# New 1,2,4-oxadiazole derivatives as potential multifunctional agents for the treatment of Alzheimer’s disease: design, synthesis, and biological evaluation

**DOI:** 10.1186/s13065-024-01235-x

**Published:** 2024-07-13

**Authors:** Mohammed Salah Ayoup, Mariam Ghanem, Hamida Abdel-Hamid, Marwa M. Abu-Serie, Aliaa Masoud, Doaa A. Ghareeb, Mohammed B. Hawsawi, Amr Sonousi, Asmaa E. Kassab

**Affiliations:** 1 Department of Chemistry, College of Science, King Faisal University, Al-Ahsa, 31982 Saudi Arabia; 2 Chemistry Department, Faculty of Science, Alexandria University, P.O. Box 426, Alexandria, 21321 Egypt; 3 Medical Biotechnology Department, Genetic Engineering and Biotechnology Research Institute, City of Scientific Research and Technological Applications (SRTA-City), Alexandria, Egypt; 4 Bio-screening and Preclinical Trial Lab, Biochemistry Department, Faculty of Science, Alexandria University, Alexandria, 21511 Egypt; 5 Center of Excellence for Drug Preclinical Studies (CE-DPS), Pharmaceutical and Fermentation Industry Development Center, City of Scientific Research & Technological Applications (SRTA-city), New Borg El Arab, Alexandria, Egypt; 6 Research Projects Unit, Pharos University in Alexandria, Alexandria, Egypt; 7 Department of Chemistry, Faculty of Science, Umm Al-Qura University, Makkah, 21955 Saudi Arabia; 8 Department of Pharmaceutical Organic Chemistry, Faculty of Pharmacy, Cairo University, Kasr El-Aini Street, Cairo, P.O. Box 11562, Cairo, Egypt; 9 University of Hertfordshire hosted by Global Academic Foundation, New Administrative Capital, Cairo, Egypt

**Keywords:** Design, Synthesis, 1,2,4-oxadiazole, Anti-alzheimer, AChE, BuChE, MAO-A, MAO-B, Molecular docking

## Abstract

A series of new 1,2,4-oxadiazole-based derivatives were synthesized and evaluated for their anti-AD potential. The results revealed that eleven compounds (**1b**, **2a-c**, **3b**, **4a-c**, and **5a-c**) exhibited excellent inhibitory potential against AChE, with IC_50_ values ranging from 0.00098 to 0.07920 µM. Their potency was 1.55 to 125.47 times higher than that of donepezil (IC_50_ = 0.12297 µM). In contrast, the newly synthesized oxadiazole derivatives with IC_50_ values in the range of 16.64**–**70.82 µM exhibited less selectivity towards BuChE when compared to rivastigmine (IC_50_ = 5.88 µM). Moreover, oxadiazole derivative **2c** (IC_50_ = 463.85 µM) was more potent antioxidant than quercetin (IC_50_ = 491.23 µM). Compounds **3b** (IC_50_ = 536.83 µM) and **3c** (IC_50_ = 582.44 µM) exhibited comparable antioxidant activity to that of quercetin. Oxadiazole derivatives **3b** (IC_50_ = 140.02 µM) and **4c** (IC_50_ = 117.43 µM) showed prominent MAO-B inhibitory potential. They were more potent than biperiden (IC_50_ = 237.59 µM). Compounds **1a**, **1b**, **3a**, **3c**, and **4b** exhibited remarkable MAO-A inhibitory potential, with IC_50_ values ranging from 47.25 to 129.7 µM. Their potency was 1.1 to 3.03 times higher than that of methylene blue (IC_50_ = 143.6 µM). Most of the synthesized oxadiazole derivatives provided significant protection against induced HRBCs lysis, revealing the nontoxic effect of the synthesized compounds, thus making them safe drug candidates. The results unveiled oxadiazole derivatives **2b**, **2c**, **3b, 4a, 4c**, and **5a** as multitarget anti-AD agents. The high AChE inhibitory potential can be computationally explained by the synthesized oxadiazole derivatives’ significant interactions with the AChE active site. Compound **2b** showed good physicochemical properties. All these data suggest that **2b** could be considered as a promising candidate for future development.

**Supplementary Information:**

The online version contains supplementary material available at 10.1186/s13065-024-01235-x.

## Introduction

The most common progressive neurodegenerative disease in the world and the main leading cause of dementia is Alzheimer’s disease (AD) [1, 2]. The two primary signs of AD are progressive memory loss and diminished intelligence [3, 4]. AD is a very difficult and emotionally exhausting condition to care for, and few diseases distress patients and their loved ones as much and regularly as it does [5]. Based on available data, AD caused an estimated 1.9 million deaths globally in 2015 [6] and an estimated 46.8 million cases of AD worldwide in 2017 [7]. With population growth, it was projected that by 2050, the number of AD patients will triple [8]. After heart disease and cancer, AD is currently the third most common cause of mortality [9]. The etiology of AD remains unclear, despite being recognized as a complex condition with numerous contributing factors explained by several theories such as the cholinergic hypothesis [10], tau hyperphosphorylation [11], amyloid cascade, and oxidative stress [12, 13]. One of the key theories explaining AD pathogenesis is the cholinergic one. Acetylcholine (ACh), a neurotransmitter, is required for the transmission of nerve impulses between muscle and/or nerve cells [14]. ACh works on the nicotinic-sensitive receptor in the central nervous system (CNS) and the muscarinic-sensitive receptor in the peripheral nervous system (PNS), which is also linked to the cardiac and smooth muscles [15]. ACh congregates in a structure known as vesicles at the terminals of neural cells. It diffuses from the vesicles into the synaptic cleft and binds to the nicotinic or muscarinic receptor, which results in the creation of electrical impulses in the cholinergic system when the action potential travels through the nerve cells and reaches the axon terminals [16]. The enzyme acetylcholine esterase (AChE), which is abundant in the synaptic clefts of both the CNS and PNS, hydrolyzes the disseminated ACh into inactive choline and acetate metabolites, shortening the half-life of ACh [17]. Additionally, research has revealed that AChE interacts to amyloid-β (Aβ) in its nonamyloidogenic form *via* peripheral active site (PAS) and promotes conformational change to its amyloidogenic form [18, 19]. The other cholinesterase (ChE) neurotransmitter, butyrylcholinesterase (BuChE), regulates the level of ACh and maintains regular cholinergic activities [20, 21]. Moreover, both ChEs and Aβ plaque deposition have been related [22, 23]. U.S. Food and Drug Administration (FDA) approved AChE inhibitors (donepezil, rivastigmine, and galantamine) that are used to treat mild-to-moderate AD stages. Simultaneously, the glutamate regulator memantine and a combination of memantine and donepezil were authorized for the treatment of moderate-to-severe AD [24]. Thus, AChE remains a potential therapeutic target in the hunt for novel anti-AD medications.

The connection between neurologic diseases and oxidative stress has garnered more attention recently. Free radicals have been linked to AD, Parkinson’s disease, head trauma, cerebral ischemia-reperfusion, and other conditions. Because the brain uses up a lot of oxygen, has a lot of lipids, and has fewer antioxidant enzymes than other tissues, it is particularly susceptible to damage from free radicals [25]. This implies that therapies for AD that try to get rid of free radicals or stop them from getting created are beneficial [26].

The breakdown of endogenous and exogenous amines is significantly aided by monoamine oxidases (MAOs), which are widely distributed enzymes. The most popular substrates for MAOs are dopamine (DA), norepinephrine (NE), epinephrine, serotonin (5-HT), and 2-phenylethylamine (PEA) [27].

The mammalian family of MAOs comprises two isozymes, MAO-A and MAO-B, which have different selectivities towards substrates and inhibitors. The A isoform preferentially deamines 5-HT and NE, while the MAO-B substrates are PEA and benzylamine [28]. It was proved that following the inhibition of MAO-B, a rise in DA levels and a neuroprotective effect are observed [29–33]. Elevated MAO-B level in aged people [34, 35] induces a rise in reactive oxygen species (ROS) and hydrogen peroxide production, which in turn may cause neuron degeneration [36–39].

Consequently, selective irreversible human MAO-B inhibitors are efficiently employed in the treatment of AD, either alone or in conjunction with other medications [40–43]. Until 2021, the FDA had only approved five medications for the treatment of AD since the disease was first identified in 1906. Therefore, there is an urgent need to discover more potent anti-AD drug candidates.

Given the complex nature of AD and recent advancements in systems biology, it was highly indicated that single-target drugs will not be adequate to treat AD or halt its progression [44]. With the ability to target several pathways involved in AD pathogenesis, multi-target-directed ligands (MTDLs) have recently attracted significant interest. The employment of this technique has resulted in the discovery of several promising anti-AD candidates [45–47].

**Fig. 1:**
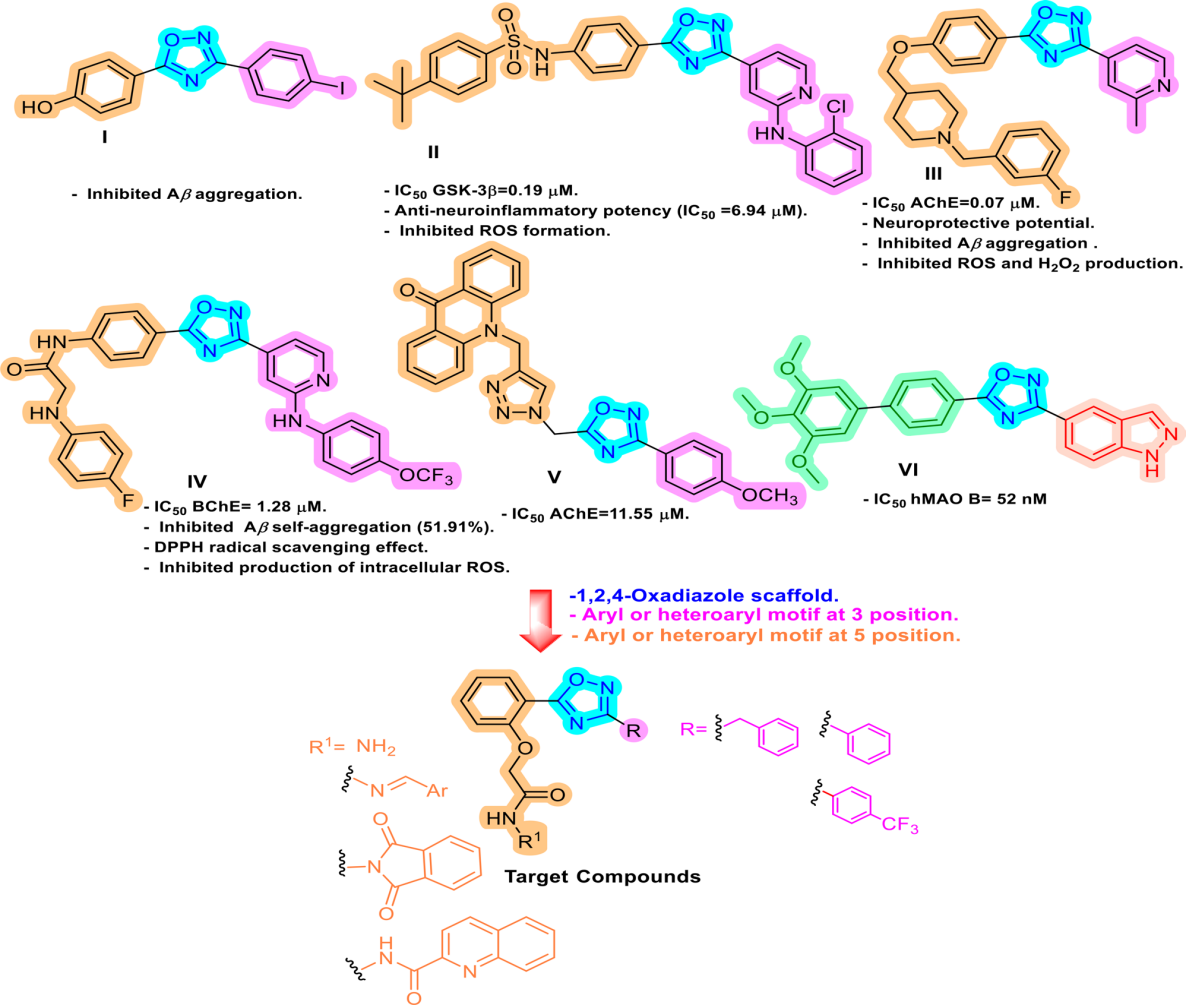
Structure of 1,2,4-oxadiazole derivatives **I**-**VI** [50–55] with anti-AD potential and design strategy for the synthesized 3,5-disubstituted-1,2,4-oxadiazole derivatives

Due to its distinct bioisosteric characteristics and a very broad range of biological activities, the five-membered 1,2,4-oxadiazole heterocyclic ring has drawn a lot of interest. It has been discovered and used as a metabolically stable analog of an ester or amide functionality in pharmacologically significant molecules [48]. Consequently, 1,2,4-oxadiazole is an ideal platform for the development of innovative drugs. Throughout the past fifteen years, there has been a two-fold increase in interest in the biological applications of 1,2,4-oxadiazoles. The FDA has approved several derivatives based on 1,2,4-oxadiazoles, which are now being promoted as commercial drugs [49]. The 3,5-disubstituted-1,2,4-oxadiazole structural motif, incorporating aryl and/or heteroaryl scaffolds as compounds **I**-**VI** (Fig. 1), is included in several anti-AD and neuroprotective candidates. They exhibited their AD potential through various mechanistic pathways such as cholinesterase inhibition and antioxidant activity. Moreover, some 1,2,4-oxadiazole derivatives demonstrated multifunctional anti-AD potential [50–55].

Our goal was to design and synthesize novel compounds based on the 1,2,4-oxadiazole scaffold, considering the previously mentioned findings (Fig. 1). The first strategy involved the incorporation of benzyl, phenyl (electron-rich moieties), or *p*-trifluoromethyl phenyl (more lipophilic and electron-poor moiety) at position 3 of the oxadiazole scaffold. In the second strategy, a phenyl moiety has been grafted to position 5 of the synthesized oxadiazole derivatives. The *ortho* position of this phenyl ring was decorated with different biologically active pharmacophoric entities, such as hydrazide or *N*-acylhydrazone. The *N*-acylhydrazone scaffold either incorporates phenyl triazole (more lipophilic and electron-rich moiety) or *o*-nitrophenyl (polar and electron-poor moiety). The bicyclic polar isoindoline motif or lipophilic quinoline motif featuring five- or six-membered heterocyclic ring fused to benzene, respectively was also introduced at the *ortho* position of the phenyl ring. Linker properties were studied in the third strategy, by bridging the aryl or heteroaryl motif at *the ortho* position of the phenyl ring at position 5 of the oxadiazole core *via* 4- or 6-atom linkers. As donors or acceptors of H bonds, these motifs and linkers may collaborate with amino acids in the active site of the AChE enzyme. Such variation in substitutions ensured different electrical, steric, and lipophilic environments, which could affect the activity of the target molecules. Several in vitro assays, such as AChE inhibition, BuChE inhibition, antioxidant activity, MAO-B inhibition, and MAO-A inhibition were performed to evaluate the synthetic oxadiazole derivatives’ potential to treat AD.

## Results and discussion

### Chemistry

In continuation of our previous work [56, 57], herein this study focuses on the functionalization of the 1,2,4-oxadiazole scaffold by constructing novel 3,5-diaryl derivatives containing phenyl, benzyl or 4-trifluoromethylphenyl at C3 position and a pharmacophoric group at C5 of the 1,2,4-oxadiazole scaffold. The phenyl moiety at the C5 position incorporates substituents at the *ortho* position with hydrogen bond donor/acceptor characters. The acetic acid hydrazide derivatives **1a-c** were prepared from the corresponding esters by refluxing with hydrazine in ethanol. Condensation of the hydrazide with *o*-nitrobenzaldehyde or 2-phenyl-*2 H*-1,2,3-triazole-4-carbaldehyde afforded the corresponding Schiff`s bases **2a-c** and **3a-c**, respectively. The structures of the Schiff`s bases were confirmed by IR and NMR spectra, where; the IR spectra showed a strong band at a wave number of 3231 to 3323 cm^− 1^, corresponding to NH stretching, also a strong band appeared at 1705 to 1716 cm^− 1^ for **2a-c** and **3a-c**, revealing the presence of the C = O group. The NMR spectra of **2a-c** and **3a-c** confirmed their existence as a mixture of *E/Z* isomers. Where **2a** and 2**c** exist in a ratio (5:2) and **2b**, **3a**, and **3c** exist in a ratio (2:1), while **3b** exists in a ratio (3:2) for *E/Z*, respectively. The ^1^HNMR spectra of **2a-c** and **3a-c** showed the proton of N**H** resonating downfield at *δ*_*H*_: 11.94 to 11.68 ppm for the major and minor isomers. Furthermore, the ^13^CNMR spectra showed a characteristic signal for ArO**C**H_2_ resonating at *δ*_*C*_: range 65.9 to 66.1 ppm. Furthermore, refluxing of the hydrazides **1a-c** with phthalic anhydride in acetic acid afforded the corresponding *N*-Phthalimido-protected hydrazide **4a-c**. The structure of **4a-c** was confirmed by IR spectra, where the N**H** group appeared at wave number 3247 to 3182 cm^− 1^, corresponding to N**H** stretching. A strong band appeared at the range 1739 –1737 cm^− 1^, corresponding to the C = O group. The ^1^HNMR spectral analyses confirmed the structure of **4a-c**, where the N**H** proton appeared at range *δ*_*H*_: 11.08–11.01 ppm, also the aliphatic protons of ArOC**H**_2_ for **4a-c** appeared at *δ*_*H*_: 5.14, 5.06, and 5.13 ppm, respectively. Moreover, ^13^CNMR spectra of **4a-c** showed the characteristic signals corresponding to the carbonyl carbon of the phthalimido group at *δ*_*C*_: 165.38, 165.1, and 165.31 ppm, respectively. Coupling of the hydrazide **1a, b** with quinaldic acid using DCC/6-NO_2_-HOBt protocol afforded the amide products **5a, b** in excellent yields. On the other hand, the amide **5c** was synthesized in a good yield superior to that of the DCC/NO_2_-HOBt protocol *via* a reaction of the acid chloride of quinaldic acid and the hydrazide **1c**. The structures of **5a-c** were confirmed by IR spectra, where the N**H** group appeared at wave number 3435 to 3255 cm^− 1^, corresponding to N**H** stretching. A strong band appeared at the range 1719 –1666 cm^− 1^, corresponding to the C = O groups of the amides. The ^1^HNMR spectral analyses confirmed the structure of **5a-c**, where the N**H** proton appeared at range *δ*_*H*_: 10.96–10.15 ppm, also the aliphatic protons of ArOC**H**_2_ of compounds **5a-c** appeared at *δ*_*H*_: 4.88, 4.82, and 5.0 ppm, respectively. Moreover, ^13^CNMR spectra of **5a-c** showed the characteristic signals corresponding to the carbonyl carbon of amide at *δ*_*C*_: 167.1 to 169.9 ppm. The characteristic carbon of CF_3_ of **5c** appeared at *δ*_*C*_: 126.4 ppm.

**Scheme 1:**
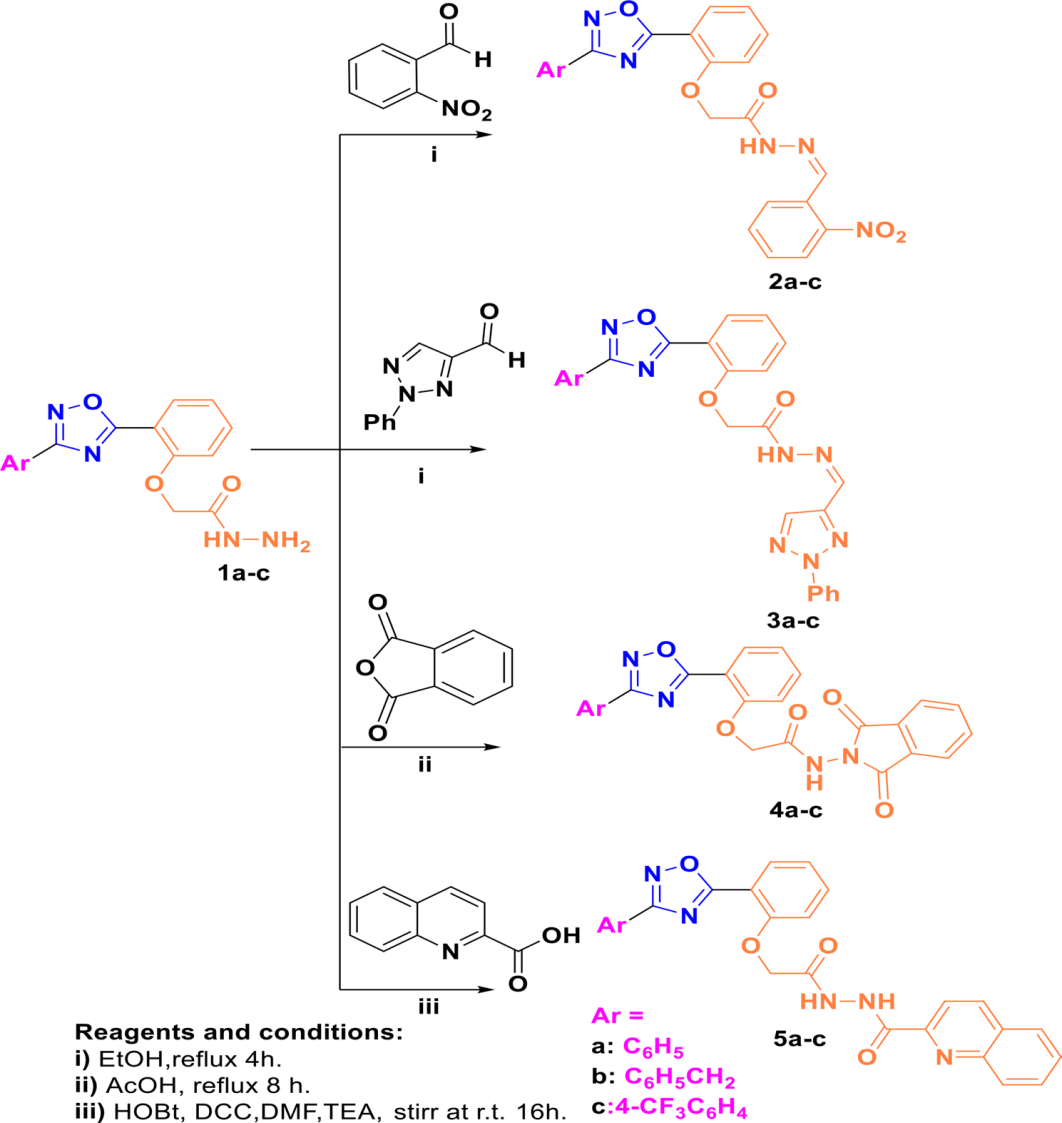
The reagents and synthesis route used to prepare the target compounds **2–5**

## **AChE and BuChE inhibitory activities**

**Fig. 2:**
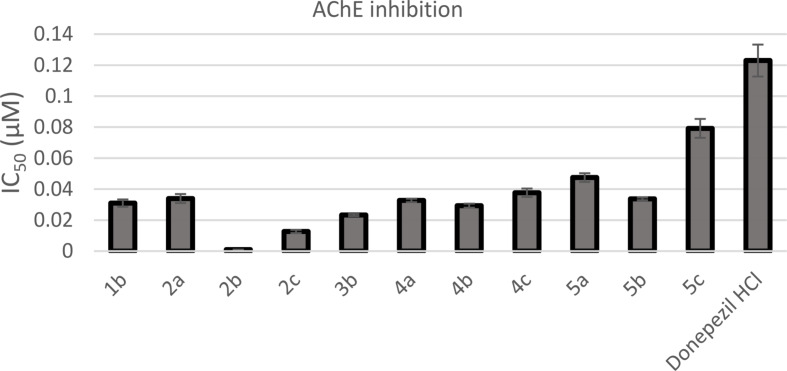
A bar diagram representing the AChE inhibition of the synthesized compounds

**Fig. 3:**
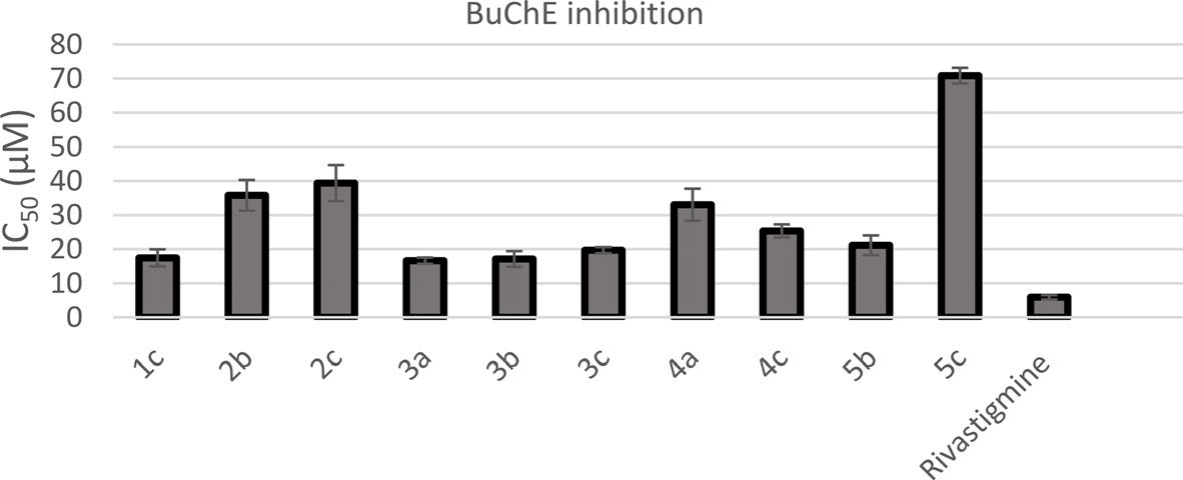
A bar diagram representing the BuChE inhibition of the synthesized compounds

The in vitro AChE and BuChE inhibition potentials of newly synthesized oxadiazole derivatives were assessed by comparing their half maximum inhibitory concentration (IC_50_) values to those of anti-AD drugs, donepezil and rivastigmine. The results, which are shown in Table 1, are graphically represented in Figs. 2 and 3. Eleven compounds (**1b**, **2a-c**, **3b**, **4a-c**, and **5a-c**) exhibited excellent inhibitory potential against AChE, with IC_50_ values ranging from 0.00098 to 0.07920 µM. Their potency was 1.55 to 125.47 times higher than that of donepezil (IC_50_ = 0.12297 µM). Oxadiazole derivatives **1a**, **1c, 3a**, and **3c** exhibited nonexistent efficacy towards AChE. In contrast, the newly synthesized oxadiazole derivatives with IC_50_ values in the range of 16.64**–**70.82 µM exhibited less selectivity towards BuChE when compared to rivastigmine (IC_50_ = 5.88 µM). Compounds **3a**, **3b** and **1c** showed the most prominent inhibitory potential against BuChE with IC_50_ values of 16.64, 17.14, and 17.74 µM, respectively.

**Table 1:** Biological evaluation results of the synthesized 1,2,4-oxadiazole-based derivatives

Compound	AChE-IC_50_ (µM ± SD) ^a^	BuChE-IC_50_ (µM ± SD) ^a^	AChE Selectivity Index ^b^	DPPH-IC_50_ (µM ± SD) ^a^	MAO-B-IC_50_ (µM ± SD) ^a^	MAO-A-IC_50_ (µM ± SD) ^a^	Hemolysis-IC_50_ (µM ± SD) ^a^
1a	N.A.	N.A.	-	1215.74 **±** 51.30	358.61 ± 23.4	**47.25 ± 8.1**	**1620.36 ± 57.8**
1b	**0.03098 ± 0.0024**	N.A.	-	1280.92 **±** 42.80	534.58 ± 41.5	**82.54 ± 8.7**	**1193.47 ± 78.3**
1c	N.A.	17.47 ± 2.5	-	734.85 ± 12.80	396.84 ± 19.6	146.63 ± 16.9	656.56 ± 33.1
2a	**0.03393 ± 0.0029**	N.A.	-	1306.54 ± 31.50	N.A.	N.A.	**1792.14 ± 63.1**
2b	**0.00098 ± 0.000001**	35.84 ± 4.5	36,571	991.45 ± 11.40	346.03 **±** 14.3	203.91 ± 17.9	**4104.89 ± 114.5**
2c	**0.01271 ± 0.00102**	39.41 ± 5.3	3,100	**463.85 ± 31.50**	265.56 ± 13.4	N.A.	**4338.69 ± 125.6**
3a	N.A.	16.64 ± 0.93	-	N.A.	N.A.	**85.2 ± 5.9**	622.32 **±** 23.4
3b	**0.02330 ± 0.00069**	17.14 ± 2.3	735	536.83 ± 19.30	**140.02 ± 8.7**	N.A.	457.23 **±** 19.6
3c	N.A.	19.72 ± 0.89	-	582.44 **±** 42.90	N.A.	**114.6 ± 19.5**	661.47 **±** 23.8
4a	**0.03275 ± 0.0011**	33.07 ± 4.7	1,010	1658.66 **±** 62.30	393.68 ± 16.3	N.A.	522.45 **±** 15.9
4b	**0.02934 ± 0.0013**	N.A.	-	2660.83 ± 26.30	713.28 **±** 22.3	**129.7 ± 16.9**	**1788.84 ± 89.3**
4c	**0.03769 ± 0.0027**	25.38 ± 1.9	673	N.A.	**117.43 ± 9.8**	143.9 ± 22.4	1040.07 **±** 88.9
5a	**0.04748 ± 0.0028**	N.A.	-	885.05 **±** 22.90	274.43 **±** 21.1	N.A.	**2016.04 ± 111.3**
5b	**0.03374 ± 0.00089**	21.16 ± 2.9	627	N.A.	N.A.	N.A.	**3559.75 ± 125.3**
5c	**0.07920 ± 0.0061**	70.82 ± 2.3	894	1143.46 ± 68.90	N.A.	N.A.	521.22 ± 11.9
Donepezil HCl	0.12297 ± 0.0.0103						
Rivastigmine		5.88 **±** 0.64					
Quercetin				491.23 ± 14.8			
Biperiden HCl					237.59 ± 16.3		
Diclofenac							1121.94 ± 12.6
Methylene blue						143.6 ± 22.1	

^**a**^ The means of three replicates are used to express all values

^b^ AChE Selectivity index = IC_50_ of BuChE/ IC_50_ of AChE

The IC_50_ values of compounds that are more powerful than the reference standard are shown in the bold text

N.A. means no activity

## 1,1-Diphenyl-2-picrylhydrazyl (DPPH) radical scavenging activity

**Fig. 4:**
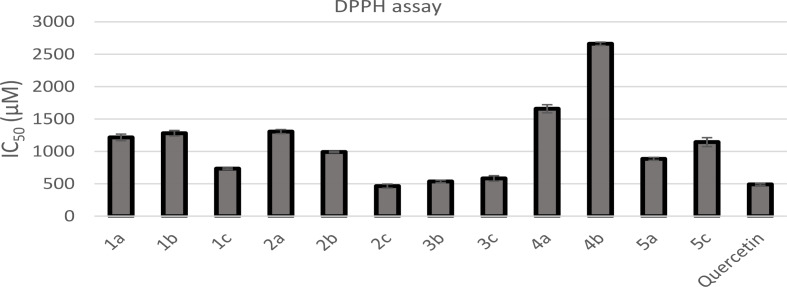
A bar diagram representing the antioxidant potential of the synthesized compounds using DPPH assay

The antioxidant activity of the synthesized oxadiazole derivatives was evaluated using the DPPH assay. Quercetin, a common antioxidant, was used for comparison of the IC_50_ values. As can be shown in Table 1; Fig. 4, among tested oxadiazole derivatives, *N*-acylhydrazone derivative **2c** (IC_50_ = 463.85 µM) was a more potent antioxidant than quercetin (IC_50_ = 491.23 µM). *N*-Acylhydrazone derivatives **3b** (IC_50_ = 536.83 µM) and **3c** (IC_50_ = 582.44 µM) exhibited comparable antioxidant activity to that of quercetin. Compounds **1c**, **2b**, and **5a** (IC_50_ values of 734.85, 991.45, and 885.05 µM, respectively) showed significant antioxidant potential. With IC_50_ values in the range of 1143.46- 1306.54 µM, oxadiazole derivatives **1a**, **1b**, **2a**, and **5c** exhibited moderate antioxidant activity.

## MAO-B and MAO-A inhibitory activities

**Fig. 5:**
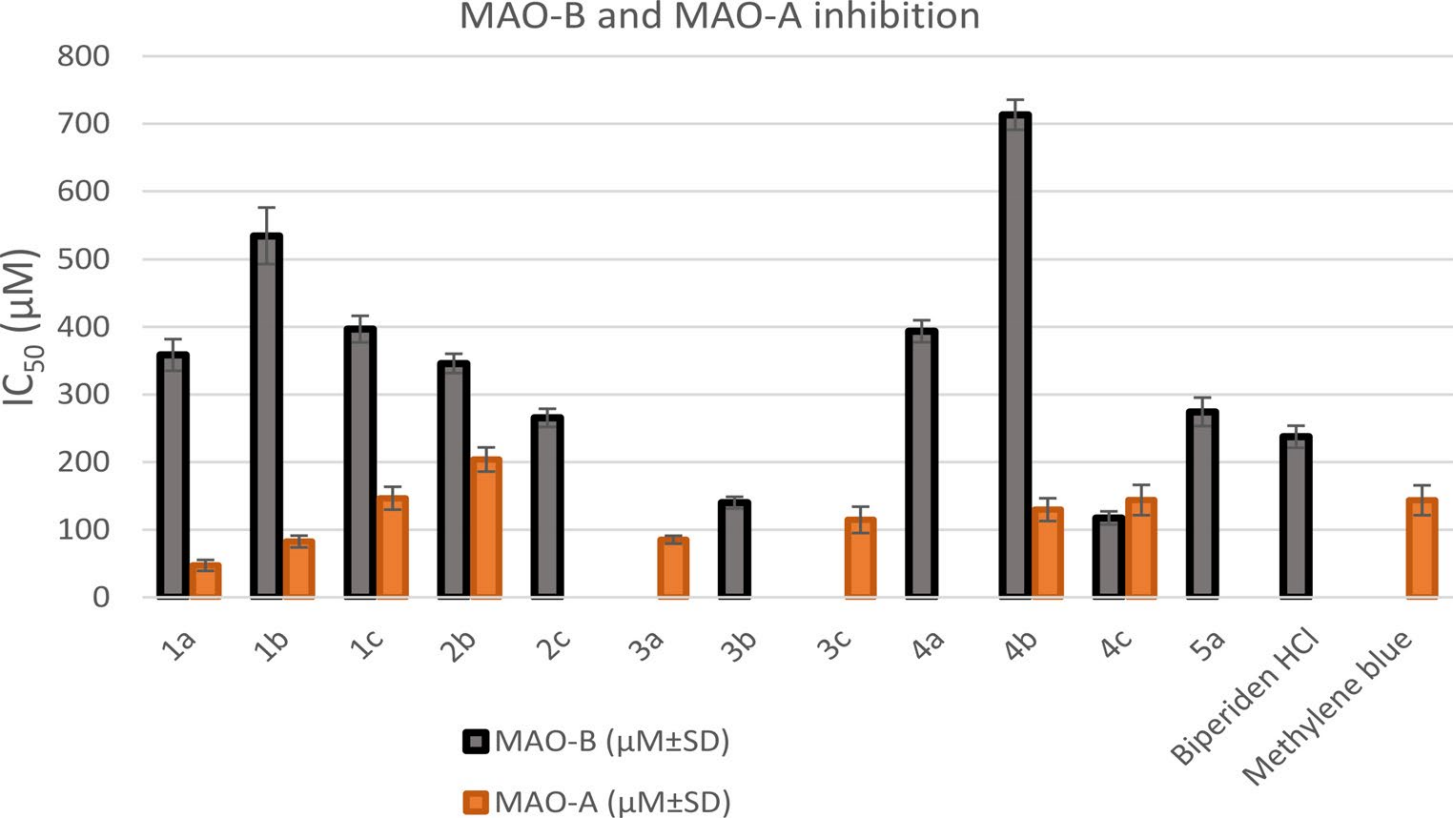
A bar diagram representing the MAO-B and MAO-A inhibition of the synthesized compounds

The MAO-B and MAO-A inhibitory potentials of all the synthesized 1,2,4-oxadiazole-based derivatives were evaluated. Biperiden and methylene blue (methylthioninium chloride) were used as the reference standards. As shown in Table 1; Fig. 5, oxadiazole derivatives **3b** (IC_50_ = 140.02 µM) and **4c** (IC_50_ = 117.43 µM) showed excellent MAO-B inhibitory potentials. They were more potent than biperiden (IC_50_ = 237.59µM). Compounds **2c** and **5a** with IC_50_ values of 265.56 and 274.43 µM, respectively, exhibited equipotent MAO-B inhibitory activity to that of biperiden. Oxadiazole derivatives **1a**, **1c**, **2b**, and **4a** with IC_50_ values in the range of 346.03–396.84 µM showed significant MAO-B inhibitory activity. Five oxadiazole derivatives (**1a**, **1b**, **3a**, **3c**, and **4b**) exhibited remarkable MAO-A inhibitory potential, with IC_50_ values ranging from 47.25 to 129.7 µM. Their potency was 1.1 to 3.03 times higher than that of methylene blue (IC_50_ = 143.6 µM). Compound **4c** (IC_50_ = 143.9 µM) exhibited equipotent MAO-A inhibitory potential to that of methylene blue.

## In vitro determination of the anti-hemolytic effect of synthesized compounds

**Fig. 6:**
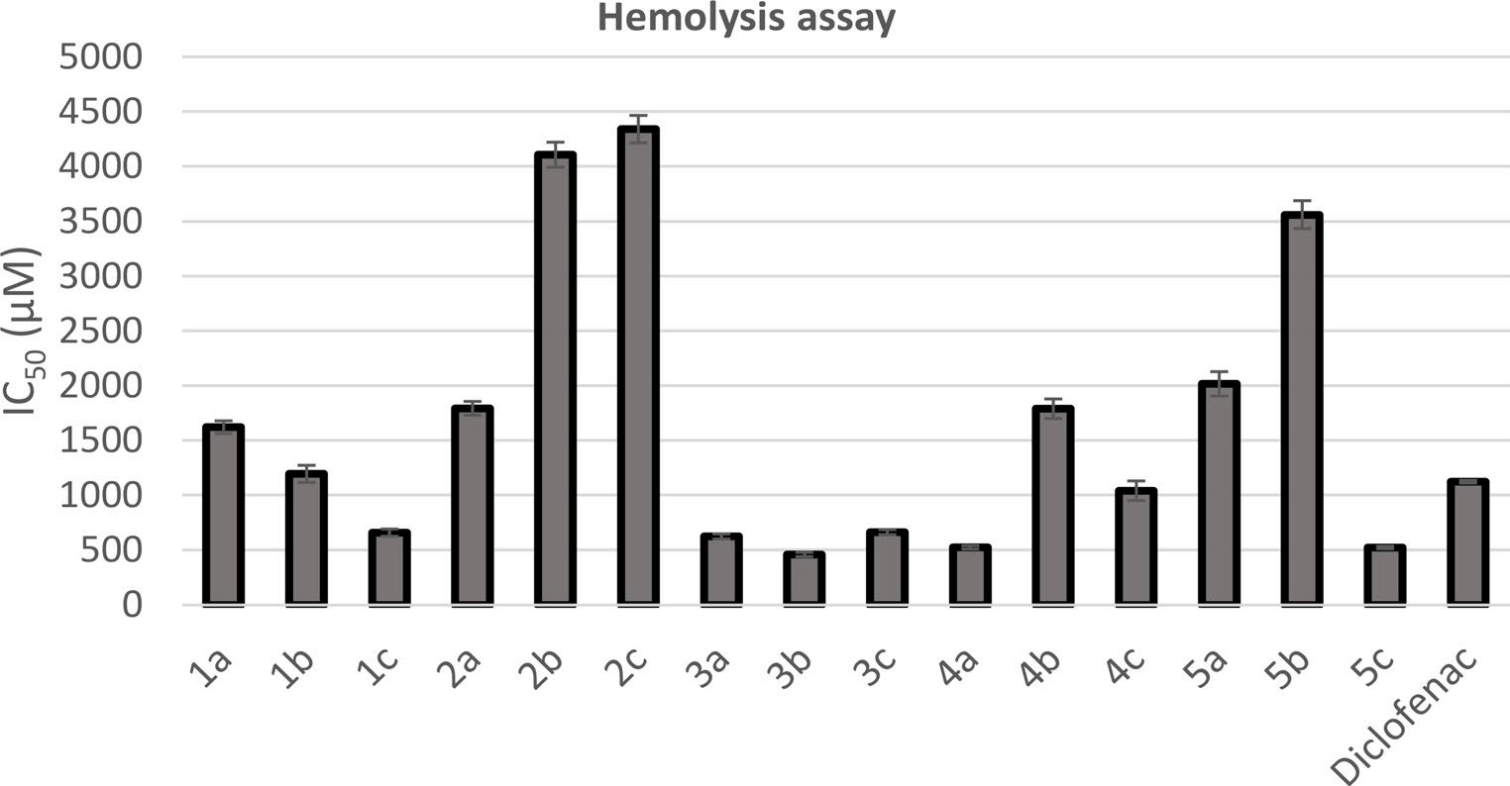
A bar diagram representing the anti-hemolytic effect of the synthesized compounds

The inhibition of human red blood cells (HRBCs) membrane lysis under hypnotic conditions was taken as a measure of the mechanism of anti-hemolytic activity of the synthesized 1,2,4-oxadiazole-based derivatives. Inhibition of hemolysis was expressed as IC_50_ value, which is the inhibitory concentration at which 50% of hemolysis is repressed. Eight compounds namely, **1a**, **1b**, **2a-c**, **4b**, **5a**, and **5b** with IC_50_ values at the range of 1193.47–4338.69 µM (Table 1), provided higher protection against induced lyses than diclofenac (IC_50_ = 1121.94 µM). Compound **4c** (IC_50_ = 1040.07 µM), exhibited protection against induced lyses comparable to that of diclofenac. The results revealed the nontoxic effect of the synthesized 1,2,4-oxadiazole derivatives, thus making them safe drug candidates (Fig. 6).

## Structure-activity relationships (SAR)

**Fig. 7:**
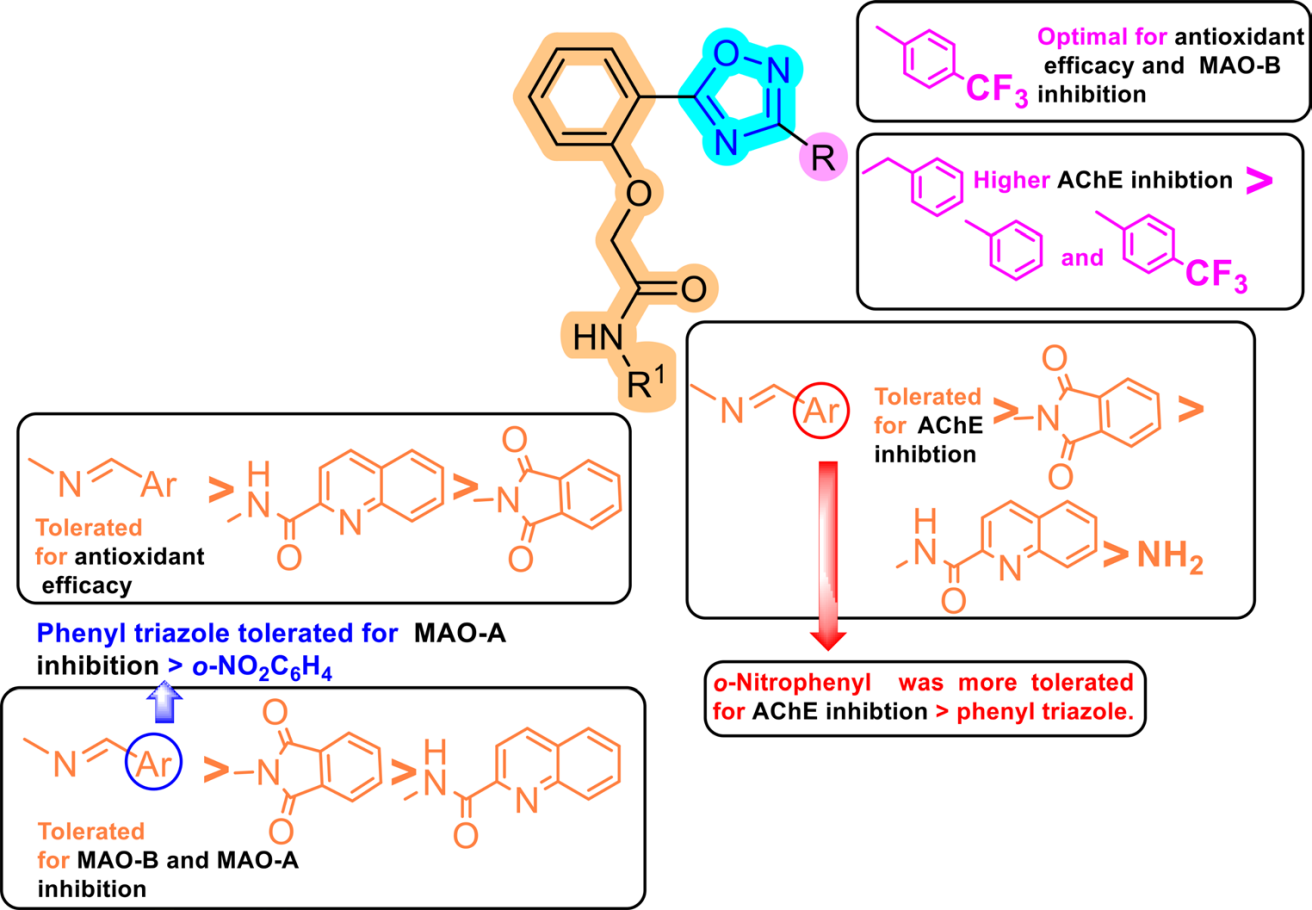
Structure-activity relationship (SAR) for anti-AD potential of 1,2,4-oxadiazole-based derivatives

The structure-activity relationship studies of the new oxadiazole-based derivatives revealed that the 3,5-disubstituted-1,2,4-oxadiazole pharmacophoric entity is highly tolerated for AChE inhibition. It was clear that grafting a benzyl moiety at position 3 of the oxadiazole ring (**1b**, **2b**, **3b**, **4b**, and **5b**) exhibited higher AChE inhibitory activity than phenyl and *p*-trifluoromethylphenyl moieties. It was intriguing to observe that among oxadiazole derivatives featuring the hydrazide scaffold at the *ortho* position of the phenyl ring of the 5th position of the oxadiazole ring, only compound **1b** incorporating the benzyl moiety showed potent AChE inhibitory activity. Shifting the hydrazide moiety into the *N*-acylhydrazone scaffold improved the AChE inhibitory potential. Further analysis of hydrazone derivatives revealed that the polar and electron-poor *o*-nitrophenyl motif (**2a-c**) was more tolerated for AChE inhibition than the lipophilic and electron-rich phenyl triazole motif (**3a-c**). The incorporation of isoindoline or quinoline scaffold into the hydrazide moiety demonstrated a good impact on the AChE inhibition. An interesting phenomenon is that polar isoindoline derivatives (**4a-c**) scored higher AChE inhibitory potential than lipophilic quinoline counterparts (**5a-c**) (Fig. 7).

Regarding the antioxidant potential, it was concluded that the 1,2,4-oxadiazole derivatives incorporating the *N*-acylhydrazone scaffold (**2** and **3**) emerged as the most prominent antioxidant candidates. In hydrazide and hydrazone derivatives, grafting the lipophilic electron-deficient *p*-trifluoromethylphenyl at position 3 of the oxadiazole ring (**1c**, **2c**, and **3c**) exhibited significant antioxidant potential. The lipophilic quinoline motif displayed more efficient antioxidant activity than the polar isoindoline motif (Fig. 7).

The 1,2,4-oxadiazole hydrazide derivatives (**1a-c**) showed mild MAO-B inhibition. The incorporation of *N*-acylhydrazone scaffold (**2b**, **2c**, and **3b**) showed a significant impact on the MAO-B inhibition. It was noteworthy that MAO-B inhibition was more enhanced by the incorporation of the isoindoline motif (**4a-c**) than the quinoline motif (**5a-c**). It is worth noting that inserting the lipophilic electron-deficient *p*-trifluoromethylphenyl at position 3 of the oxadiazole ring (**1c**, **2c**, and **4c**) highly improved MAO-B inhibitory potential (Fig. 7).

Regarding MAO-A inhibition, both hydrazide and *N*-acylhydrazone scaffolds were tolerated for activity. The incorporation of an electron-rich phenyl triazole motif in the *N*-acylhydrazone scaffold (**3a** and **3c**) exhibited a better impact on MAO-A inhibition than the electron-poor *o*-nitrophenyl motif (**2a-c**). An interesting phenomenon was that MAO-A inhibition was enhanced by the incorporation of the isoindoline motif (**4b** and **4c**), in contrast, adding the quinoline moiety in compounds **5a-c** completely abolished MAO-A inhibitory potential.

## Molecular docking study

**Fig. 8:**
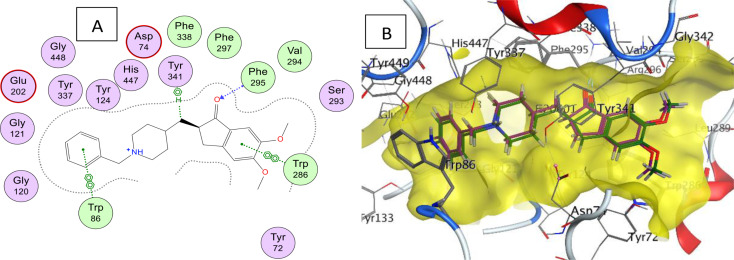
**A**) 2D diagram of donepezil interaction with human acetylcholinesterase; **B**) 3D of the co-crystallized donepezil inhibitor (magenta) together with the re-docked donepezil (green) in the gorge of the active site (highlighted in yellow) of human acetylcholinesterase (RMSD = 0.42 Å)

The purpose of the docking study is to predict the binding interactions between the synthesized drug candidates and the active site of AChE enzyme as well as to rationalize the biological activities of the target molecules. MOE (2020.09) software [58] was used in this study. First, the protein crystal structure of AChE (PDB: 7E3H) [59] was downloaded from the protein data bank and the docking procedure was validated step before docking the synthesized molecules. In the validation step, the co-crystallized molecule (donepezil) was docked in the active site of AChE enzyme to get a pose that overlapped the experimental pose with RMSD of 0.42 Å which is within the cutoff limit (< 1 Å) (Fig. 8). The active site of AChE consists of a narrow groove that is 20 Å deep and contains the catalytic site deep at the bottom of the groove. The donepezil structure is deeply embedded inside the AChE active site pocket and the 2,3-dihydroinden-1-one carbonyl moiety interacts with Phe295 by hydrogen bonding. The benzyl group showed arene interactions with Trp86 amino acid of the enzyme. The synthesized molecules were docked using the same method and the binding modes with the human AChE binding site were evaluated. The compounds generally displayed analogous interactions and occupied the same space with the binding site as donepezil.

**Fig. 9:**
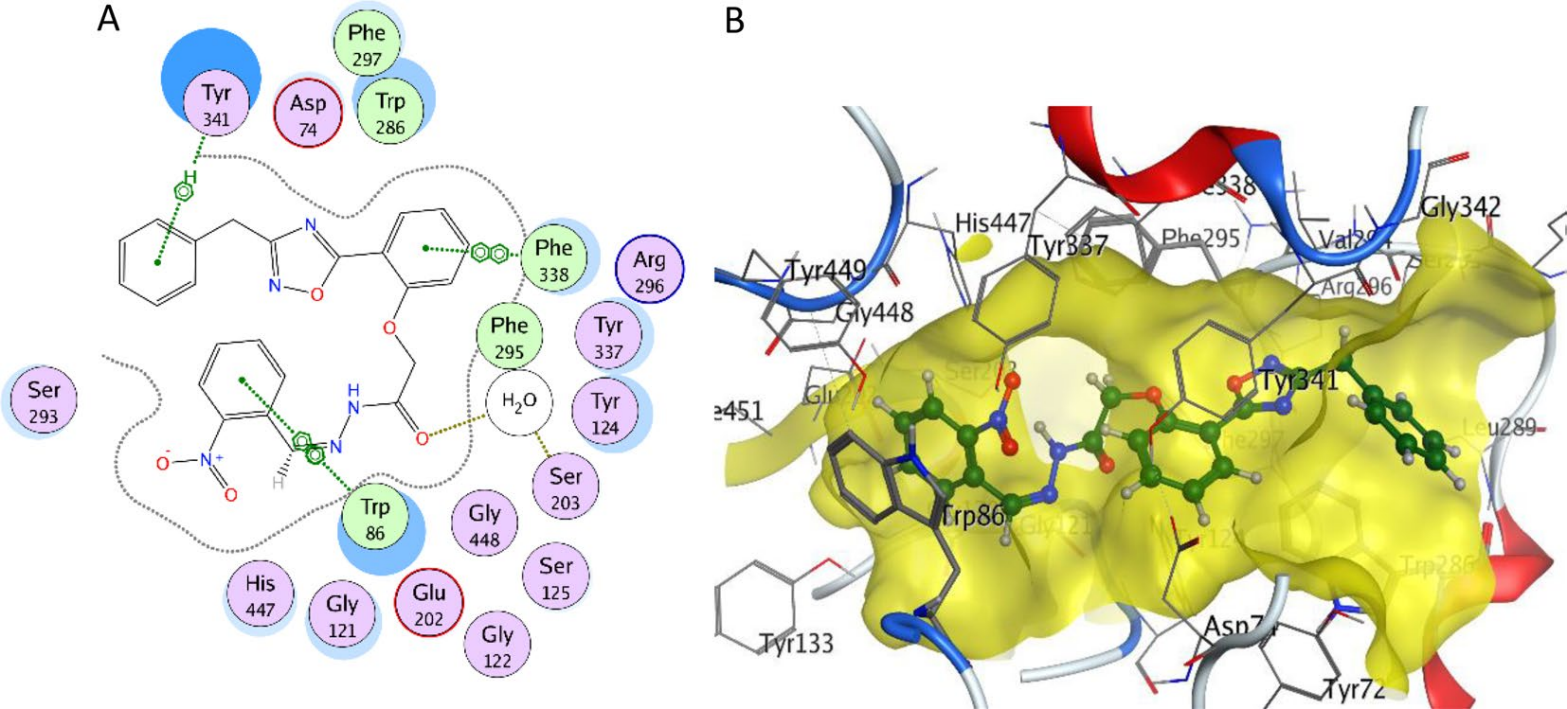
**A**) 2D diagram and **B**) 3D diagram of the interactions of compound **2b** (green) with the AChE active site (highlighted in yellow) (PDB: 7E3H)

The 3-benzyl-1,2,4-oxadiazole moiety of compound **2b** inhabited a similar space as donepezil piperidine ring and formed Arene-H interaction with Tyr341 (Fig. 9). In addition, the hydrazide carbonyl forms a hydrogen bond with Ser203 *via* a water bridge which improved the stability of the molecule in the active site. The 5-phenyl moiety formed Arene-H interaction with Phe338. Finally, the nitro phenyl group showed Arene-H interaction with Trp86 same as donepezil. Most compounds with benzyl substitution showed better interactions and higher binding energies than their phenyl and trifluoro phenyl congeners which rationalize their higher inhibition activity towards AChE enzyme. In addition, all compounds with benzyl substitution showed Arene-H interaction with Tyr341 which may highlight the importance of this interaction with binding stability.

**Fig. 10:**
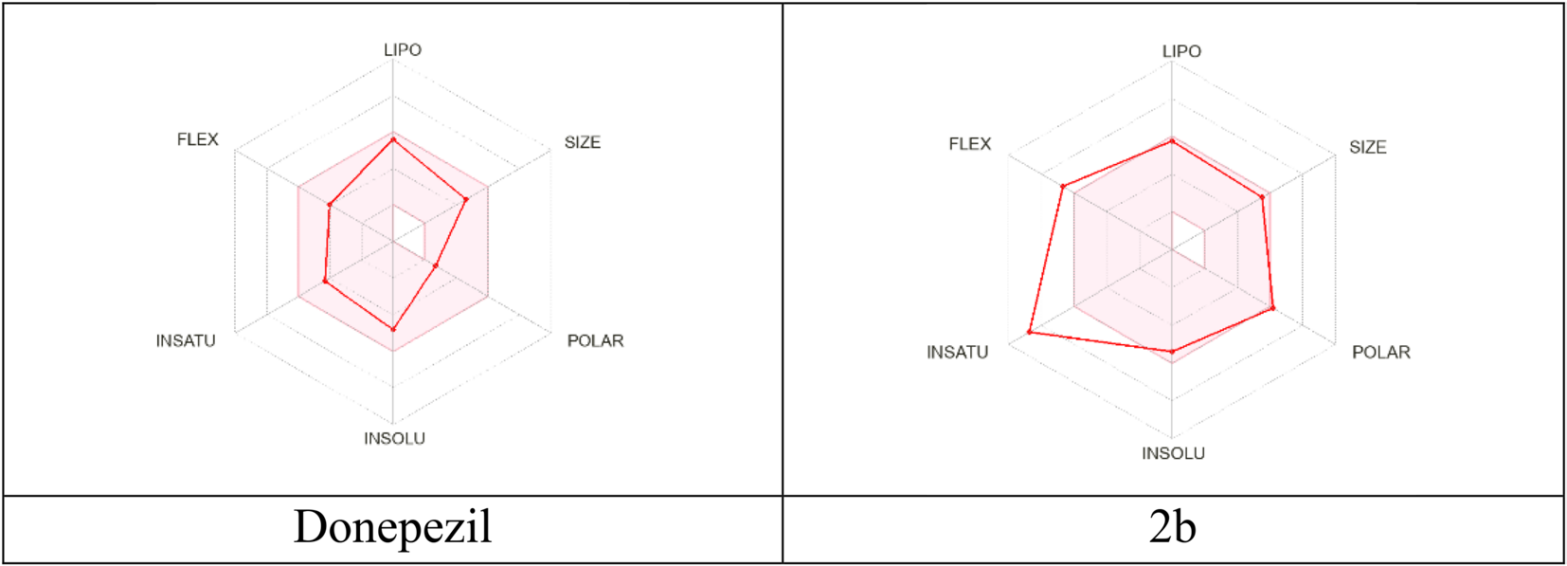
Bioavailability radar map of donepezil and **2b**. The inner pink area shows the ideal range for each of the following characteristics: solubility: log S should not exceed 6, saturation: the fraction of carbons in the sp^3^ hybridization should not be less than 0.25, flexibility: no more than 9 rotatable bonds, polarity: TPSA between 20 and 130 Å^2^, size: molecular weight between 150 and 500 g/mol, and lipophilicity: XLOGP3 between − 0.7 and + 5.0

In addition, the prediction of several properties such as ADME and other physicochemical properties was done using the online software SwissADME. All compounds showed alignment with Lipinski’s rule of five [60]. Most of the compounds showed a high probability of gastrointestinal absorption and all of them exhibited a 0.55 oral bioavailability score (see Supplementary data) and the docking score and interactions of all target compounds with the active site of AChE enzyme were collected in (Table 2) with donepezil. Compound **2b** showed the highest in vitro inhibitory activity, high docking binding, and good physicochemical properties as shown in the bioavailability radar map (Fig. 10). All these data suggest that **2b** could be considered as a promising candidate for future development.

**Table 2:** The docking score and interactions of all target compounds

Compound	Docking score with human AChE active site (PDB: 7E3H) (kcal/mol)	Interactions with human AChE active site
**1a**	-7.655	HB (Asn87) HB (Tyr124)
**1b**	-7.895	HB (Asp74) HB (Trp86, *via* water bridge) Arene-H (Tyr341)
**1c**	-7.805	HB (Asn87) HB (Tyr124)
**2a**	-8.808	HB (Ser203, *via* water bridge) Arene-arene (Trp286) Arene-arene (Tyr124)
**2b**	-8.853	HB (Ser203, *via* water bridge) Arene-H (Tyr341) Arene-arene (Phe338) Arene-arene (Trp86)
**2c**	-8.391	Arene-arene (Trp86)
**3a**	-8.674	HB (Tyr124) Arene-arene (Trp286) Arene-H (Trp86)
**3b**	-8.723	Arene-H (Tyr341) Arene-H (Tyr337) Arene-arene (Trp86) Arene-H (Ser203, *via* water bridge)
**3c**	-8.691	HB (Phe295) Arene-H (Trp286) Arene-H (Ser203, *via* water bridge)
**4a**	-9.314	HB (Trp86, *via* water bridge) Arene-arene (Trp86) Arene-arene (Tyr341)
**4b**	9.122	HB (Trp86, *via* water bridge) HB (Thr83, *via* water bridge) Arene-arene (Trp86) Arene-arene (Tyr341) Arene-H (Tyr337)
**4c**	-9.067	Arene-arene (Tyr341) Arene-arene (Trp86) Arene-arene (Trp286)
**5a**	-8.650	Arene-H (Tyr341) Arene-H (Gly121) Arene-arene (Trp86)
**5b**	-8.675	Arene-H (Tyr341) Arene-H (Gly121) Arene-arene (Trp86) Arene-H (Ser203, *via* water bridge)
**5c**	-8.598	HB (Asp74) Arene-arene (Trp86) Arene-H (Ser203, *via* water bridge)
**Donepezil HCl**	-9.592	HB (Phe295) Arene-H (Tyr341) Arene-arene (Trp86) Arene-arene (Trp286)

## Conclusion

New derivatives based on 1,2,4-oxadiazole core were designed, synthesized and their anti-AD potential was assessed. Eleven compounds with IC_50_ values ranging from 0.00098 to 0.07920 µM demonstrated excellent inhibitory potential against AChE. Their efficacy was 1.55 to 125.47 times greater than donepezil’s. In contrast, the newly synthesized oxadiazole derivatives exhibited less selectivity towards BuChE. Furthermore, compared to quercetin (IC_50_ = 491.23 µM), oxadiazole derivative **2c** (IC_50_ = 463.85 µM) exhibited greater antioxidant capacity. Comparable antioxidant activity to that of quercetin was shown by compounds **3b** (IC_50_ = 536.83 µM) and **3c** (IC_50_ = 582.44 µM). The oxadiazole compounds with the highest ability to inhibit MAO-B were **3b** (IC_50_ = 140.02 µM) and **4c** (IC_50_ = 117.43 µM). Compared to biperiden (IC_50_ = 237.59 µM), they exhibited more potent MAO-B inhibition. Oxadiazole derivatives **1a**, **1b**, **3a**, **3c**, and **4b** exhibited excellent MAO-A inhibitory potential. Their potency was 1.1 to 3.03 times higher than that of methylene blue. Compound **4c** exhibited equipotent MAO-A inhibitory potential to that of methylene blue. The majority of synthesized oxadiazole derivatives demonstrated a strong protective effect against induced lysis of human red blood cells, indicating their safety as potential therapeutic options. Oxadiazole derivatives **2b**, **2c**, **3b**, **4a**, **4c**, and **5a** were shown as multitarget anti-AD agents. A computational explanation for the high AChE inhibitory potential is the strong interactions between the synthesized oxadiazole derivatives and the AChE active site. The physicochemical properties of compound **2b** were prominent. 1,2,4-Oxadiazole derivative **2b** is a promising anti-AD drug candidate for further research and development.

## Experimental

### Chemistry

#### Materials and equipment

The materials and equipment were reported in the Supporting Information section.

#### General method for the synthesis of Schiff`s base 2a-c and 3a-c

To a stirred solution of the acid hydrazide **1a-c**, (0.318 mmol) in ethanol (20 mL) o-nitrobenzaldehyde or 2-phenyl-2 H-1,2,3-triazole-4-carbaldehyde (0.35 mmol) was added and the reaction mixture was heated under reflux for 9 h, then left to cool to room temperature. The solid formed was filtered off to give the desired product.

**(*E*/*Z*)-*N*’-(2-Nitrobenzylidene)-2-(2-(3-phenyl-1,2,4-oxadiazol-5-yl)phenoxy)acetohydrazide (2a)**.

Off-white powder; (yield 65.0%); m.*p* = 190–191 ˚C; Rf = 0.1 (n-hexane: EtOAc, 2:1); IR (KBr, cm^− 1^): 3317 (NH), 1714 (CO), 1526, 1306 (NO_2_); NMR for the major product (**2a**-*E isomer*): ^1^H NMR (500 MHz, DMSO-*d*_*6*_) *δ*_*H*_: 11.94 (s, 0.67 H, N**H**), 8.35 (s, 0.67 H, C**H =** N), 8.11–7.139 (m, 13H, Ar**H**), 5.383 (s, 1.37 H, ArOC**H**_2_); ^13^C NMR (125 MHz, DMSO-*d*_*6*_) *δ*_*C*_: 175.7, 169., 168.1, 157.8, 148.4, 144.1, 140, 135.1, 134.1, 132.1, 131.1, 129.8, 129.1, 128.6, 127.6, 126.9, 125.1, 121.7, 114.5, 113.1, 65.9; Anal. calcd for C_23_H_17_N_5_O_5_ (443.12): C, 62.30; H, 3.86; N, 15.79; Found: C, 62.66; H, 3.91; N, 15.59.

##### (*E*/*Z*)-2-(2-(3-Benzyl-1,2,4-oxadiazol-5-yl)phenoxy)-*N*’-(2-nitrobenzylidene)acetohydrazide (2b)

White powder; (yield 72.0%); m.*p* = 184–186 ˚C; Rf = 0.1 ( n-hexane: EtOAc, 2:1); IR (KBr, cm^− 1^): 3231 (NH), 1705 (CO), 1517 and 1287 (NO_2_); NMR for the major product (**2b**, *E-isomer*): ^1^HNMR (500 MHz, DMSO-*d*_*6*_) *δ*_*H*_: 11.92 (s, 0.66 H, N**H**), 8.34 (s, 0.67 H, C**H =** N), 8.11–7.139 (m, 13H, Ar**H**), 5.34 (s, 1.1 H, ArOC**H**_2_), 4.13 (d, *J* = 8.5 Hz, 2 H, ArC**H**_**2**_); ^13^C NMR (125 MHz, DMSO-*d*_*6*_) *δ*_*C*_: 175.3, 169.7, 169.4, 157.6, 148.5, 144.1, 140.0, 136.5, 134.9, 134.1, 131.7, 131.1, 129.5, 129.1, 128.6, 127.4, 125.1, 121.6, 114.4, 113.1, 65.9, 31.9; Anal. calcd for C_24_H_19_N_5_O_5_ (457.14), C, 63.02; H, 4.19; N, 15.31; Found: C, 62.91; H, 4.32; N, 15.24.

##### (*E*/*Z*)-*N*’-(2-Nitrobenzylidene)-2-(2-(3-(4-(trifluoromethyl)phenyl)-1,2,4-oxadiazol-5-yl)phenoxy) acetohydrazide (2c)

White powder; (yield, 61.0%) m.*p* = 222–224 ˚C; Rf = 0.1 ( n-hexane: EtOAc, 2:1); IR (KBr, cm^− 1^): 3315 (NH), 1716 (CO), 1531 and 1322 (NO_2_); NMR for the major product ( *E-isomer*) product: ^1^H NMR (500 MHz, DMSO-*d*_*6*_) *δ*_*H*_: 11.94 (s, 0.61 H, N**H**), 8.36 (s, 0.63 H, C**H =** N), 8.28–7.15 (m, 12 H, Ar**H**), 5.395 (s, 1.32 H, ArOC**H**_2_); ^13^C NMR (125 MHz, DMSO-*d*_*6*_) *δ*_*C*_: 176.1, 169.3, 167.2, 164.7, 157.8, 148.5, 144.1, 140.0, 135.3, 134.1, 131.9, 131.1, 130.7, 129.1, 128.7, 128.5, 126.8, 125.1, 121.8, 114.6, 112.9, 66.1; Anal. calcd for C_24_H_16_F_3_N_5_O_5_ (511.11), C, 56.37; H, 3.15; N, 13.69; Found: C, 56.51; H, 3.35; N, 13.43.

##### *(E*/*Z*)-2-(2-(3-Phenyl-1,2,4-oxadiazol-5-yl)phenoxy)-N’-((2-phenyl-2 H-1,2,3-triazol-4-yl)methylene) acetohydrazide (3a)

White powder (yield 73.0%); m.*p* = 224–228 ˚C; Rf = 0.33 ( n-hexane: EtOAc, 2:1); IR (KBr, cm^− 1^): 3319 (NH), 1715 (CO); NMR for major product (**3a**, *E-isomer*) product: ^1^H NMR (500 MHz, DMSO-*d*_*6*_) *δ*_*H*_: 11.89 (s, 0.61 H, N**H**), 8.46–7.14 (m, 16 H, Ar**H**, **H**C **=** N, triazolo -**H**), 5.39 (s, 1.14 H, ArOC**H**_2_); ^13^C NMR (125 MHz, DMSO-*d*_*6*_) *δ*_*C*_: 175.7, 169.2, 168.1, 157.8, 145.8, 19.3, 135.4, 135.1, 134.8, 132.1, 131.7, 130.4, 129.8, 128.7, 127.6, 126.9, 121.8, 118.9, 114.6, 113.2, 66.1; Anal. calcd for C_25_H_19_N_7_O_3_ (465.15), C, 64.51; H, 4.11; N, 21.06; Found: C, 64.28; H, 4.32; N, 21.29.

##### *(E*/*Z*)-2-(2-(3-Benzyl-1,2,4-oxadiazol-5-yl)phenoxy)-*N*’-((2-phenyl-2 H-1,2,3-triazol-4-yl)methylene) acetohydrazide (3b)

White powder (yield 75.0%); m.*p* = 206–207 ˚C; Rf = 0.33 ( n-hexane: EtOAc, 2:1); IR (KBr, cm^− 1^): 3310 (NH), 1709 (CO); NMR for major product (**3b**, *E-isomer*): ^1^H NMR (500 MHz, DMSO-*d*_*6*_) *δ*_*H*_: 11.88 (s, 0.58 H, N**H**), 8.48–7.07 (m, 16 H, Ar**H**, **H**C **=** N, and triazolo -**H**), 5.35 (s, 1.2 H, ArOC**H**_2_), 4.13 (s, 1.18 H, ArC**H**_**2**_); ^13^C NMR (125 MHz, DMSO-*d*_*6*_) *δ*_*C*_: 175.3, 169.7, 169.2, 157.6, 145.8, 139.3, 136.5, 135.1, 134.9, 131.7, 130.4, 129.4, 129.1, 128.6, 127.4, 121.7, 119.0, 114.4, 113.1, 65.9, 31.9; Anal. calcd for C_26_H_21_N_7_O_3_ (479.17), C, 65.13; H, 4.41; N, 20.45; Found: C, 64.90; H, 4.52; N, 20.61.

##### *(E*/*Z*)-*N*’-((2-Phenyl-2 H-1,2,3-triazol-4-yl)methylene)-2-(2-(3-(4-(trifluoromethyl)phenyl)-1,2,4-oxadiazol-5-yl)phenoxy)acetohydrazide (3c)

White powder (yield 72.0), m.*p* = 210–214 ˚C; Rf = 0.27 ( n-hexane: EtOAc, 2:1); IR (KBr, cm^− 1^): 3323 (NH), 1707 (CO); NMR for the major product (**3c**, *E-isomer*): ^1^H NMR (500 MHz, DMSO-*d*_*6*_) *δ*_*H*_: 11.905 (s, 0.61 H, N**H**), 8.46 (s, 0.55 H, triazolo-H), 8.32–7.16 (m, 15 H, Ar**H**, and ArC**H =** N), 5.403 (s, 1.2 H, ArOC**H**_2_); ^13^CNMR (125 MHz, DMSO-*d*_*6*_) *δ*_*C*_: 1761, 169.1, 167.2, 157.9, 145.8, 139.6, 135.3, 135.1, 134.8, 131.9, 131.7, 131.4, 130.7, 130.4, 128.7, 128.5, 126.8, 121.8, 118.9, 114.9, 112.9, 66.1; Anal. calcd for C_26_H_18_F_3_N_7_O_3_ (533.14), C, 58.54; H, 3.40; N, 18.38; Found: C, 58.21; H, 3.32; N, 18.51.

#### General method for the synthesis of 4a-c

To a stirred solution of the acid hydrazide **1a-c** (0.318 mmol) in acetic acid (20 mL) phthalic anhydride (81.9 mg, 0.472 mmol) was added and the reaction mixture was heated near the boiling point for 8 h, then left to cool to room temperature. The solid formed was filtered off to afford the desired product.

##### *N*-(1,3-Dioxoisoindolin-2-yl)-2-(2-(3-phenyl-1,2,4-oxadiazol-5-yl)phenoxy)acetamide (4a)

White solid; (76% yield); m.*p* = 256–259 °C; Rf = 0.66 (n-hexane: EtOAc, 1:1); IR (KBr, cm^− 1^): 3198 (NH), 1739 (CO); ^1^H NMR (500 MHz, DMSO-*d*_*6*_) *δ*_H_: 11.08 (s, 1H, N-**H** ), 8.12–8.10 (m, 1H, Ar-**H**), 7.96–7.93 (m, 6 H, Ar-**H**), 7.73–7.70 ( m, 1H, Ar-**H)**, 7.44–7.23 (m, 5 H, Ar-**H**), 5.14 (s, 2 H, ArOC**H**_**2**_); ^13^C NMR (125 MHz, DMSO-*d*_*6*_) *δ*_*C*_: 174.7, 168.3, 167.8, 165.4, 156.5, 136.1, 135.5, 132.1, 131.4, 129.7, 129.5, 127.5, 126.7, 124.5, 122.7, 115.0, 113.1, 67.3; Anal. calcd for C_24_H_16_N_4_O_5_ (440.11), C, 65.45; H, 3.66; N, 12.72; Found: C, 65.41; H, 3.42; N, 12.81.

##### 2-(2-(3-Benzyl-1,2,4-oxadiazol-5-yl)phenoxy)-*N*-(1,3-dioxoisoindolin-2-yl)acetamide (4b)

White solid (78% yield); m.*p* = 206–209 °C; Rf = 0.32 ( n-hexane: EtOAc, 2:1); IR (KBr, cm^− 1^): 3182 (NH), 1737 (CO); ^1^H NMR (500 MHz, DMSO-*d*_*6*_) *δ*_H_: 11.06 (s, 1H, N-**H** ), 8.04–79.24 (m, 5 H, Ar-**H**), 7.69–7.66 (m, 1H, Ar-**H**), 7.27–7.19 ( m, 2 H, Ar-**H**), 7.075–7.026 (m, 5 H, Ar-**H**), 5.06 (s, 2 H, ArOC**H**_**2**_), 4.07 (s, 2 H, ArC**H**_**2**_); ^13^C NMR (125 MHz, DMSO-*d*_*6*_) *δ*_*C*_:174.3, 169.5, 167.7, 165.2, 156.3, 135.96, 135.92, 15.5, 131.1, 129.9, 128.9, 128.7, 127.22, 124.4, 122.7, 115.0, 112.9, 67.4, 31.7; Anal. calcd for C_25_H_18_N_4_O_5_ (454.13), C, 66.08; H, 3.99; N, 12.33; Found: C, 66.25; H, 4.17; N, 12.14.

##### *N*-(1,3-Dioxoisoindolin-2-yl)-2-(2-(3-(4-(trifluoromethyl)phenyl)-1,2,4-oxadiazol-5-yl)phenoxy)acetamide (4c)

White solid; (70% yield); m.*p* = 210–214 °C; Rf = 0.33 ( n-hexane: EtOAc, 2:1); IR (KBr, cm^− 1^): 3247 (NH), 1738 (CO); ^1^H NMR (500 MHz, DMSO-*d*_*6*_) *δ*_H_:: 11.01 (s, 1H, N-**H** ), 8.18 (d, *J* = 8.5 Hz, 2 H, Ar-**H**), 8.13 (dd, *J* = 7.5 Hz,1.5 Hz, 1H, Ar-**H**), 7.97–7.89 ( m, 4 H, Ar-**H)**, 7.78 (d, *J* = 8.0 Hz, 2 H, Ar-**H**), 7.74–7.66 (m, 1H, Ar-**H**), 7.32 (d, *J* = 8.5 Hz, 2 H, Ar-**H**), 7.30–7.20 (m, 1H, Ar-**H**), 5.13 (s, 2 H, ArOC**H**_**2**_); ^13^C NMR (125 MHz, DMSO-*d*_*6*_) *δ*_*C*_: 175.3, 167.7, 165.3, 156.7, 136.0, 135.7, 131.6, 130.7, 129.7, 128.5, 128.2, 126.6, 126.6, 125.3, 124.4, 122.7, 115.1, 113.1, 67.3; Anal. calcd for C_25_H_15_F_3_N_4_O_5_ (508.10), C, 59.06; H, 2.97; N, 11.02; Found: C, 60.11; H, 2.71; N, 11.19.

#### **General method for the synthesis of the amides 5a, b**

DCC (90 mg, 0.387 mmol) and Et_3_N (65.15 mg, 0.645 mmol) were added to a solution of quinaldic acid (56.43 mg, 0.3225 mmol) and 6-nitro HOBt (69.73 mg, 0.387 mmol) in DMF (2mL) and the solution was stirred for 5 min at 0 °C. The acid hydrazide **1a** or **1b** (0.3225 mmol) was added and the solution was stirred at room temperature for 16 h. The reaction mixture was poured into cold water and the formed solid was filtered. To purify the crude product from the side products, the crude product was refluxed in ethanol (10 mL) for 1 h and filtered while hot, the precipitate was washed with hot ethanol (10 mL), collected, and dried to get the desired peptide.

##### *N*’-(2-(2-(3-Phenyl-1,2,4-oxadiazol-5-yl)phenoxy)acetyl)quinoline-2-carbohydrazide (5a)

White solid (76% yield); m.*p* = 241–243 °C; Rf = 0.3 (n-hexane: EtOAc, 1:1); IR (KBr, cm^− 1^): 3341and 3270 (NH), 1719 and 1666 (CO); ^1^H NMR (500 MHz, DMSO-*d*_*6*_) *δ*_H_: 10.87 (s, 1H, CON-**H** ), 10.15 (s, 1H, CON-**H** ), 8.10 (m, 1H, Ar-**H**), 7.98 (s, 2 H, Ar-**H**), 7.66 ( s, 1H, Ar-**H)**, 7.50–7.44 (d, 5 H, Ar-**H**), 7.29 (s, 2 H, Ar**H** ), 7.20 (s, 2 H, Ar**H** ), 7.02 (s, 1H, Ar**H** ), 6.90 (s, 1H, Ar**H** ), 4.88 (s, 2 H, ArOC**H**_**2**_); ^13^C NMR (125 MHz, DMSO-*d*_*6*_) *δ*_*C*_: 174.6, 170.5, 168.4, 167.1, 156.5, 136.5, 135.6, 135.4, 132.1, 131.3, 129.8, 127.7, 127.5, 126.6, 124.4, 12.4, 121.5, 119.3, 118.9, 14.7, 112.7, 111.8, 108.4, 67.4. Anal. calcd for C_26_H_19_N_5_O_4_ (465.14), C, 67.09; H, 4.11; N, 15.05; Found: C, 67.18; H, 4.25; N, 14.91.

##### *N*’-(2-(2-(3-Benzyl-1,2,4-oxadiazol-5-yl)phenoxy)acetyl)quinoline-2-carbohydrazide (5b)

White solid (74% yield); m.*p* = 206–209 °C; Rf = 0.03 (n-hexane: EtOAc, 1:1); IR (KBr, cm^− 1^): 3355 and 3255 (NH), 1718 and 1667 (CO); ^1^H NMR (500 MHz, DMSO-*d*_*6*_) *δ*_H_: 10.85 (s, 1H, OCN-**H** ), 10.27 (s, 1H, OCN-**H** ), 7.97 (s, 1H, Ar-**H**), 7.57 (s, 2 H, Ar-**H**), 7.21–6.90 ( m, 12 H, Ar-**H)**, 4.82 (s, 2 H, ArOC**H**_**2**_), 3.95 (s, 2 H, ArC**H**_**2**_); ^13^C NMR (125 MHz, DMSO-*d*_*6*_) *δ*_*C*_:174.2, 169.9, 169.91, 166.6, 156.5, 136.6, 136.2, 135.4, 131.1, 129.5, 129.3, 129.1, 127.7, 127.4, 124.4, 122.3, 121.5, 119.3, 118.8, 114.7, 112.8, 111.8, 108.5, 67.4, 31.1. Anal. calcd for C_27_H_21_N_5_O_4_ (479.16), C, 67.63; H, 4.41; N, 14.61; Found: C, 67.51; H, 4.52; N, 14.43.

#### *N*’-(2-(2-(3-(4-(Trifluoromethyl)phenyl)-1,2,4-oxadiazol-5-yl)phenoxy)acetyl)quinoline-2-carbohydrazide (5c)

A mixture of quinaldic acid (22.88 mg, 0.1322 mmol) and SOCl_2_ (5.0 ml) was refluxed for 2 h, then the excess thionyl chloride was evaporated under reduced pressure. Dry THF (5.0 mL), Et_3_N (2.0 mL), and the acid hydrazide **1c** (50 mg, 0.1322 mmol) were added, and the mixture was stirred at room temperature for 16 h. The reaction mixture was poured into cold water and the formed precipitate was filtered. The crude product was recrystallized from ethanol, filtered, and dried to get the desired peptide **5c** (92% yield) as off-white powder, m.*p* = 202–203 °C; Rf = 0.7 (EtOAc : n-hexane, 1:1); IR (KBr, cm^− 1^): 3435 and 3321 (NH), 1718 and 1683 (CO); ^1^H NMR (500 MHz, DMSO-*d*_*6*_) *δ*_H_: 10.96 (s, 1H, OCN-**H**), 10.45 (s, 1H, OCN-**H**), 8.55 (d, *J* = 8.5 Hz, 1H, Ar-**H**), 8.19–8.13 (m, 4 H, Ar-**H**), 8.09–8.03 (m, 2 H, Ar-**H**), 7.88 (t, *J* = 7.0 Hz, 1H, Ar-**H**), 7.729 (t, *J* = 7.0 Hz, 2 H, Ar**H** ), 7.61 (d, *J* = 8.5 Hz, 2 H, Ar**H** ), 7.35 (d, *J* = 8.5 Hz, 1H, Ar**H**), 7.25 (t, *J* = 8.0 Hz, 1H, Ar**H** ), 5.00 (s, 2 H, ArOC**H**_**2**_); ^13^C NMR (125 MHz, DMSO-*d*_*6*_) *δ*_*C*_: 174.9, 167.5, 167.2, 163.8, 156.4, 149.4, 146.7, 138.6, 135.9, 131.2, 131.2, 130.5, 129.7, 129.5, 128.9, 128.7, 128.5, 126.5, 126.47, 126.44, 122.6, 119.2, 114.9, 112.4, 67.7. Anal. calcd for C_27_H_18_F_3_N_5_O_4_ (533.13), C, 60.79; H, 3.40; N, 13.13; Found: C, 60.56; H, 3.53; N, 13.24.

### Biological evaluation

The biological experiments were conducted in compliance with the previously documented protocols and are available in the **Supplementary Materials**; **AChE and BuChE inhibitory assays** [61], **DPPH radical scavenging activity** [62], **MAO-B and MAO-A inhibitory assays** [63], and **the anti-hemolytic effect of synthesized compounds** [64].

### Molecular modeling studies

The x-ray structure of the of AChE (PDB: 7E3H) was downloaded from the protein databank (PDB) website, (https://www.rcsb.org/) at a resolution of 1.90 Å and 2.00 Å respectively. All the molecular modeling and docking studies were carried out using MOE 2020.09 (Chemical Computing Group, Canada) as the computational software. First, all the hydrogen atoms were added using the Protonate 3D algorithm where the protonation states of the amino acid residues were assigned, and the partial charges of atoms were added. In addition, the compounds were drawn using the builder tool and energy was minimized using the MMFF94x force field. MOE induced-fit Dock tool used to dock the synthesized compounds into the active site. The selection of the final docked ligand–enzyme poses was according to the criteria of binding energy score and combined with ligand-receptor interactions.

### Electronic supplementary material

Below is the link to the electronic supplementary material.

Supplementary Material 1

## Data Availability

This published article and its additional file includes some data generated or analyzed during this study. Other datasets used and analyzed during the current study are available from the corresponding author on reasonable request.

## Declarations

We obtained informed consent from all of the participants in the study, they are three individual participants from our research teamwork. Also, we obtained ethics approval from Faculty of science, Alexandria University. Medical Research Ethics Committee (Approval number AU04 608 24 01 2024).

## References

[1] Du X, Wang X, Geng M (2018). Alzheimer’s disease hypothesis and related therapies. Transl Neurodegener.

[2] Matej R, Tesar A, Rusina R (2019). Alzheimer’s disease and other neurodegenerative dementias in comorbidity: a clinical and neuropathological overview. Clin Biochem.

[3] Karantzoulis S, Galvin JE (2011). Distinguishing Alzheimer’s disease from other major forms of dementia. Expert Rev Neurother.

[4] Buckner RL (2004). Memory and executive function in aging and AD. Neuron.

[5] Butcher HK, Holkup PA, Buckwalter KC (2001). The experience of caring for a Family Member with Alzheimer’s Disease. West J Nurs Res.

[6] Feigin V. GBD 2015 Mortality and Causes of Death Collaborators. Global, regional, and national life expectancy, all-cause mortality, and cause-specific mortality for 249 causes of death, 1980–2015: a systematic analysis for the Global Burden of Disease Study 2015, Lancet. 388 (2016) 1459–1544. 10.1016/S0140-6736(16)31012-1 PMC5388903 27733281

[7] Association A. 2017 Alzheimer’s disease facts and figures, Alzheimer’s Dement. 13 (2017) 325–373. 10.1016/j.jalz.2017.02.001.

[8] Nandi A, Counts N, Chen S, Seligman B, Tortorice D, Vigo D (2022). Global and regional projections of the economic burden of Alzheimer’s disease and related dementias from 2019 to 2050: a value of statistical life approach. EClinicalMedicine.

[9] Bonin-Guillaume S, Zekry D, Giacobini E, Gold G, Michel J-P (2005). Impact économique de la démence. Presse Med.

[10] Hampel H, Mesulam M-M, Cuello AC, Khachaturian AS, Vergallo A, Farlow MR, REVISITING THE CHOLINERGIC HYPOTHESIS IN ALZHEIMER’S DISEASE (2018). EMERGING EVIDENCE FROM TRANSLATIONAL AND CLINICAL RESEARCH. J Prev Alzheimer’s Dis.

[11] Arnsten AFT, Datta D, Del Tredici K, Braak H (2021). Hypothesis: tau pathology is an initiating factor in sporadic Alzheimer’s disease. Alzheimer’s Dement.

[12] Liu P-P, Xie Y, Meng X-Y, Kang J-S (2019). History and progress of hypotheses and clinical trials for Alzheimer’s disease, Signal Transduct. Target Ther.

[13] Cai Z, Zhao B, Ratka A (2011). Oxidative stress and β-Amyloid protein in Alzheimer’s Disease. NeuroMolecular Med.

[14] Wessler I, Kirkpatrick CJ (2008). Acetylcholine beyond neurons: the non-neuronal cholinergic system in humans. Br J Pharmacol.

[15] Coulson FR, Fryer AD (2003). Muscarinic acetylcholine receptors and airway diseases. Pharmacol Ther.

[16] Prakriya M, Miller RJ (2008). Neuronal and glial signaling. Neuroimmune Pharmacol.

[17] Pohanka M (2011). Cholinesterases, a target of pharmacology and toxicology. Biomed Pap.

[18] Inestrosa NC, Sagal JP, Colombres M. Acetylcholinesterase Interaction with Alzheimer amyloid β. Alzheimer’s Dis. Springer US; 2005. pp. 299–317. 10.1007/0-387-23226-5_15. 10.1007/0-387-23226-5_15 15709485

[19] Inestrosa NC, Dinamarca MC, Alvarez A (2008). Amyloid–cholinesterase interactions. FEBS J.

[20] Kumar A, Pintus F, Di Petrillo A, Medda R, Caria P, Matos MJ (2018). Novel 2-pheynlbenzofuran derivatives as selective butyrylcholinesterase inhibitors for Alzheimer’s disease. Sci Rep.

[21] Nordberg A, Ballard C, Bullock R, Darreh-Shori T, Somogyi M (2013). A review of butyrylcholinesterase as a therapeutic target in the treatment of Alzheimer’s Disease, Prim. Care Companion CNS Disord.

[22] Bartolini M, Bertucci C, Cavrini V, Andrisano V (2003). β-Amyloid aggregation induced by human acetylcholinesterase: inhibition studies. Biochem Pharmacol.

[23] Camps P, Formosa X, Galdeano C, Gómez T, Muñoz-Torrero D, Scarpellini M (2008). Novel donepezil-based inhibitors of Acetyl- and Butyrylcholinesterase and Acetylcholinesterase-Induced β-Amyloid aggregation. J Med Chem.

[24] Zuin M, Cherubini A, Volpato S, Ferrucci L, Zuliani G (2022). Acetyl-cholinesterase-inhibitors slow cognitive decline and decrease overall mortality in older patients with dementia. Sci Rep.

[25] Wojtunik-Kulesza KA, Oniszczuk A, Oniszczuk T, Waksmundzka-Hajnos M (2016). The influence of common free radicals and antioxidants on development of Alzheimer’s Disease. Biomed Pharmacother.

[26] Di Domenico F, Barone E, Perluigi M, Butterfield DA (2015). Strategy to reduce free radical species in Alzheimer’s disease: an update of selected antioxidants. Expert Rev Neurother.

[27] Binda C, Milczek EM, Bonivento D, Wang J, Mattevi A, Edmondson DE (2011). Lights and shadows on Monoamine Oxidase Inhibition in neuroprotective pharmacological therapies. Curr Top Med Chem.

[28] Finberg JPM (2014). Update on the pharmacology of selective inhibitors of MAO-A and MAO-B: focus on modulation of CNS monoamine neurotransmitter release. Pharmacol Ther.

[29] Chan HH, Tse MK, Kumar S, Zhuo L (2018). A novel selective MAO-B inhibitor with neuroprotective and anti-parkinsonian properties. Eur J Pharmacol.

[30] Tabakman R, Lecht S, Lazarovici P (2004). Neuroprotection by monoamine oxidase B inhibitors: a therapeutic strategy for Parkinson’s disease?. BioEssays.

[31] Naoi M, Maruyama W (2010). Monoamine Oxidase inhibitors as neuroprotective agents in Age-Dependent Neurodegenerative disorders. Curr Pharm Des.

[32] Youdim MBH, Edmondson D, Tipton KF (2006). The therapeutic potential of monoamine oxidase inhibitors. Nat Rev Neurosci.

[33] Finberg JPM, Rabey JM (2016). Inhibitors of MAO-A and MAO-B in Psychiatry and Neurology. Front Pharmacol.

[34] Nicotra A (2004). Monoamine Oxidase expression during Development and Aging. Neurotoxicology.

[35] Kumar MJ, Andersen JK (2004). Perspectives on MAO-B in aging and neurological disease: where do we go from Here?. Mol Neurobiol.

[36] Katsouri L, Vizcaychipi MP, McArthur S, Harrison I, Suárez-Calvet M, Lleo A (2013). Prazosin, an α1-adrenoceptor antagonist, prevents memory deterioration in the APP23 transgenic mouse model of Alzheimer’s disease. Neurobiol Aging.

[37] Mallajosyula JK, Kaur D, Chinta SJ, Rajagopalan S, Rane A, Nicholls DG (2008). MAO-B elevation in mouse brain astrocytes results in Parkinson’s Pathology. PLoS ONE.

[38] Baker G, Matveychuk D, MacKenzie EM, Holt A, Wang Y, Kar S (2019). Attenuation of the effects of oxidative stress by the MAO-inhibiting antidepressant and carbonyl scavenger phenelzine. Chem Biol Interact.

[39] Bortolato M, Chen K, Shih JC (2008). Monoamine oxidase inactivation: from pathophysiology to therapeutics. Adv Drug Deliv Rev.

[40] Weinreb O, Amit T, Bar-Am O, Youdim MBH. A novel anti-Alzheimer’s disease drug, ladostigil, in: Int. Rev. Neurobiol., Elsevier, 2011: pp. 191–215. 10.1016/B978-0-12-386467-3.00010-8. 10.1016/B978-0-12-386467-3.00010-8 21971009

[41] Zheng H, Gal S, Weiner LM, Bar-Am O, Warshawsky A, Fridkin M (2005). Novel multifunctional neuroprotective iron chelator‐monoamine oxidase inhibitor drugs for neurodegenerative diseases: in vitro studies on antioxidant activity, prevention of lipid peroxide formation and monoamine oxidase inhibition. J Neurochem.

[42] Park J-H, Ju YH, Choi JW, Song HJ, Jang BK, Woo J (2019). Newly developed reversible MAO-B inhibitor circumvents the shortcomings of irreversible inhibitors in Alzheimer’s disease. Sci Adv.

[43] Carradori S, Silvestri R, Inhibitors (2015). J Med Chem.

[44] Iqbal K, Grundke-Iqbal I (2010). Alzheimer’s disease, a multifactorial disorder seeking multitherapies. Alzheimer’s Dement.

[45] Manzoor S, Prajapati SK, Majumdar S, Raza MK, Gabr MT, Kumar S (2021). Discovery of new phenyl sulfonyl-pyrimidine carboxylate derivatives as the potential multi-target drugs with effective anti-alzheimer’s action: design, synthesis, crystal structure and in-vitro biological evaluation. Eur J Med Chem.

[46] Umar T, Shalini S, Raza MK, Gusain S, Kumar J, Seth P (2019). A multifunctional therapeutic approach: synthesis, biological evaluation, crystal structure and molecular docking of diversified 1H-pyrazolo[3,4-b]pyridine derivatives against Alzheimer’s disease. Eur J Med Chem.

[47] Umar T, Gusain S, Raza MK, Shalini S, Kumar J, Tiwari M (2019). Naphthalene-Triazolopyrimidine hybrid compounds as potential multifunctional anti-alzheimer’s agents. Bioorg Med Chem.

[48] Camci M, Karali N (2023). Bioisosterism: 1,2,4-Oxadiazole rings. ChemMedChem.

[49] Biernacki K, Daśko M, Ciupak O, Kubiński K, Rachon J, Demkowicz S (2020). Novel 1,2,4-Oxadiazole derivatives in Drug Discovery. Pharmaceuticals.

[50] Ono M, Haratake M, Saji H, Nakayama M (2008). Development of novel β-amyloid probes based on 3,5-diphenyl-1,2,4-oxadiazole. Bioorg Med Chem.

[51] Wang M, Liu T, Chen S, Wu M, Han J, Li Z (2021). Design and synthesis of 3-(4-pyridyl)-5-(4-sulfamido-phenyl)-1,2,4-oxadiazole derivatives as novel GSK-3β inhibitors and evaluation of their potential as multifunctional anti-alzheimer agents. Eur J Med Chem.

[52] Wang Y, Xiong B, Lin H, Li Q, Yang H, Qiao Y, et al. Design, synthesis and evaluation of fused hybrids with acetylcholinesterase inhibiting and Nrf2 activating functions for Alzheimer’s disease. Eur J Med Chem. 2022;244. 10.1016/j.ejmech.2022.114806. 10.1016/j.ejmech.2022.114806 36223681

[53] Liu T, Chen S, Du J, Xing S, Li R, Li Z (2022). Design, synthesis, and biological evaluation of novel (4-(1,2,4-oxadiazol-5-yl)phenyl)-2-aminoacetamide derivatives as multifunctional agents for the treatment of Alzheimer’s disease. Eur J Med Chem.

[54] Mohammadi-Khanaposhtani M, Mahdavi M, Saeedi M, Sabourian R, Safavi M, Khanavi M (2015). Design, synthesis, Biological evaluation, and Docking Study of acetylcholinesterase inhibitors: New Acridone‐1,2,4‐oxadiazole‐1,2,3‐triazole hybrids. Chem Biol Drug Des.

[55] Rullo M, La Spada G, Miniero DV, Gottinger A, Catto M, Delre P (2023). Bioisosteric replacement based on 1,2,4-oxadiazoles in the discovery of 1H-indazole-bearing neuroprotective MAO B inhibitors. Eur J Med Chem.

[56] Ayoup MS, Abu-Serie MM, Abdel-Hamid H, Teleb M (2021). Beyond direct Nrf2 activation; reinvestigating 1,2,4-oxadiazole scaffold as a master key unlocking the antioxidant cellular machinery for cancer therapy. Eur J Med Chem.

[57] Ayoup MS, ElShafey MM, Abdel-Hamid H, Ghareeb DA, Abu-Serie MM, Heikal LA (2023). Repurposing 1,2,4-oxadiazoles as SARS-CoV-2 PLpro inhibitors and investigation of their possible viral entry blockade potential. Eur J Med Chem.

[58] 2020.09 Chemical Computing Group ULC, 1010 Sherbooke St. West, Suite #910, Montreal, QC, Molecular Operating Environment (MOE), Canada. H3A 2R7, (2022).

[59] Dileep KV, Ihara K, Mishima-Tsumagari C, Kukimoto-Niino M, Yonemochi M, Hanada K (2022). Crystal structure of human acetylcholinesterase in complex with tacrine: implications for drug discovery. Int J Biol Macromol.

[60] Lipinski CA (2004). Lead- and drug-like compounds: the rule-of-five revolution. Drug Discov Today Technol.

[61] Mukherjee PK, Kumar V, Houghton PJ (2007). Screening of Indian medicinal plants for acetylcholinesterase inhibitory activity. Phyther Res.

[62] Braca A, De Tommasi N, Di Bari L, Pizza C, Politi M, Morelli I (2001). Antioxidant principles from Bauhinia t arapotensis. J Nat Prod.

[63] Holt A, Palcic MM (2006). A peroxidase-coupled continuous absorbance plate-reader assay for flavin monoamine oxidases, copper-containing amine oxidases and related enzymes. Nat Protoc.

[64] Tantary S, Masood A, Bhat AH, Dar KB, Zargar MA, Ganie SA (2016). In vitro antioxidant and RBC membrane stabilization activity of Euphorbia wallichii. Free Radicals Antioxid.

