# Supplementary material for: New 1,2,4-oxadiazole derivatives as potential multifunctional agents for the treatment of Alzheimer’s disease: design, synthesis, and biological evaluation
Source: BMC Chem. 2024 Jul 13;18(1):130. doi: 10.1186/s13065-024-01235-x (PMC11246588; doi:10.1186/s13065-024-01235-x)
Supplement: Supplementary file 1 — Supplementary Material 1 [file 13065_2024_1235_MOESM1_ESM.pdf]

Supplementary Information

New 1,2,4-oxadiazole derivatives as potential multifunctional agents for the treatment of Alzheimer's disease: design, synthesis, and biological evaluation

Mohammed Salah Ayoup [a, b] *, Mariam Ghanem [b], Hamida Abdel-Hamid [b], Marwa M. Abu-Serie [c], Aliaa Masoud [d], Doaa A. Ghareeb [d,e,f], Mohammed B. Hawsawi [g], Amr Sonousi [h,i], Asmaa E. Kassab [h] *

[a] *Department of Chemistry, College of Science, King Faisal University, Al-Ahsa 31982, Saudi Arabia*
[b] *Chemistry Department, Faculty of Science, Alexandria University, P.O. Box 426, Alexandria, 21321, Egypt*
[c] *Medical Biotechnology Department, Genetic Engineering and Biotechnology Research Institute, City of Scientific Research and Technological Applications (SRTA-City), Egypt.*
[d] *Bio-screening and Preclinical Trial Lab, Biochemistry Department, Faculty of Science, Alexandria University, 21511, Alexandria, Egypt*
[e] *Center of Excellence for Drug Preclinical Studies (CE-DPS), Pharmaceutical and Fermentation Industry Development Center, City of Scientific Research & Technological Applications (SRTA-city), New Borg El Arab, Alexandria, Egypt*
[f] *Research Projects Unit, Pharos University in Alexandria, Egypt*
[g] *Department of Chemistry, Faculty of Science, Umm Al-Qura University, Makkah 21955, Saudi Arabia*
[h] *Department of Pharmaceutical Organic Chemistry, Faculty of Pharmacy, Cairo University, Kars El-Aini Street, Cairo, P.O. Box 11562, Egypt.*
[i] *University of Hertfordshire hosted by Global Academic Foundation, New Administrative Capital, Cairo, Egypt*

* Corresponding author at *Department of Chemistry, College of Science, King Faisal University, Al-Ahsa 31982, Saudi Arabia.*
Chemistry Department, Faculty of Science, Alexandria University, P.O. Box 426, Alexandria, 21321, Egypt.
Department of Pharmaceutical Organic Chemistry, Faculty of Pharmacy, Cairo University, Kasr El-Aini Street, Cairo, P.O. Box 11562, Egypt.
Email addresses: mayoup@kfu.edu.sa, mohammedsalahayoup@gmail.com, asmaa.kassab@pharma.cu.edu.eg

Contents

1	^{1}H and ^{13}C-NMR of compounds **1a-c** to **5a-c**	**S2-S25**
2	Equipment and analytical technique	**S26**
3	Biological procedures	**S26-S27**
4	Molecular modelling and ADME study	**S29-S36**
5	Physicochemical properties and ADME properties of the target compounds	**S37**
6	References	**S38**

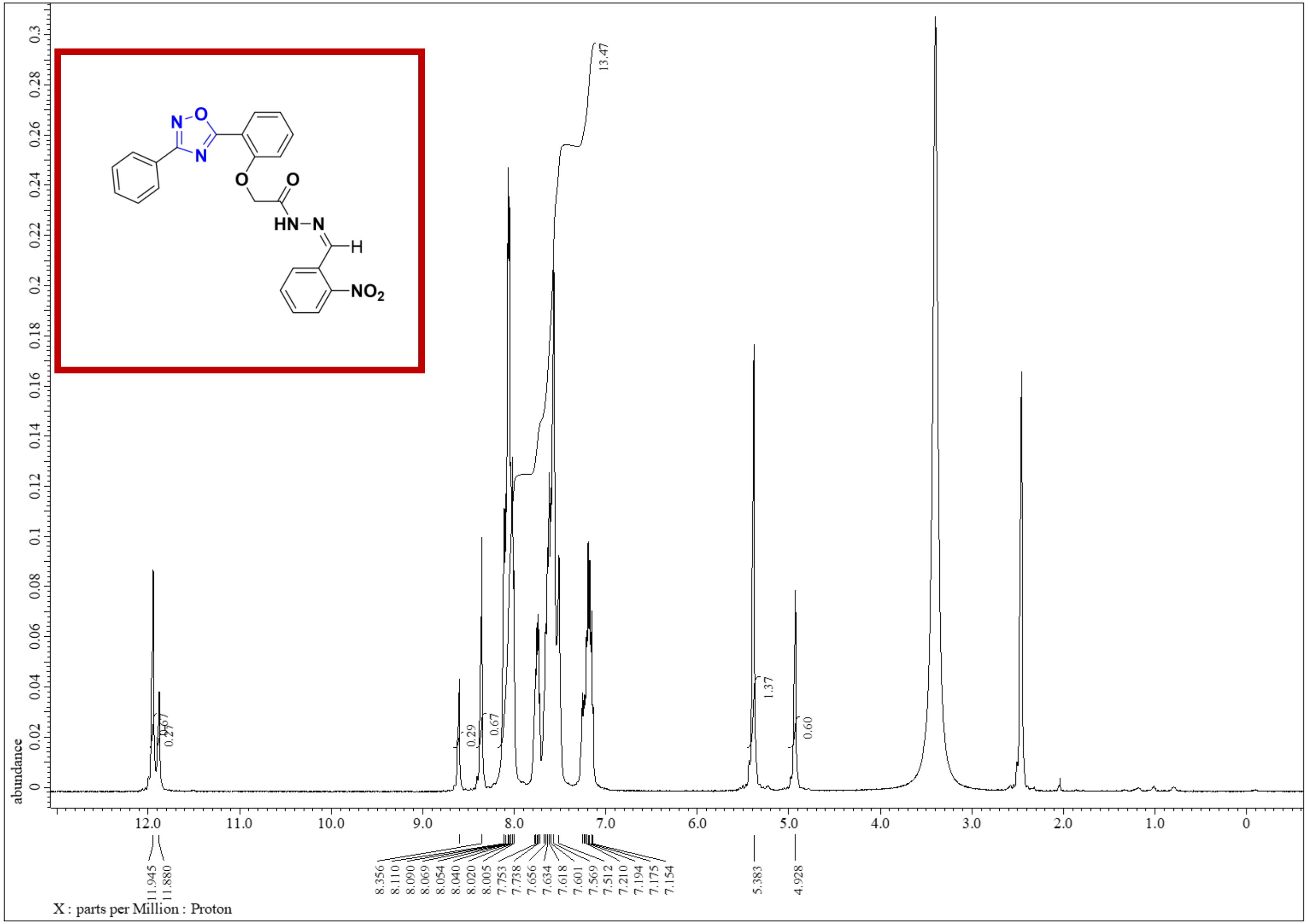

Fig. S1 ^{1}H-NMR spectrum (500 MHz, DMSO-d$_6$) of **2a.**

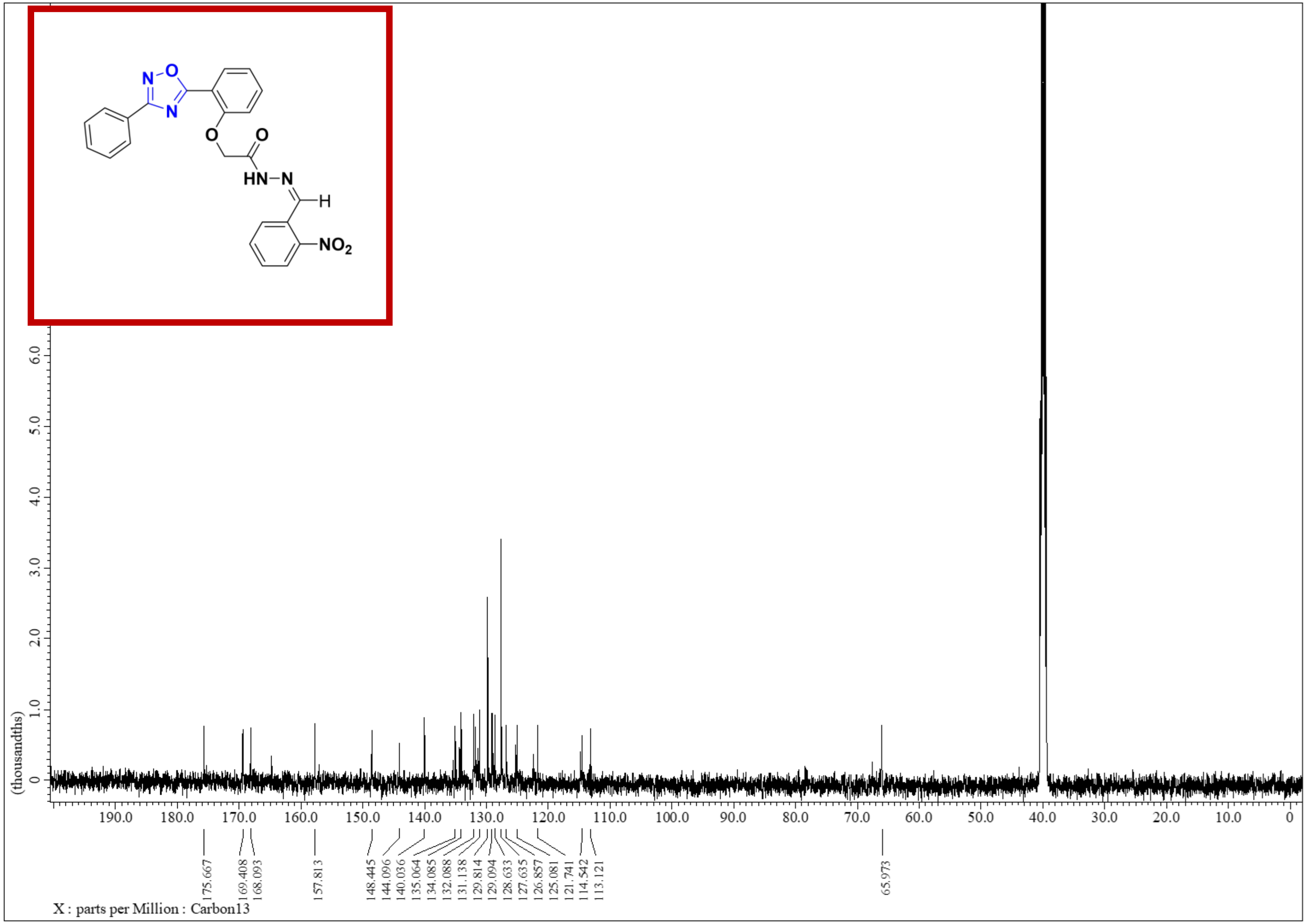

Fig. S2 ^{13}C-NMR spectrum (125 MHz, DMSO-d_6) of **2a.**

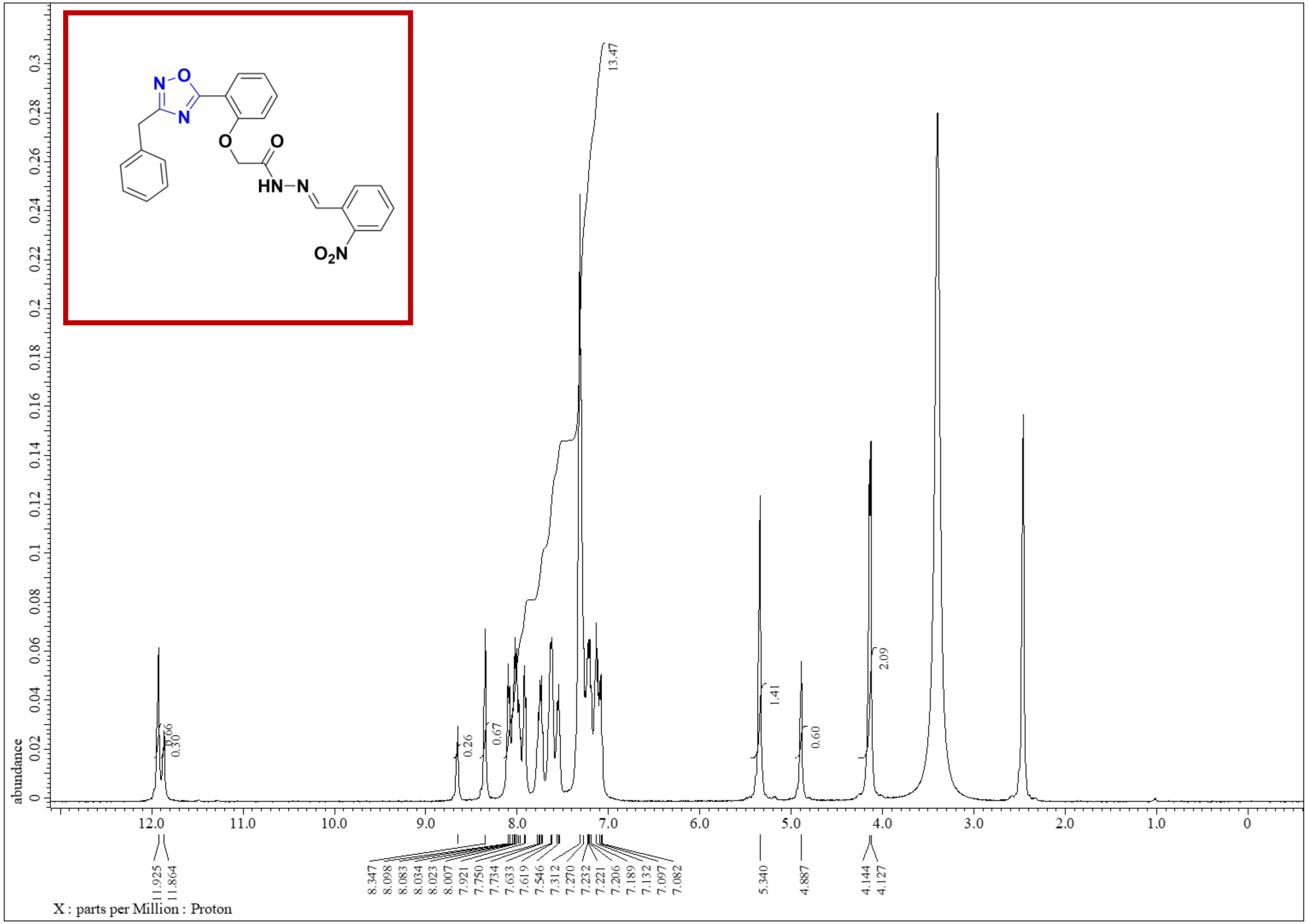

Fig. S3 ^{1}H-NMR spectrum (500 MHz, DMSO-d_6) of **2b.**

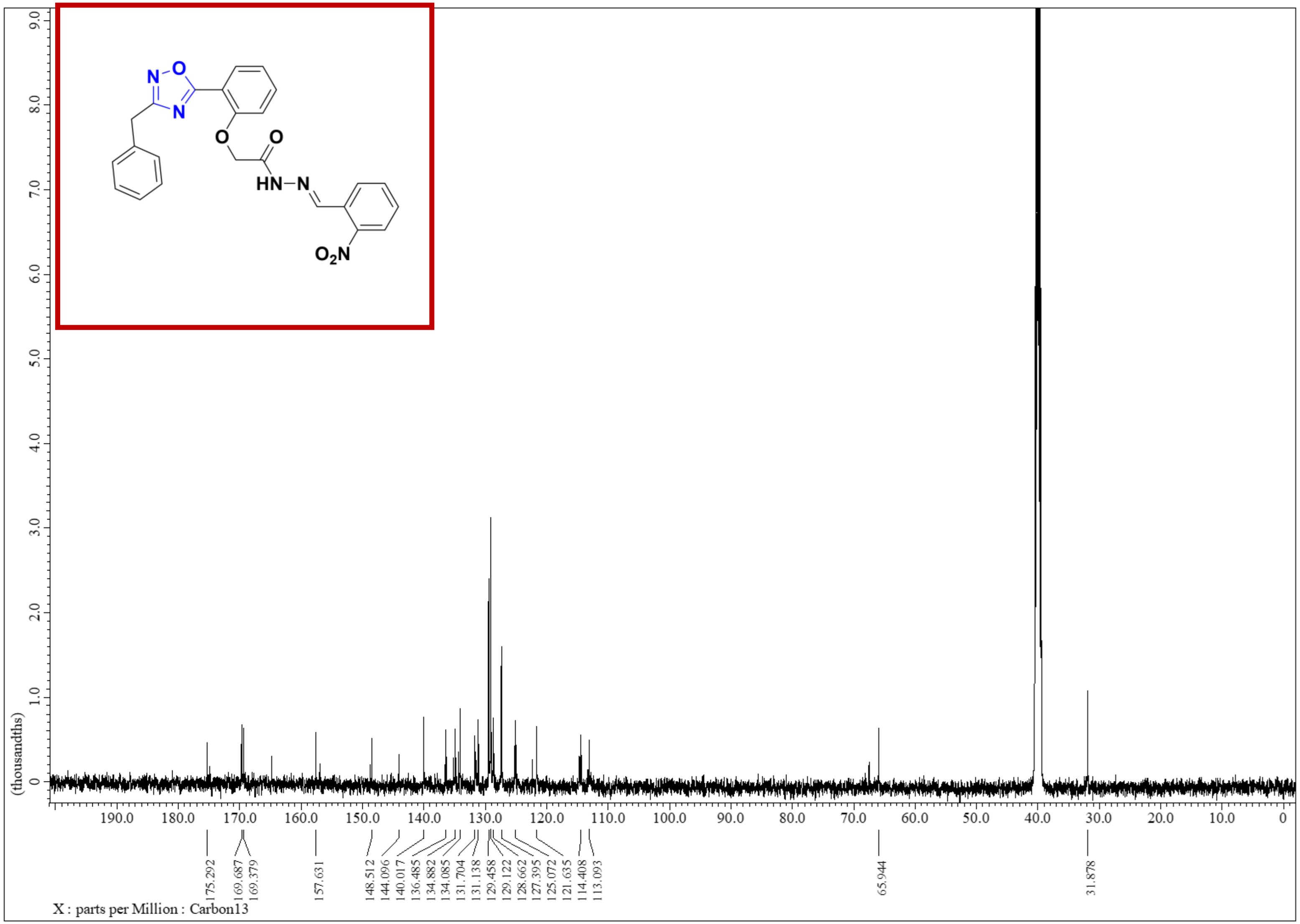

Fig. S4 ^{13}C-NMR spectrum (125 MHz, DMSO-d$_6$) of **2b.**

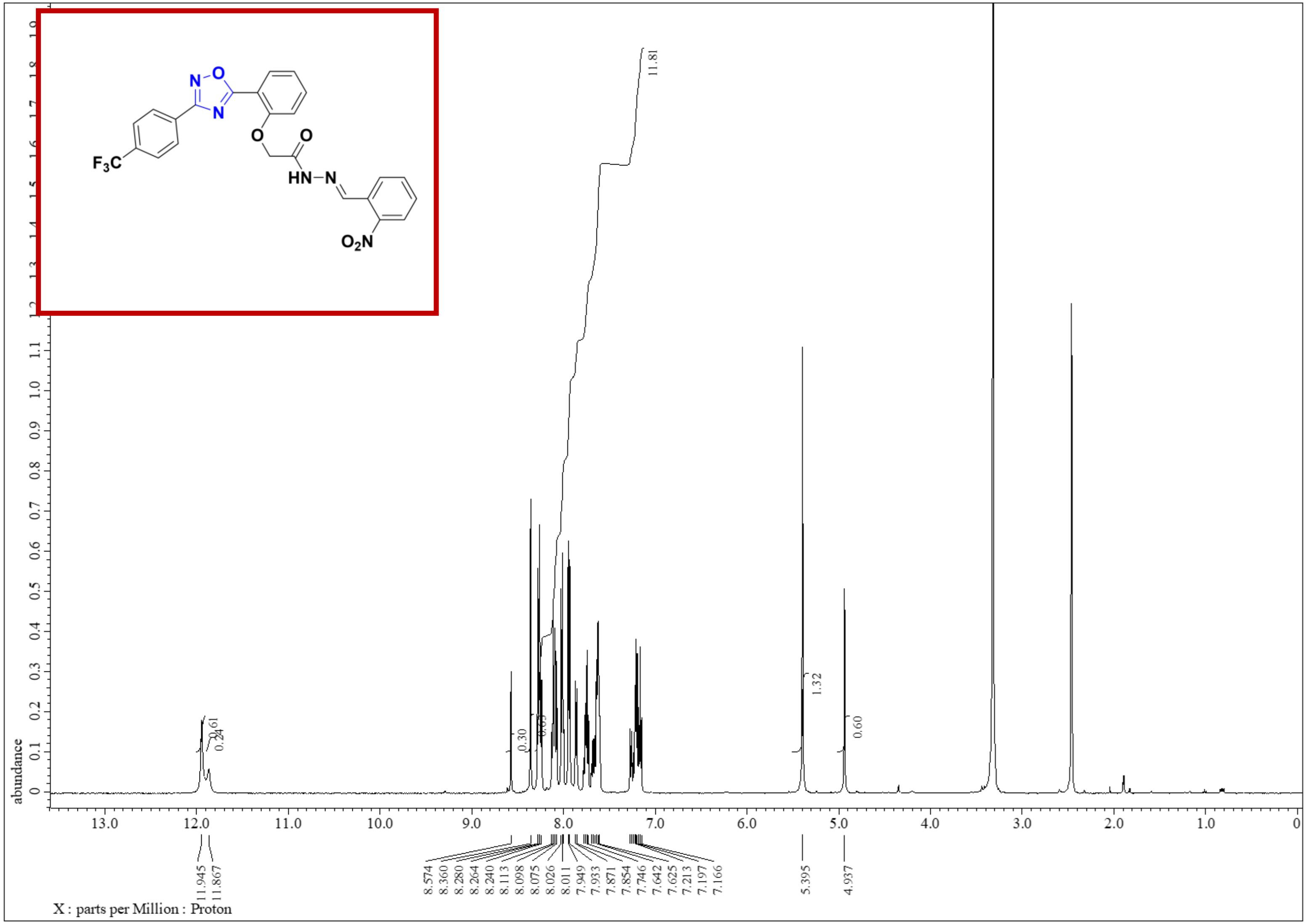

Fig. S5 ^{1}H-NMR spectrum (500 MHz, DMSO-d_6) of **2c.**

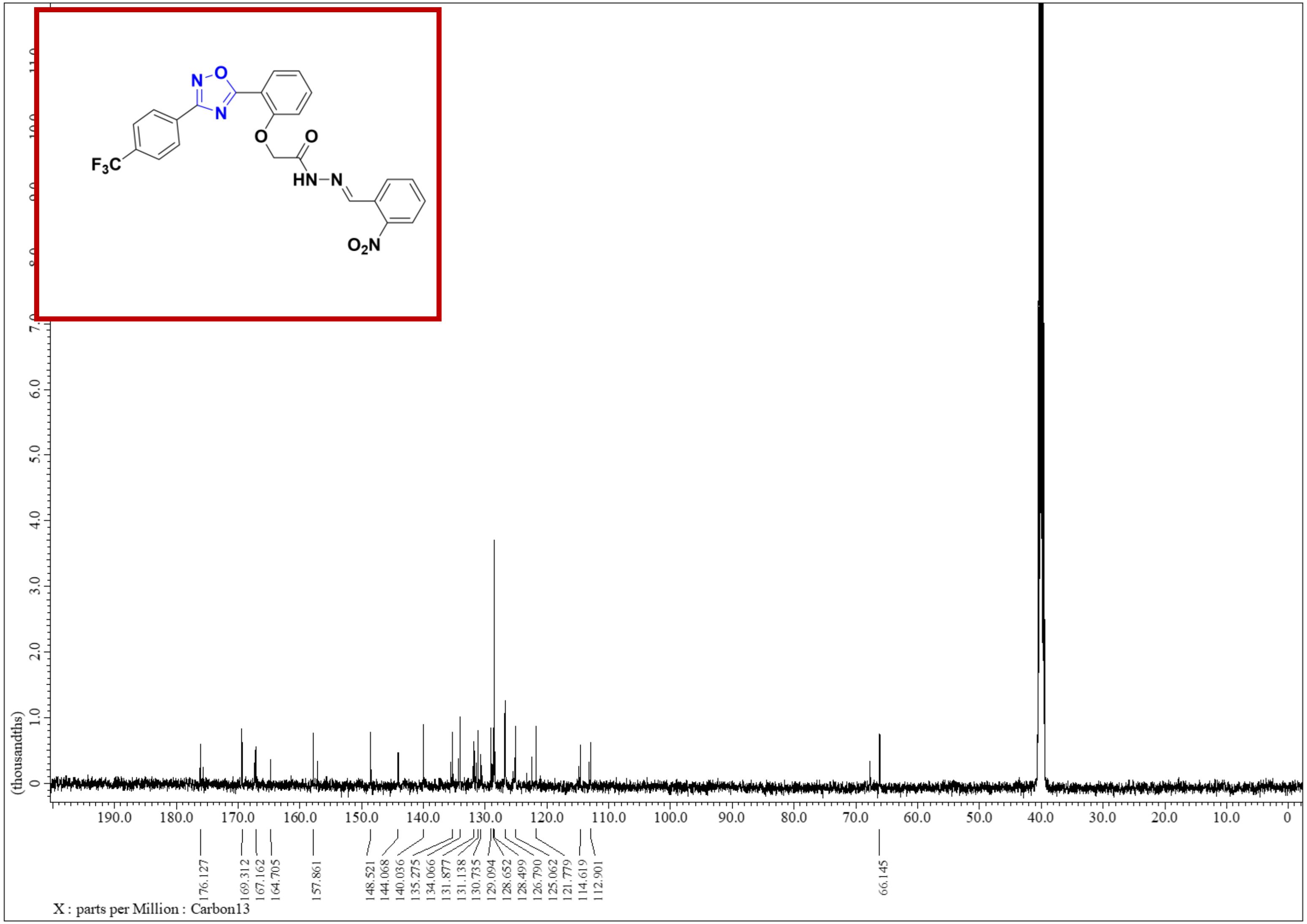

Fig. S6 ^{13}C-NMR spectrum (125 MHz, DMSO-d_6) of **2c.**

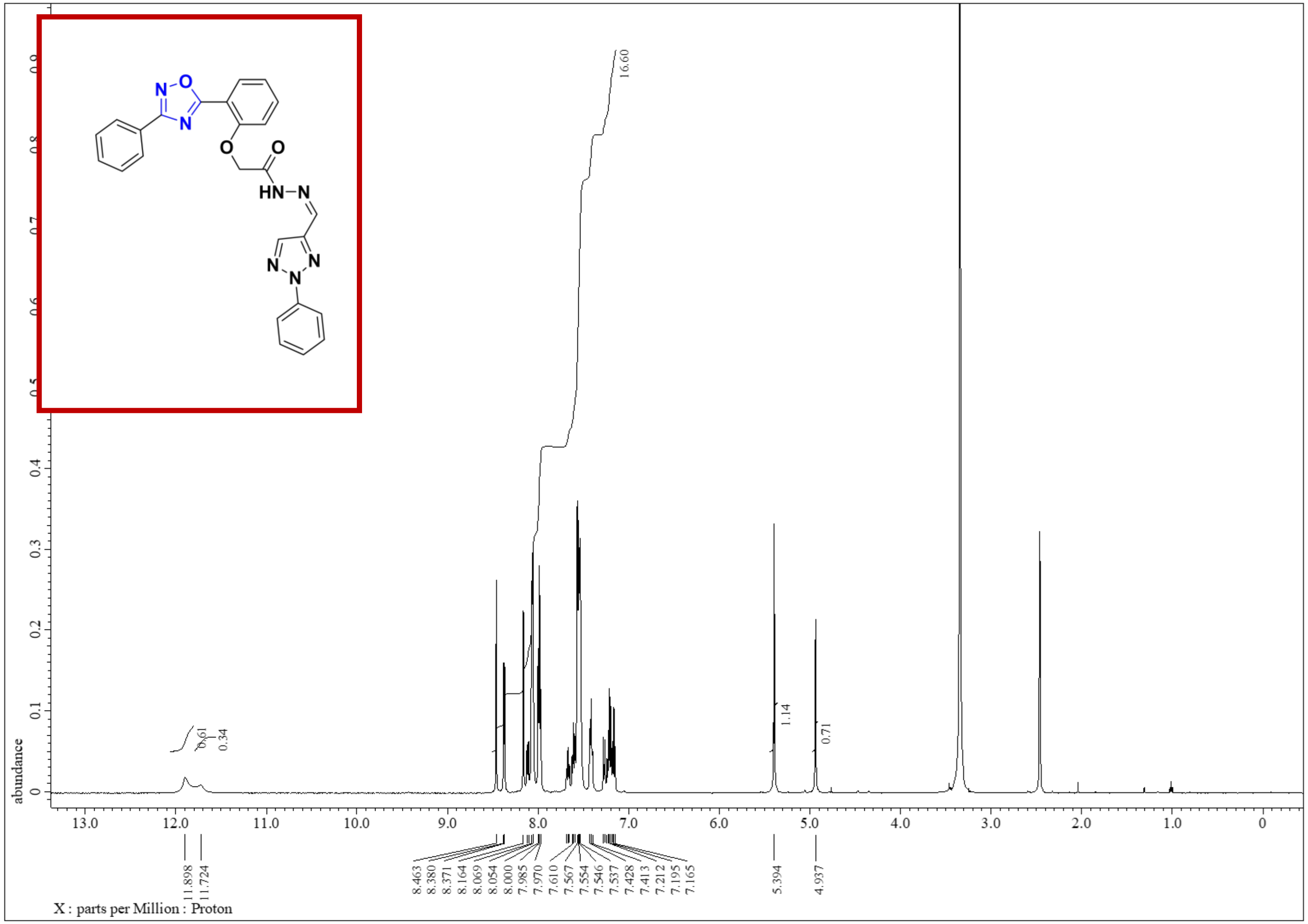

Fig. S7 ^{1}H-NMR spectrum (500 MHz, DMSO-d_6) of **3a.**

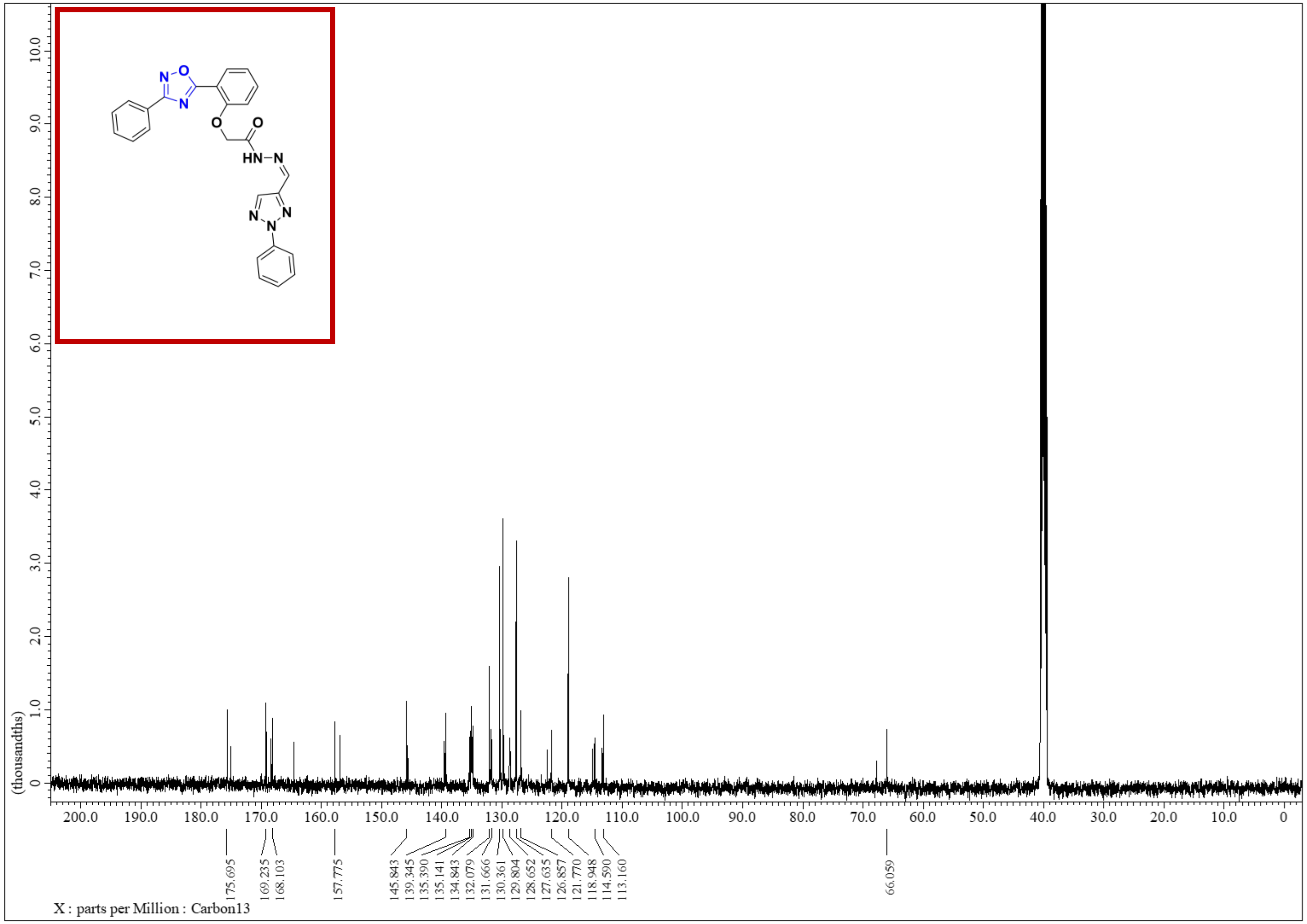

Fig. S8 ^{13}C-NMR spectrum (125 MHz, DMSO-d_6) of **3a.**

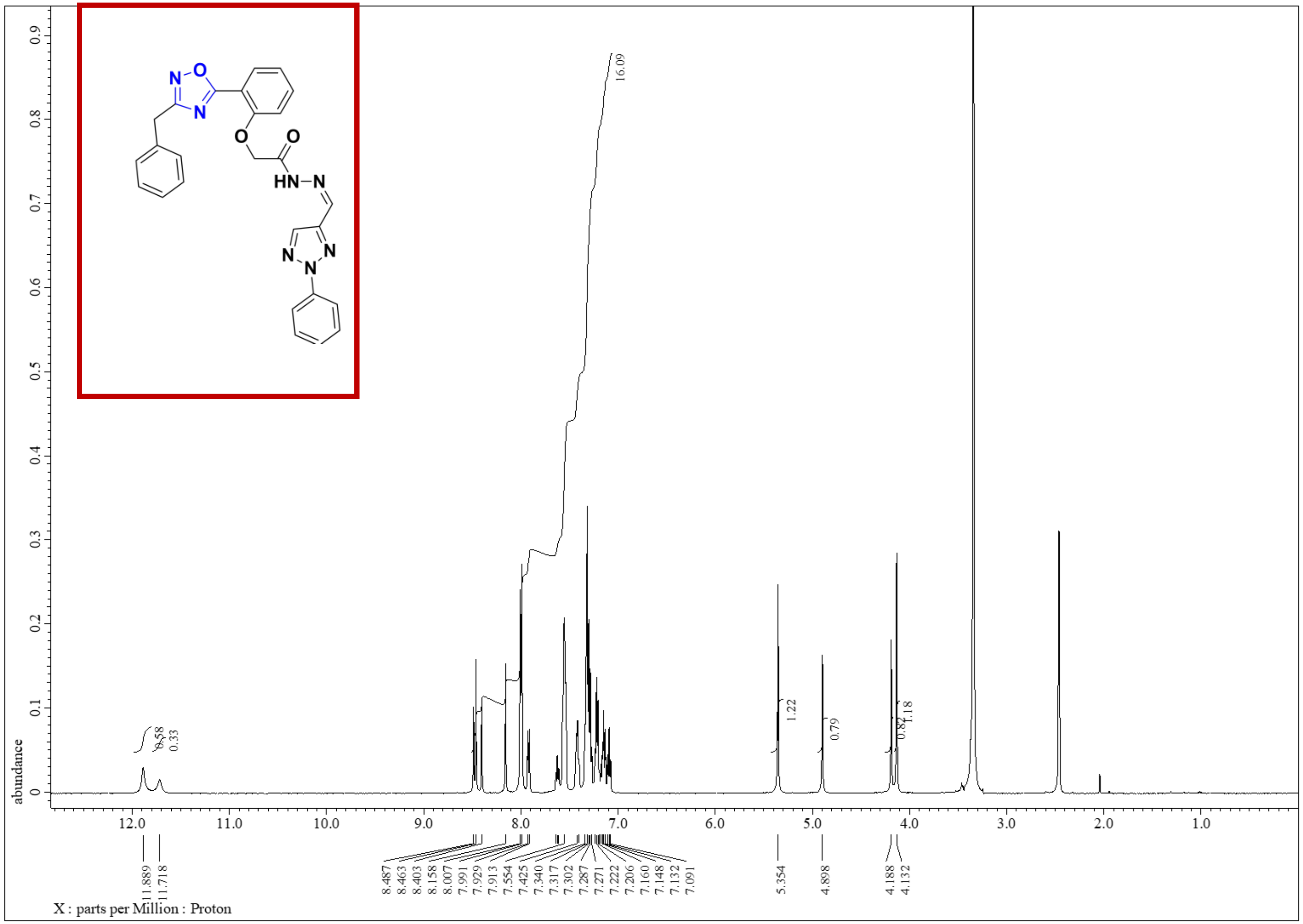

Fig. S9 ^{1}H-NMR spectrum (500 MHz, DMSO-d$_6$) of **3b.**

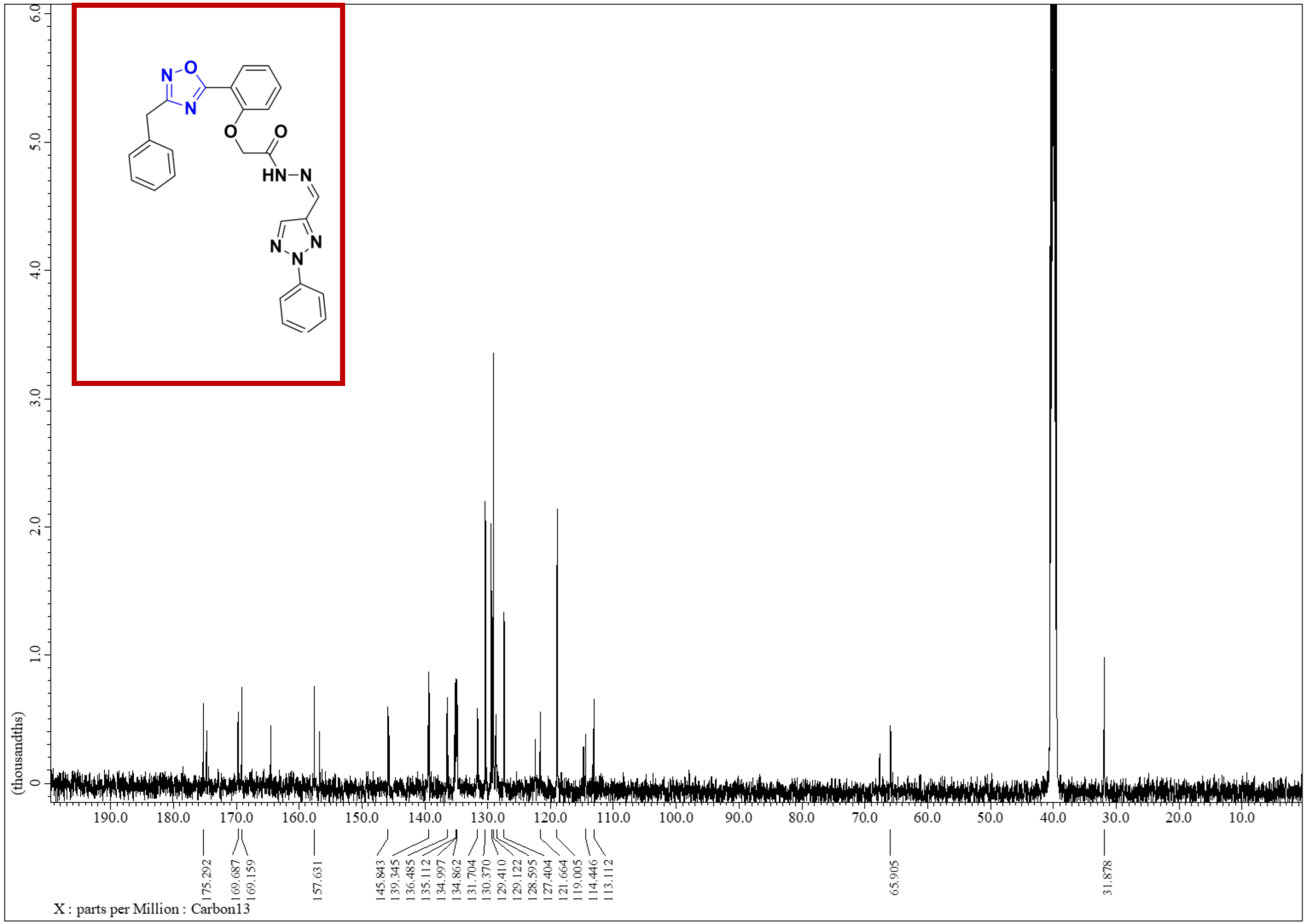

Fig. S10 ^{13}C-NMR spectrum (125 MHz, DMSO-d$_6$) of **3b.**

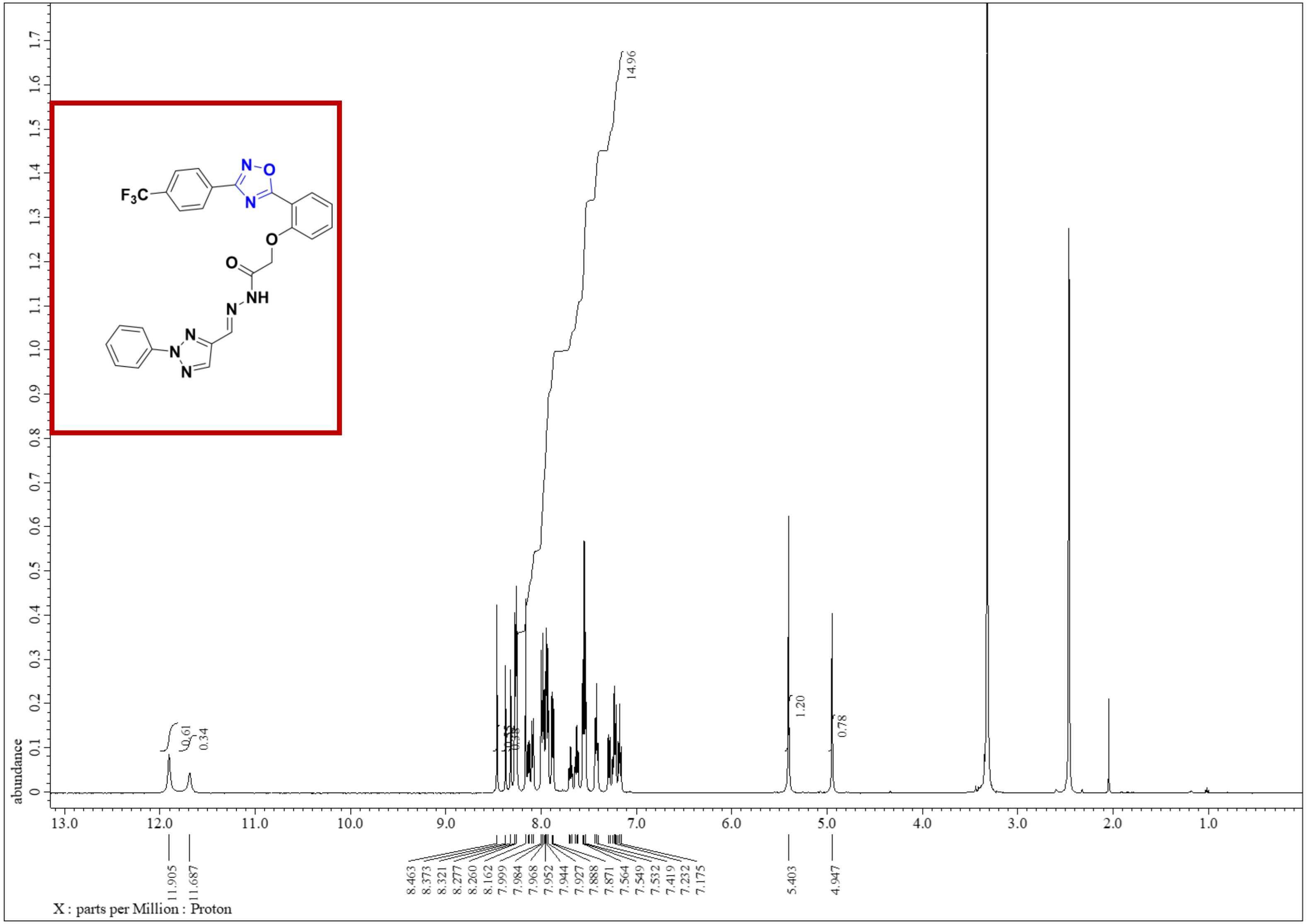

Fig. S11 ^{1}H-NMR spectrum (500 MHz, DMSO-d_6) of **3c.**

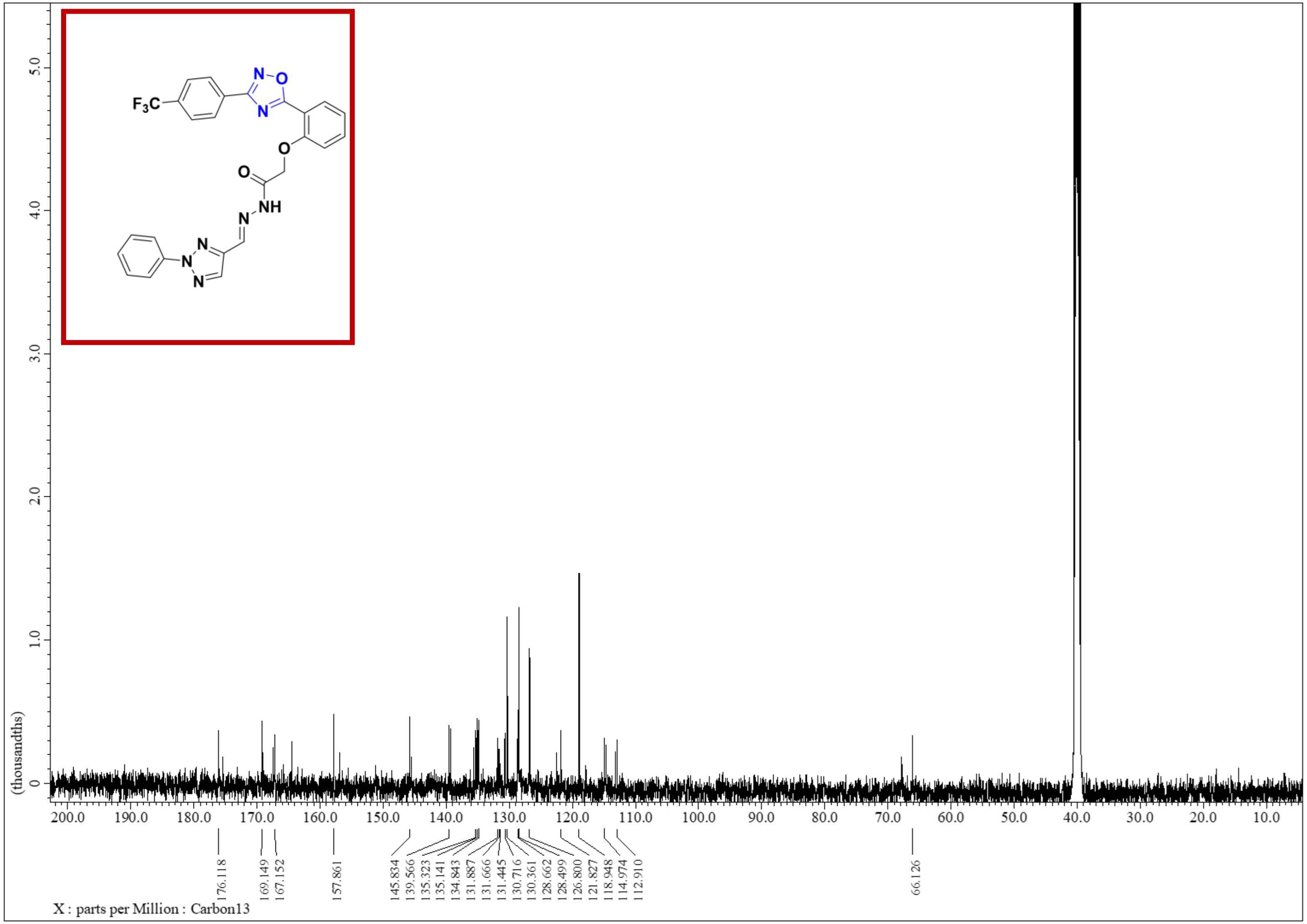

Fig. S12 ^{13}C-NMR spectrum (125 MHz, DMSO-d_6) of **3c.**

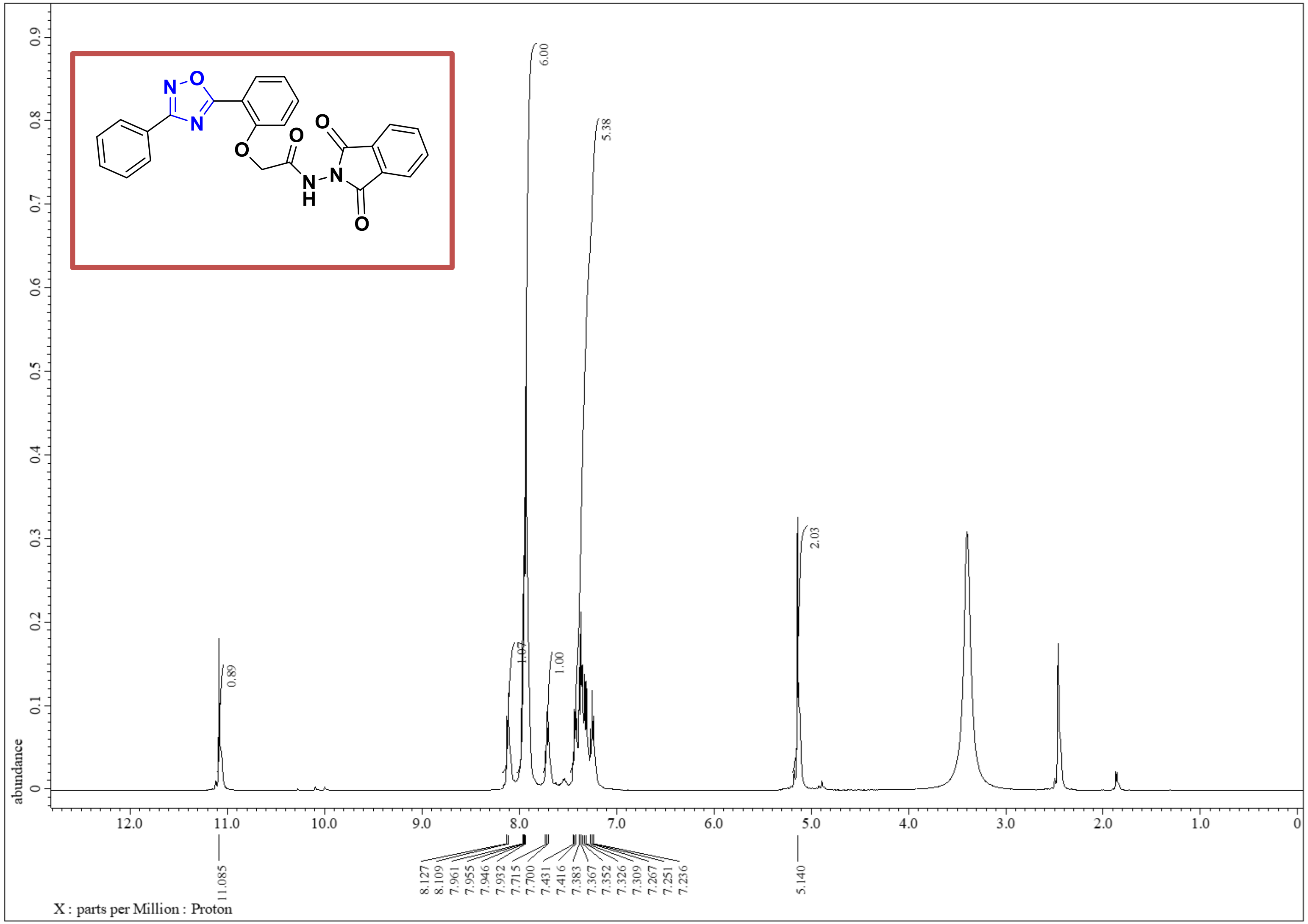

Fig. S13 ^{1}H-NMR spectrum (500 MHz, DMSO-d$_6$) of **4a.**

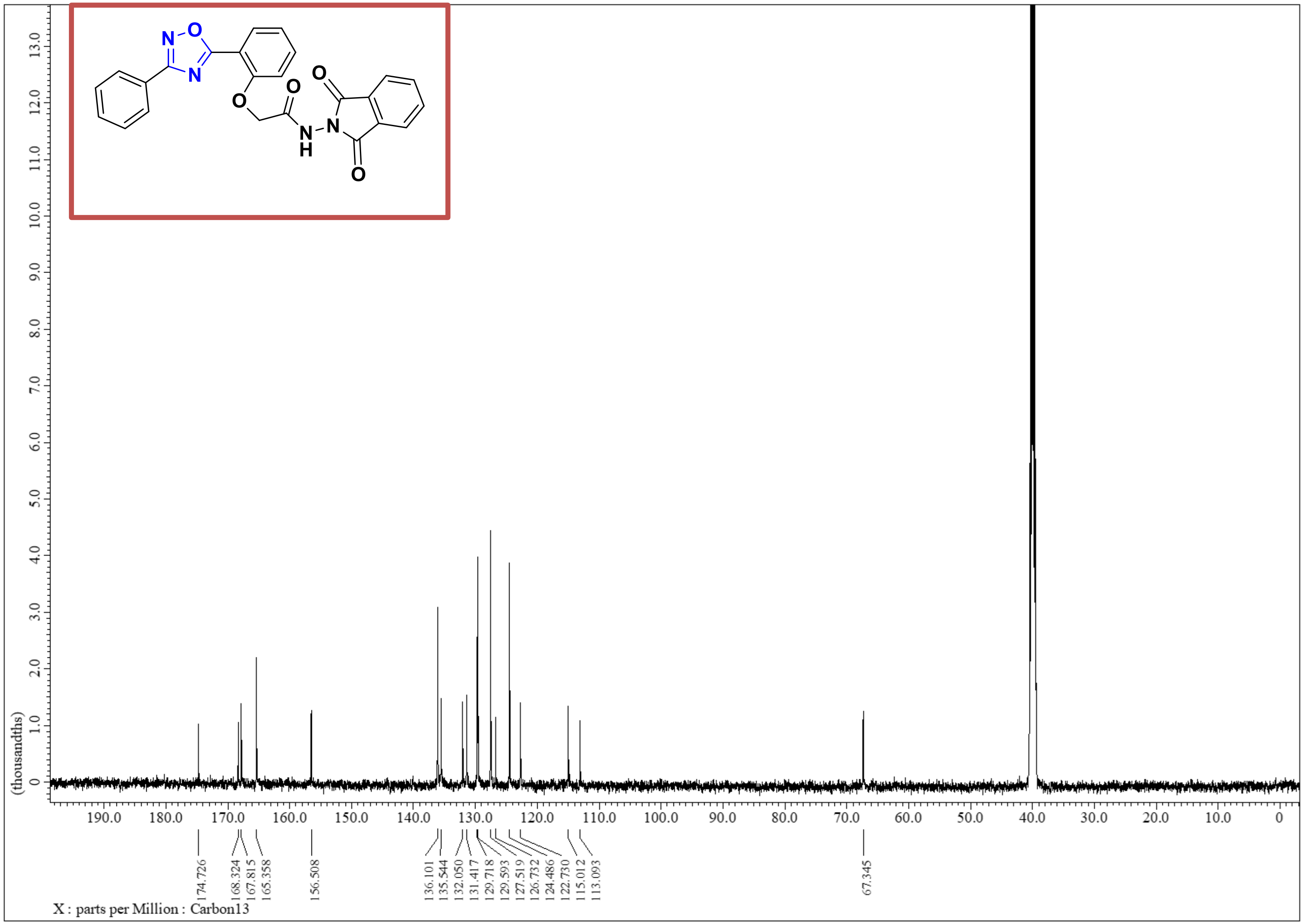

Fig. S14 ^{13}C-NMR spectrum (125 MHz, DMSO-d_6) of **4a.**

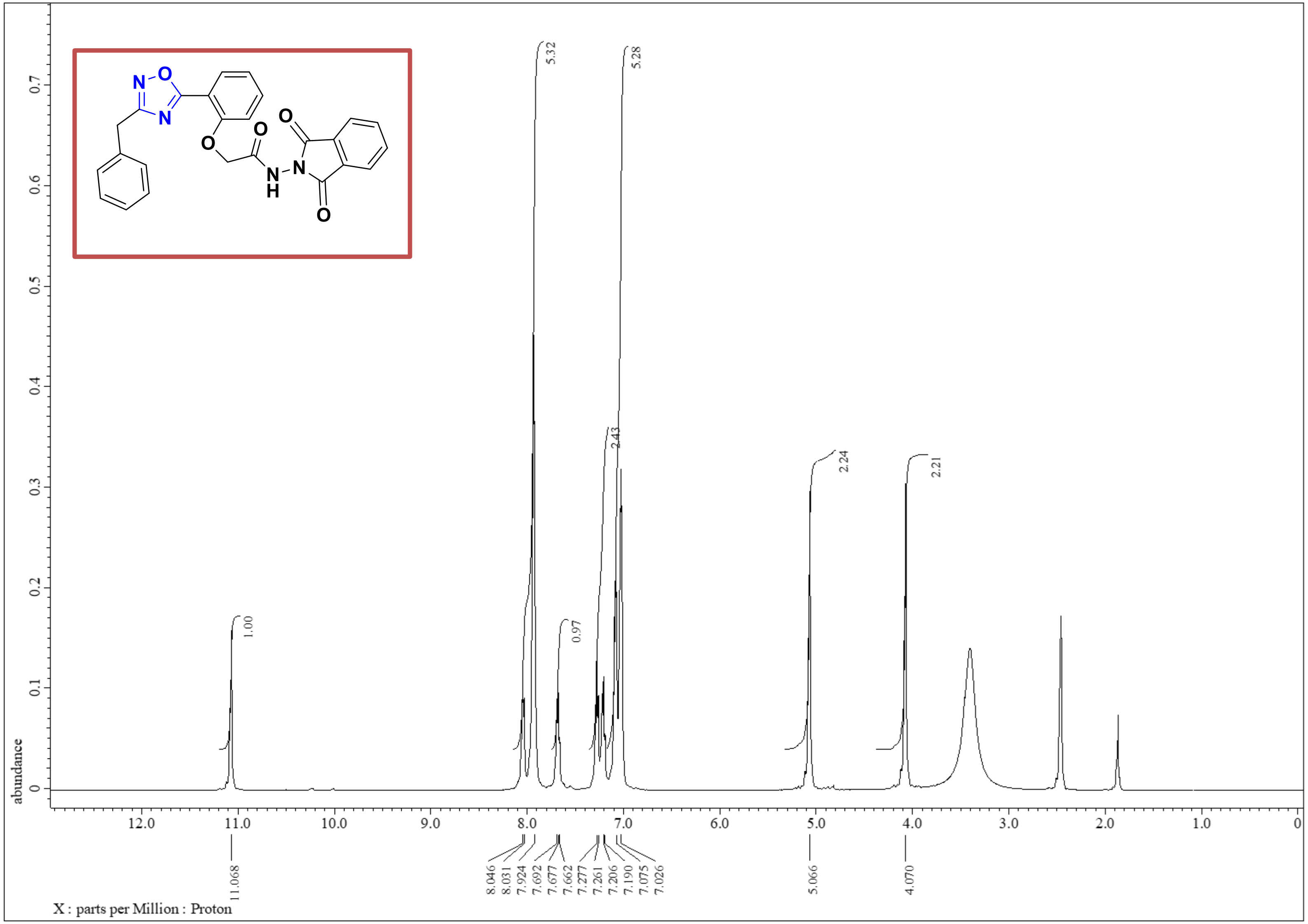

Fig. S15 ^{1}H-NMR spectrum (500 MHz, DMSO-d_6) of **4b.**

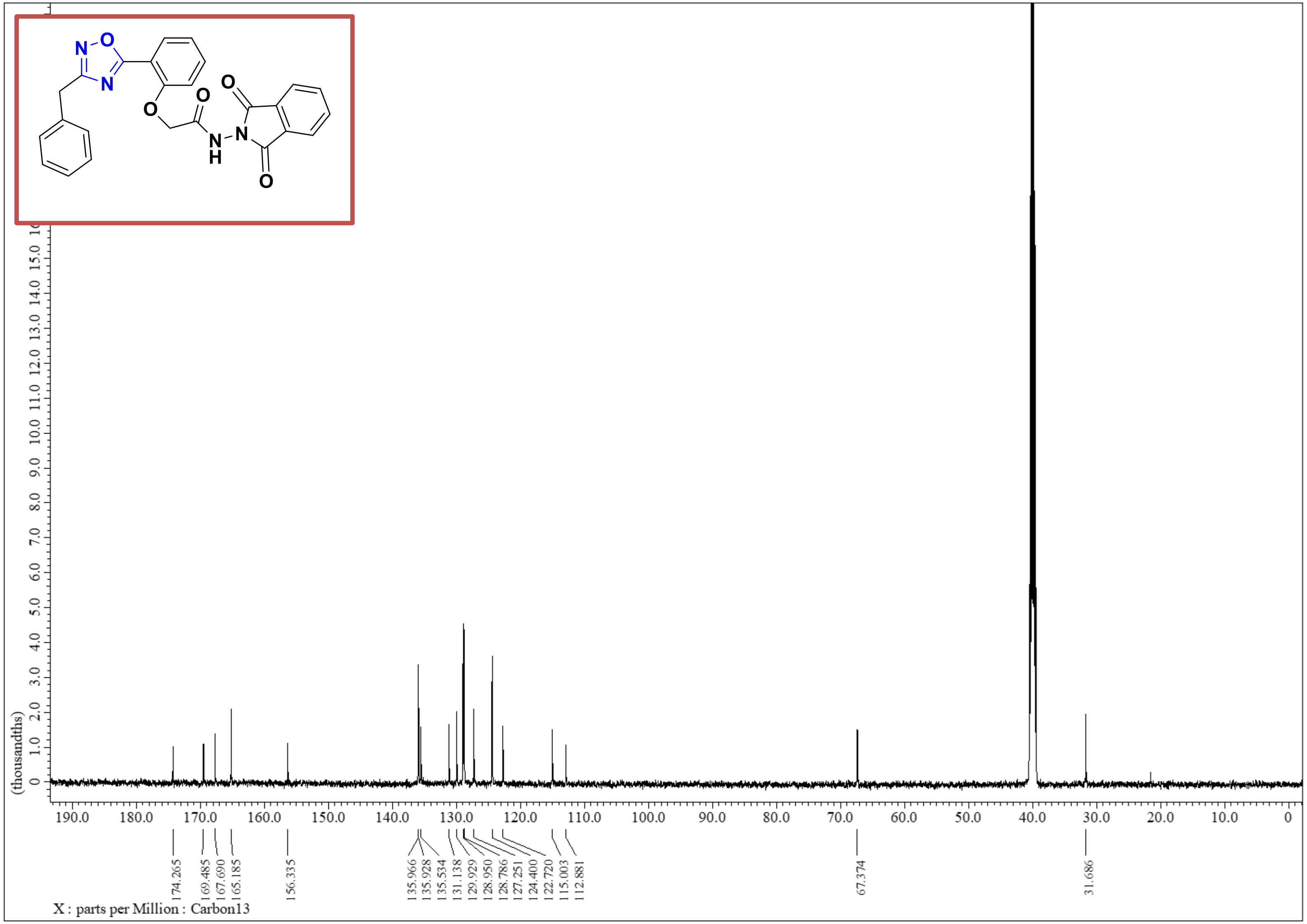

Fig. S16 ^{13}C-NMR spectrum (125 MHz, DMSO-d_6) of **4b.**

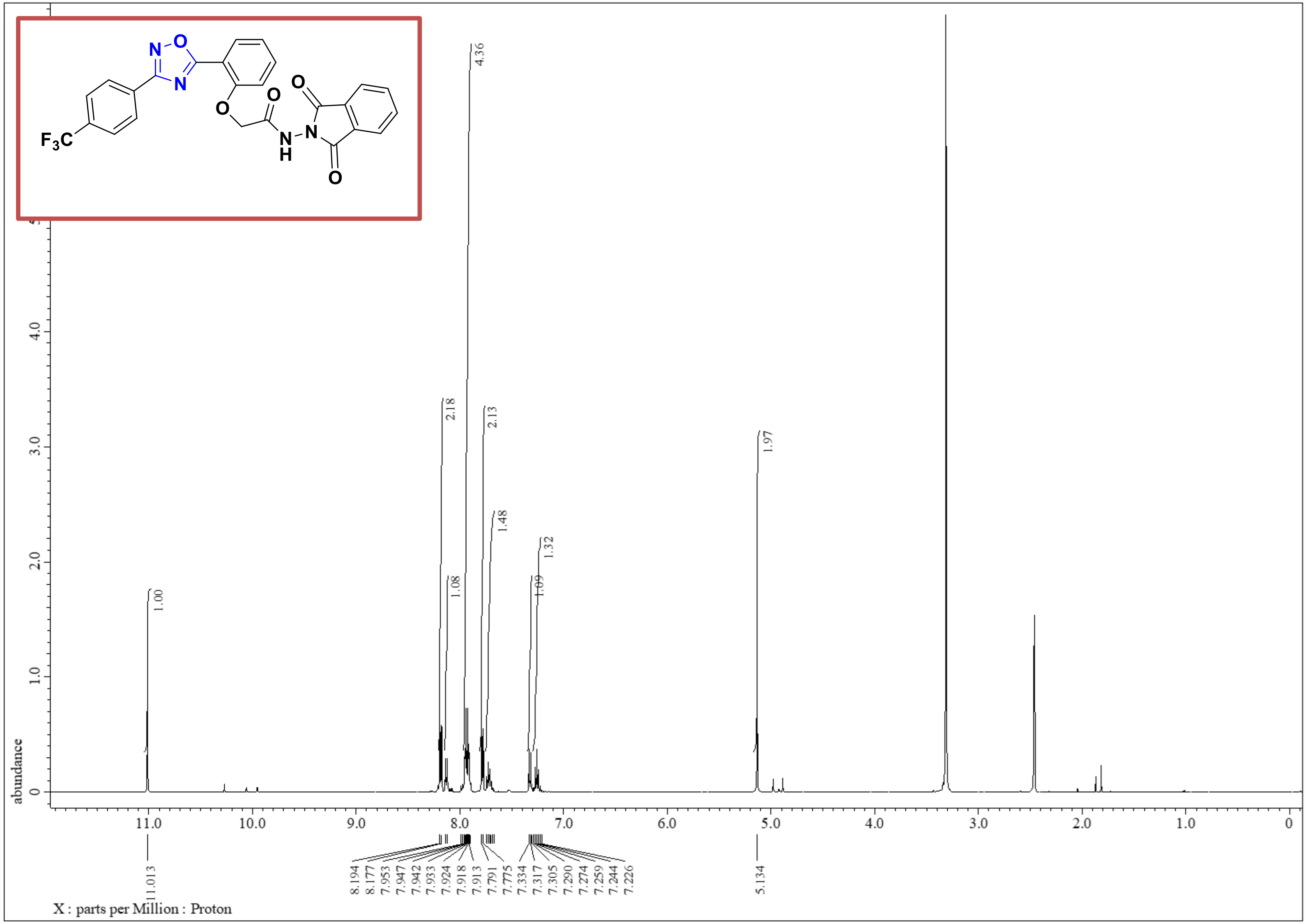

Fig. S17 ^{1}H-NMR spectrum (500 MHz, DMSO-d_6) of **4c.**

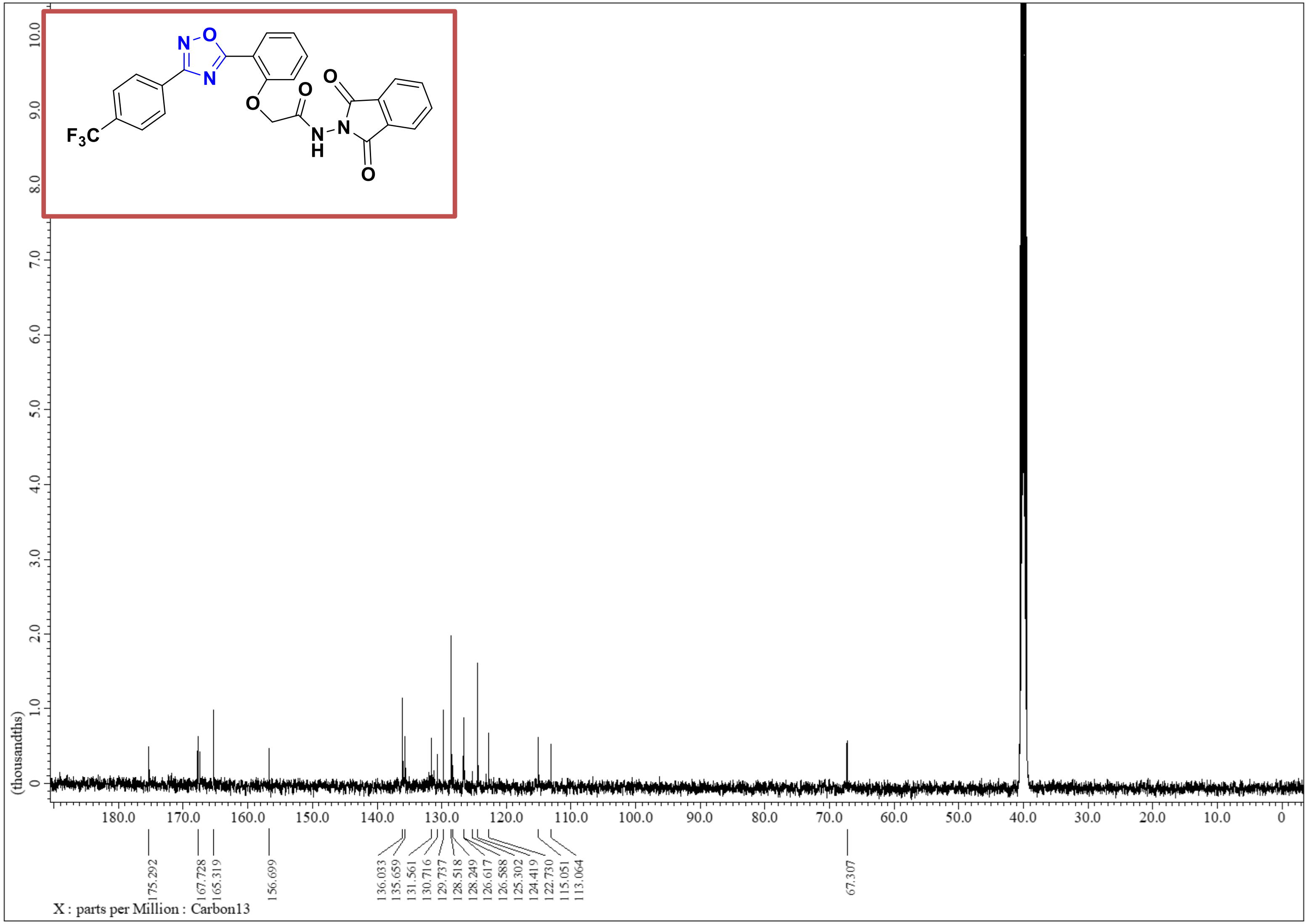

Fig. S18 ^{13}C-NMR spectrum (125 MHz, DMSO-d$_6$) of **4c.**

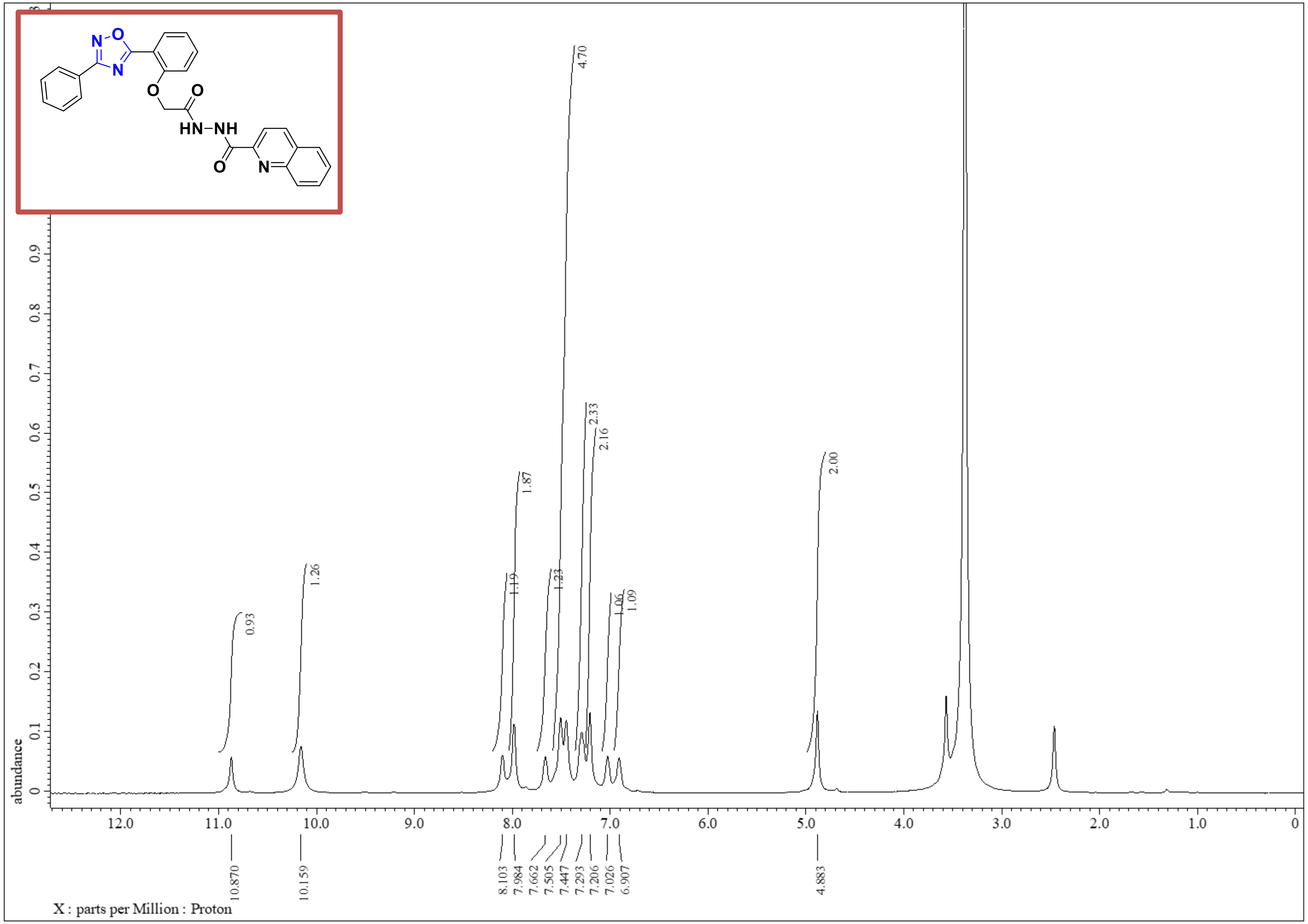

Fig. S19 ^{1}H-NMR spectrum (500 MHz, DMSO-d_6) of **5a.**

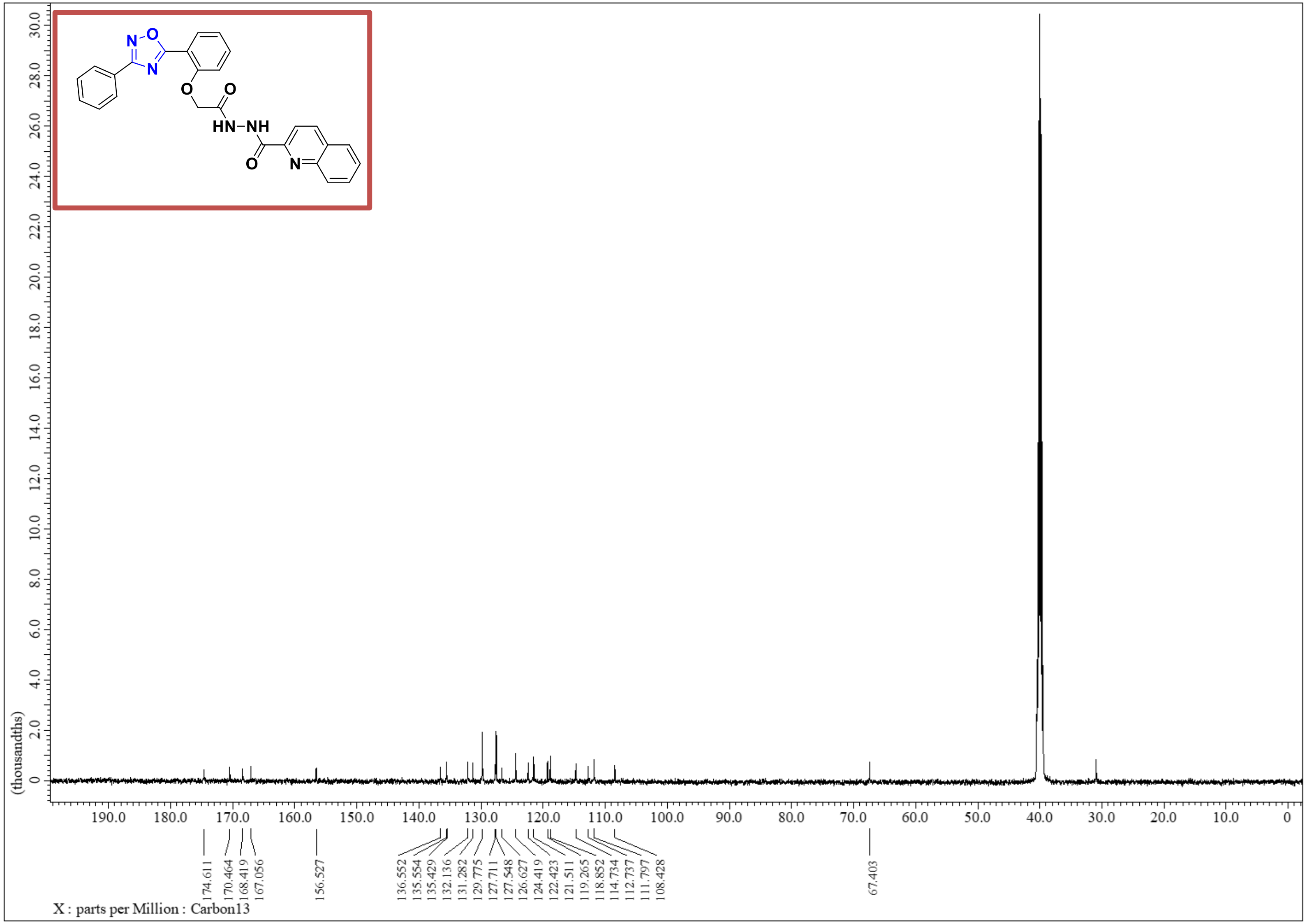

Fig. S20 ^{13}C-NMR spectrum (125 MHz, DMSO-d$_6$) of **5a.**

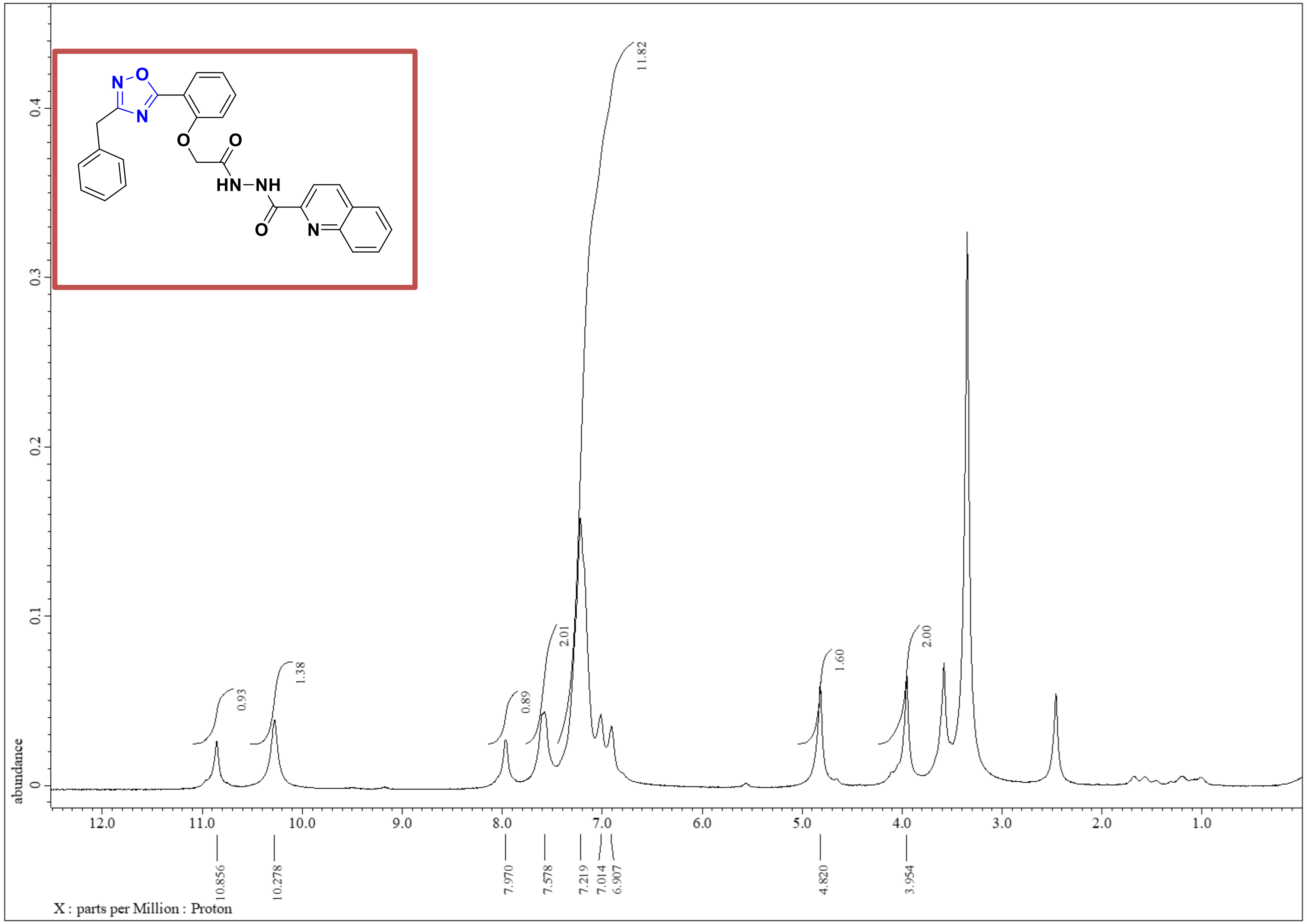

Fig. S21 ^{1}H-NMR spectrum (500 MHz, DMSO-d$_6$) of **5b.**

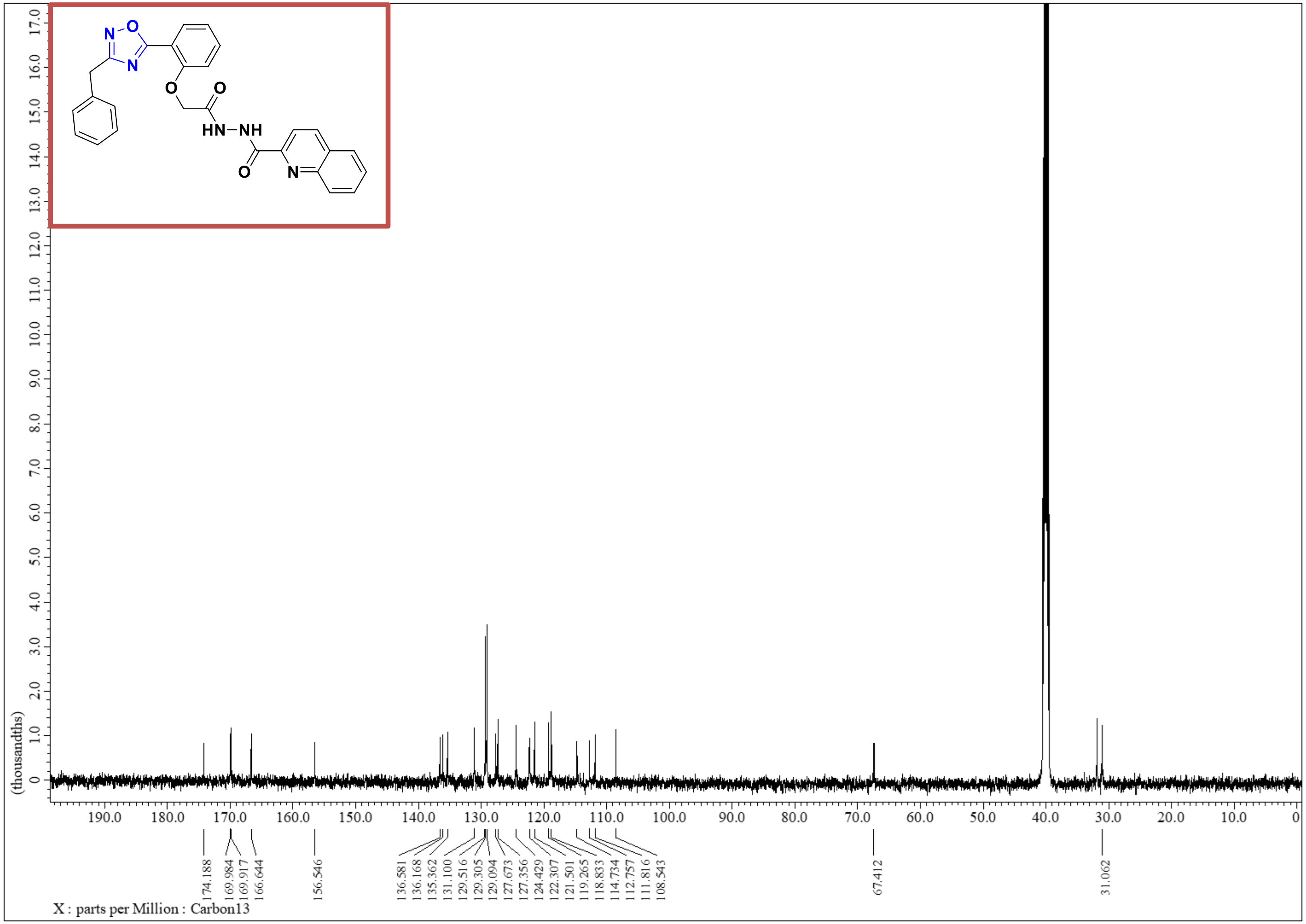

Fig. S22 ^{13}C-NMR spectrum (125 MHz, DMSO-d_6) of **5b.**

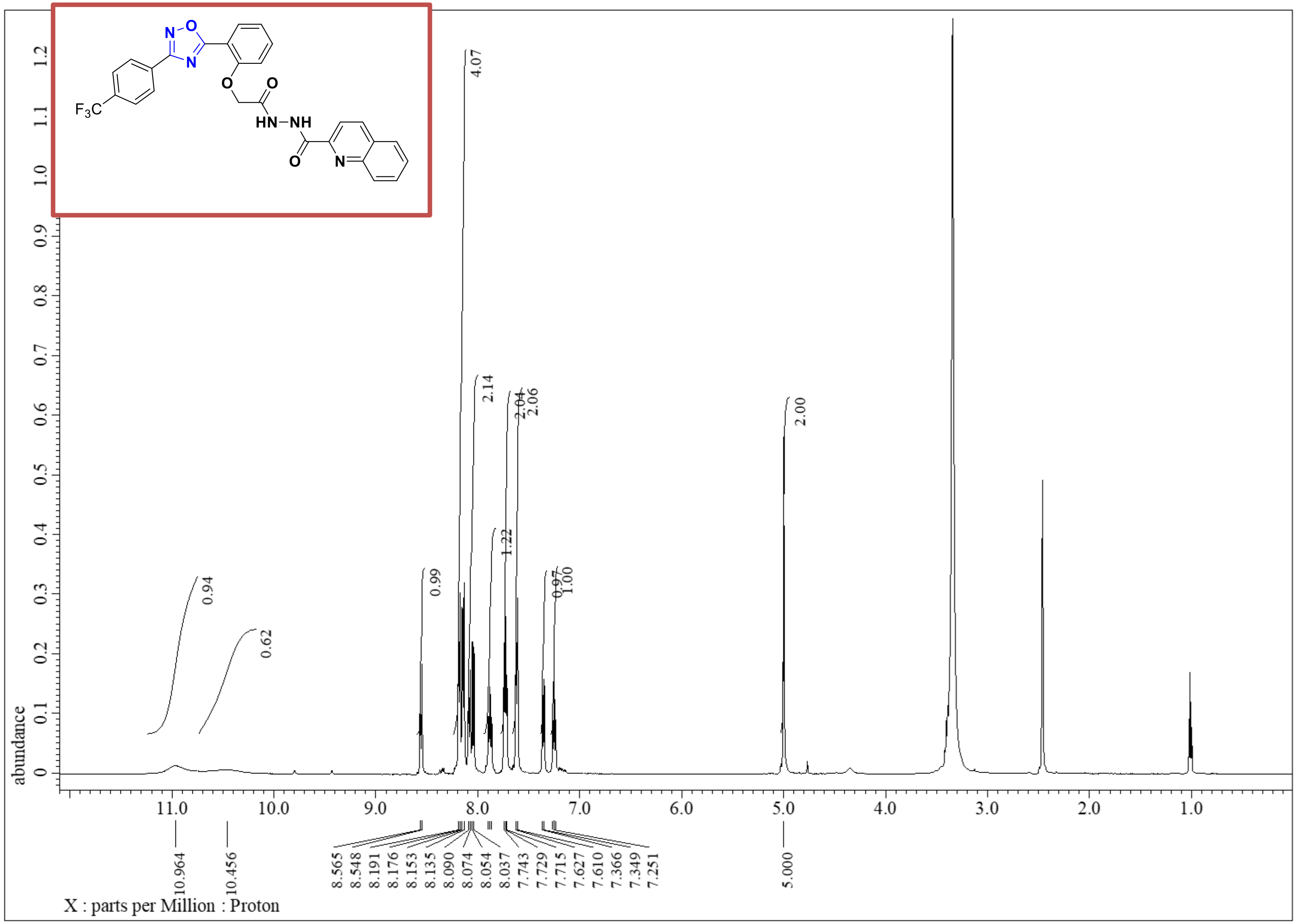

Fig. S23 ^{1}H-NMR spectrum (500 MHz, DMSO-d_6) of **5c.**

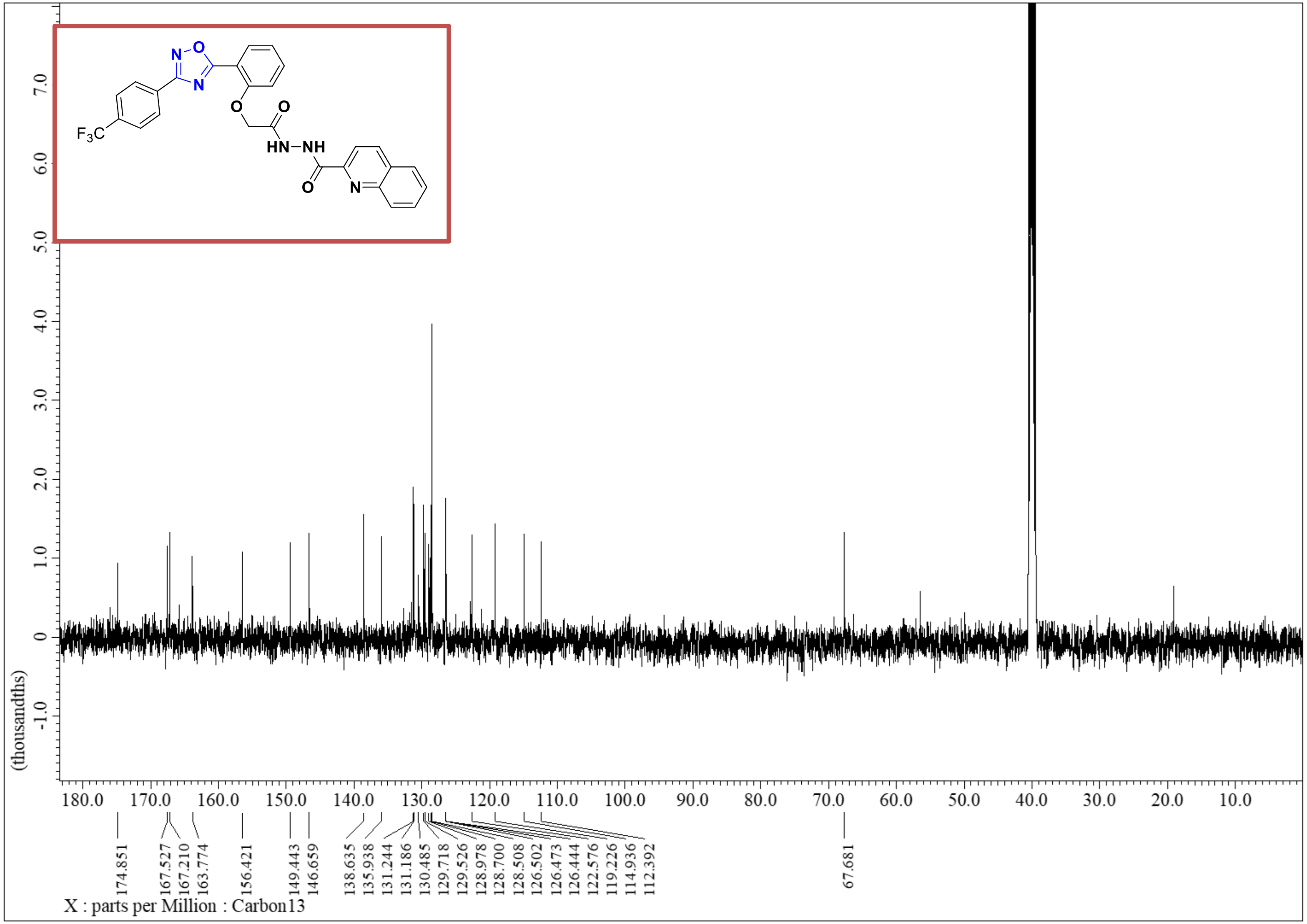

Fig. S24 ^{13}C-NMR spectrum (125 MHz, DMSO-d$_6$) of **5c.**

2. Equipment and analytical technique

All reactions were carried out in dried glassware. NMR spectra were measured using a JEOLJNM ECA 500. The deuterated solvent was used as an internal deuterium lock. ^{13}C NMR spectra were recorded using the UDEFT pulse sequence and broad band proton decoupling at 125 MHz. All chemical shifts (δ) are stated in units of parts per million (ppm) and presented using TMS as the standard reference point. CHN analyses were performed using a Flash 2000 organic elemental analyzer. IR (KBr) ν_{max} (cm^{-1}) data were recorded using PerkinElmer; FT-IR Spectrum BX and Bruker tensor 37 FT-IR. Reaction time was Melting points were recorded using Thermo Scientific, Model NO: 1002D, 220-240v; 200 W; 50/60 Hz, and are uncorrected. Reaction time was monitored by TLC on Merck silica gel aluminum cards (0.2 mm thickness) with a fluorescent indicator at 254 nm. Visualization of the TLC during monitoring of the reaction was done by UV VILBER LOURMAT 4w-365 nm or 254 nm tube.

3. Biological procedures

3.1. AChE and BuChE inhibitory assays[1]

In ELISA plate (Bio Tec. USA), 150 µL of phosphate buffer was directly added in ELISA blank well and 130 µL of phosphate buffer was added in ELISA activity wells. To the blank and activity wells, 5 µL of substrate ACTI was added, then 20 µL brain homogenate supernatant was added in activity ELISA wells only. The plate was preincubated for 15 min at 37 °C before the addition of the second substrate (DTNB). DTNB (60 µL) was added in both the blank and activity wells. Absorbance was measured at 405 nm every two min. Values obtained were analyzed and blank reading was subtracted from sample readings. The AChE and BuChE inhibition activity of tested compound was expressed as IC_{50}. IC_{50} value is the inhibitory concentration at which 50% of AChE or BuChE is repressed. It was calculated by interpolation from the graph of inhibition percentage against sample concentration using linear regression equations.

3.2 - 1,1-Diphenyl-2-picrylhydrazyl (DPPH) radical scavenging activity [2]

Antioxidant activity of tested compounds were assessed using DPPH. The assay mixture contained in a total volume of 100µL of prepared DPPH (0.004% in methanol) was added to 100 µL of sample (serial dilutions of tested compound). The plate was shaken to ensure thorough mixing before being wrapped with aluminum foil and placed into the dark for 30 min at 25°C. Then the decrease in absorbance was measured at 517 nm using optima spectrophotometer. The test carried out triplicate. The percent scavenging activity was calculated. The DPPH inhibition activity of tested compound was expressed as IC_{50}. IC_{50} value (mg/mL) is the inhibitory concentration at which 50% of DPPH is repressed. It was calculated by interpolation from the graph of inhibition percentage against sample concentration using linear regression equations.

3.3 MAO-A and MAO-B inhibitory assay [3]

In test tube, 150 µL of 20 nM of MAO-A or MAO-B (M7441, Sigma), 150 µL of tested compound serial dilutions (0, 0.1, 0.25, 0.5,1 and 2 mg/mL), 133 µL potassium phosphate buffer and 667 µL of or benzylamine (5µM) were mixed well. The absorbance was measured at 250 nm against air after 30 s and 90 s. The Inhibition percentage was calculated. IC_{50} value is the inhibitory concentration at which 50% of MAO-A or MAO-B is repressed. It was calculated by interpolation from the graph of inhibition percentage against sample concentration using linear regression equations.

3.4 The anti-hemolytic effect of synthesized compounds [4].

The human blood samples were collected in heparinized tubes and washed three times with isotonic buffered solution through centrifugation each time for 10 min at 3000 ×g. Membrane stabilizing activity of the extract was assessed using hypotonic solution-induced human erythrocyte hemolysis. The test sample consisted of stock erythrocyte (RBC) suspension (0.5 mL)

mixed with 5 mL of hypotonic solution containing serial concentration of the extracts (1, 2, 3 ,4 mg/mL). The control sample consisted of 0.5 mL of RBCs mixed with hypotonic-buffered saline solution alone. The mixtures were incubated for 10 min at room temperature and centrifuged for 10 min at 3000 ×g and the absorbance of the supernatant was measured at 540 nm using optima spectrophotometer. The test carried out triplicate. Inhibition of hemolysis percentages were calculated. Inhibition of hemolysis activity was expressed as IC_{50}. IC_{50} value (mg/mL) is the inhibitory concentration at which 50% of hemolysis are repressed. It was calculated by interpolation from the graph of inhibition percentage against sample concentration using linear regression equations.

4. Molecular modelling and ADME study

Table 1: 2D diagram and 3D representation of molecular docking of all synthesized compounds in the active site of AChE (PDB: 7E3H).

	2D interactions with human AChE active site (PDB: 7E3H)	3D interactions with human AChE active site (PDB: 7E3H)
**1a**	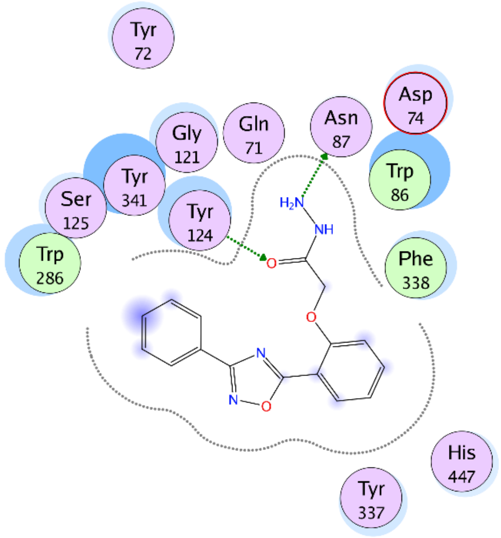	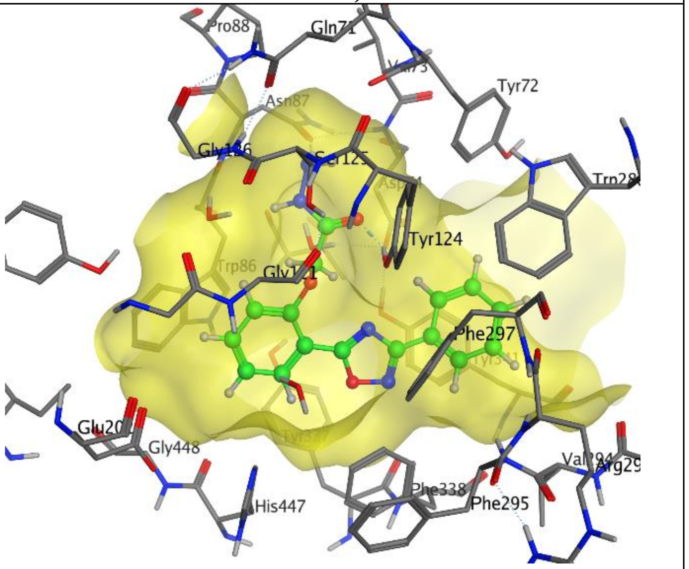

**1b**	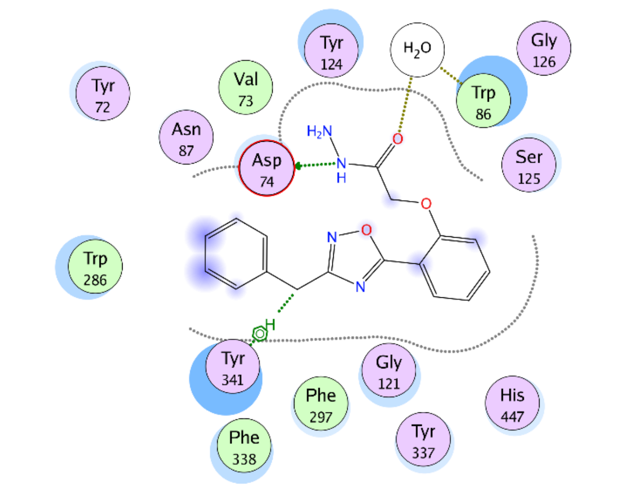Tyr 72<br>Val 73<br>Tyr 124<br>H2O<br>Gly 126<br>Trp 86<br>Asn 87<br>Asp 74<br>Ser 125<br>Trp 286<br>Tyr 341<br>Gly 121<br>Phe 297<br>His 447<br>Phe 338<br>Tyr 337	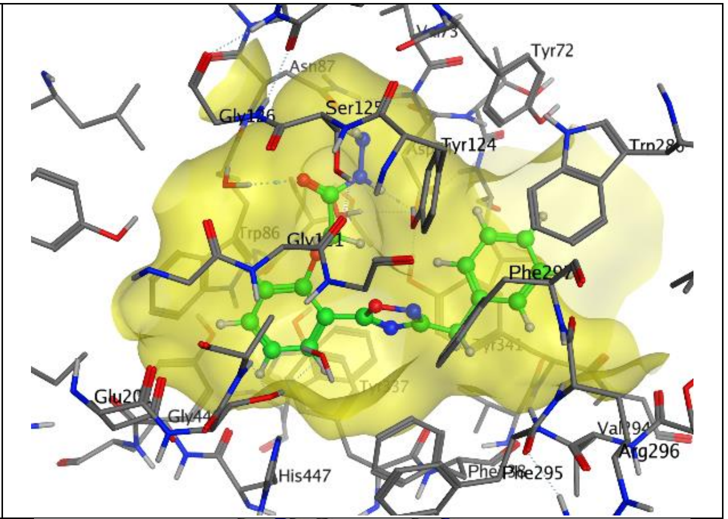Tyr72<br>Asn87<br>Ser125<br>Tyr124<br>Trp86<br>Phe297<br>Arg296<br>His447
**1c**	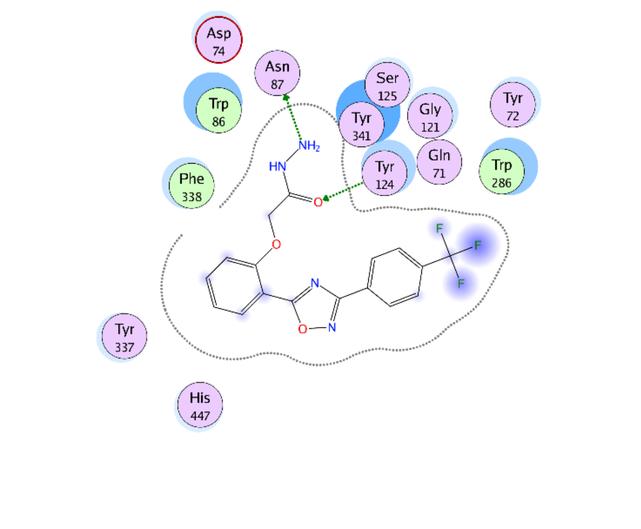Asp 74<br>Asn 87<br>Ser 125<br>Trp 86<br>Tyr 341<br>Gly 121<br>Tyr 72<br>Tyr 124<br>Gln 71<br>Trp 286<br>Phe 338<br>Tyr 337<br>His 447	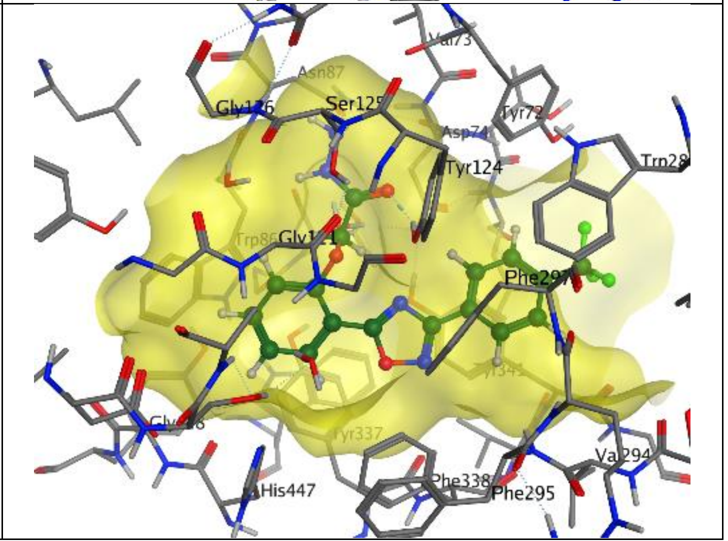Asn87<br>Gly126<br>Ser125<br>Tyr124<br>Phe297<br>Tyr337<br>Val294<br>Phe295<br>His447

**2a**	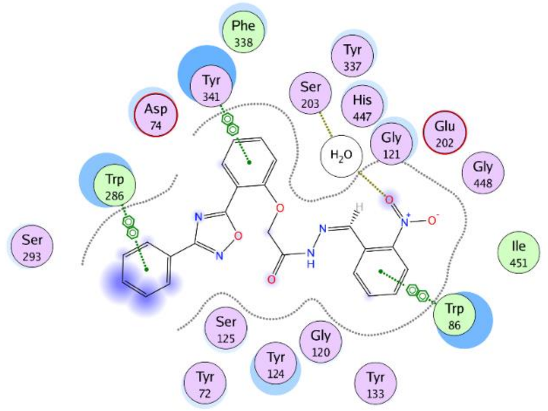Phe 338<br>Tyr 337<br>Tyr 341<br>Ser 203<br>His 447<br>Asp 74<br>Glu 202<br>Gly 121<br>Gly 448<br>H2O<br>Trp 286<br>Ser 293<br>Ile 451<br>Trp 86<br>Ser 125<br>Gly 120<br>Tyr 124<br>Tyr 72<br>Tyr 133	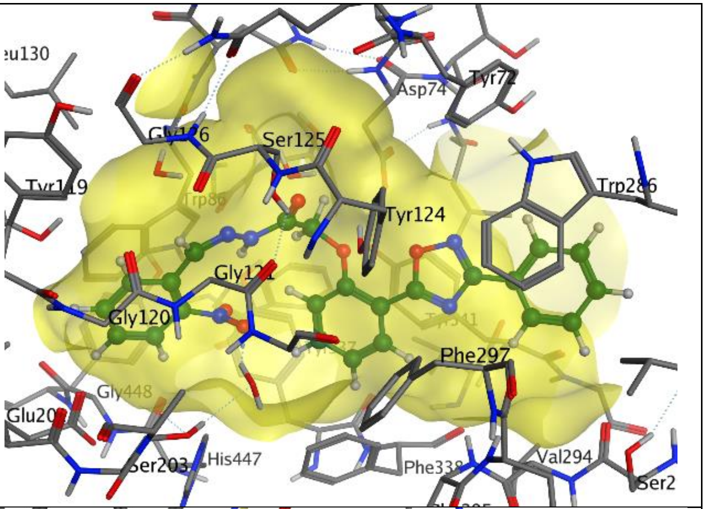Gly126<br>Ser125<br>Asp74<br>Tyr72<br>Trp286<br>Tyr124<br>Gly121<br>Gly120<br>Phe297<br>Gly448<br>His447<br>Phe338<br>Val294
**2b**	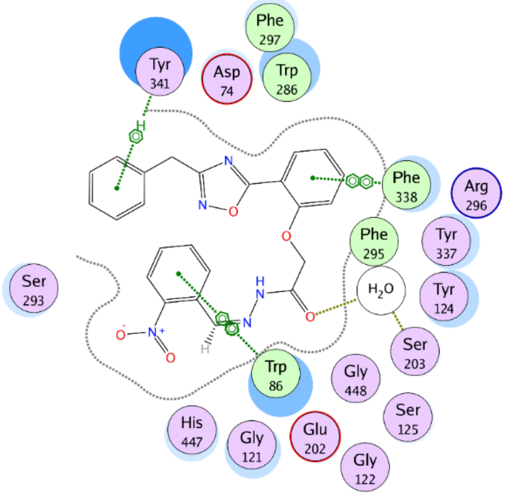Phe 297<br>Tyr 341<br>Asp 74<br>Trp 286<br>Phe 338<br>Arg 296<br>Phe 295<br>Tyr 337<br>Ser 293<br>H2O<br>Tyr 124<br>Ser 203<br>Trp 86<br>Gly 448<br>Ser 125<br>His 447<br>Gly 121<br>Glu 202<br>Gly 122	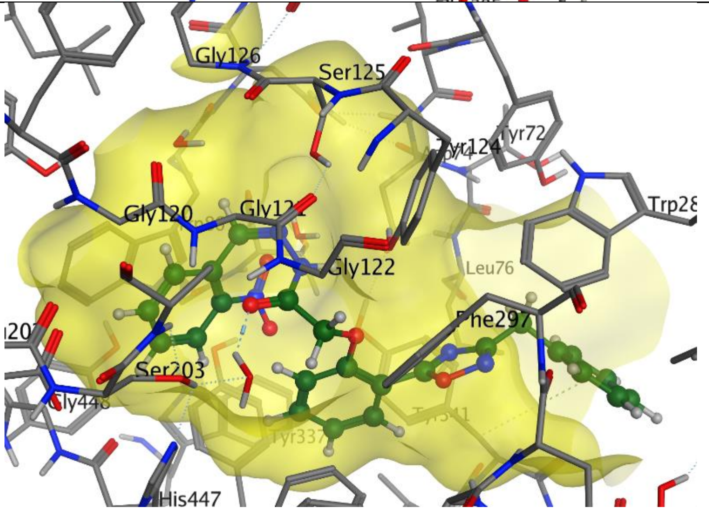Gly126<br>Ser125<br>Tyr72<br>Tyr124<br>Gly120<br>Gly122<br>Leu76<br>Phe297<br>Ser203<br>His447

2c

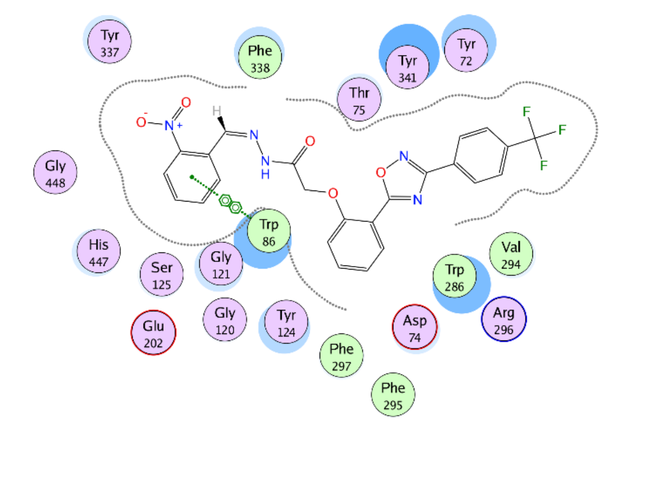
Tyr 337
Phe 338
Tyr 341
Tyr 72
Thr 75
Gly 448
His 447
Ser 125
Gly 121
Trp 86
Glu 202
Gly 120
Tyr 124
Phe 297
Phe 295
Asp 74
Trp 286
Val 294
Arg 296

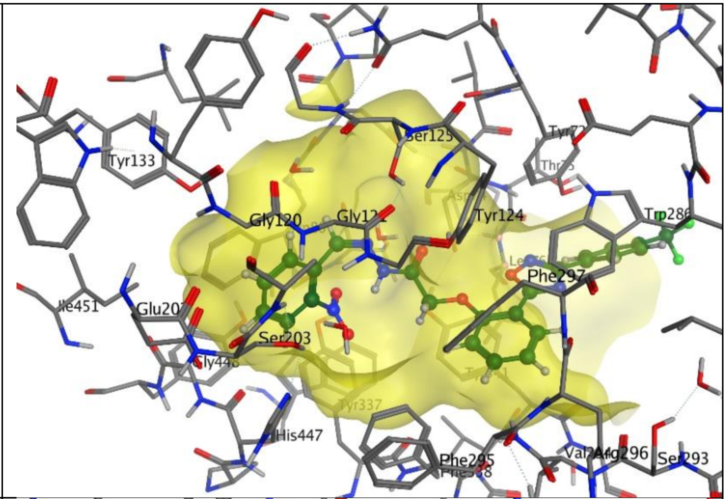
Tyr133
Ser125
Gly120
Tyr124
Trp286
Phe297
Glu202
Ser203
His447
Phe295
Ser293

3a

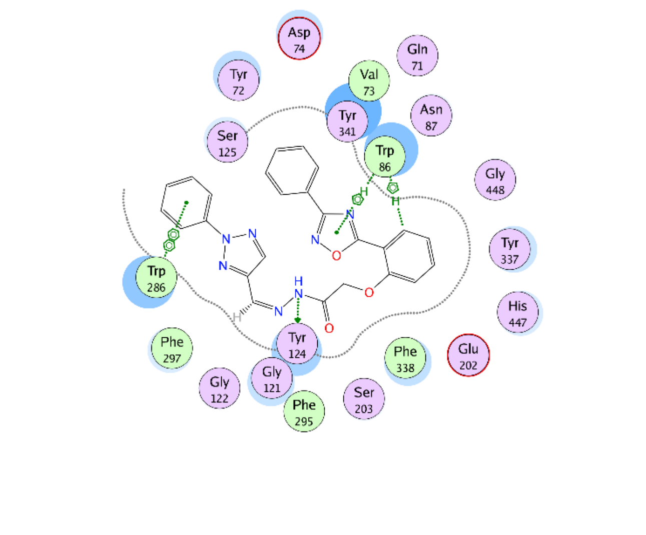
Asp 74
Gln 71
Tyr 72
Val 73
Tyr 341
Asn 87
Ser 125
Trp 86
Gly 448
Tyr 337
Trp 286
His 447
Phe 297
Tyr 124
Glu 202
Gly 122
Gly 121
Phe 338
Phe 295
Ser 203

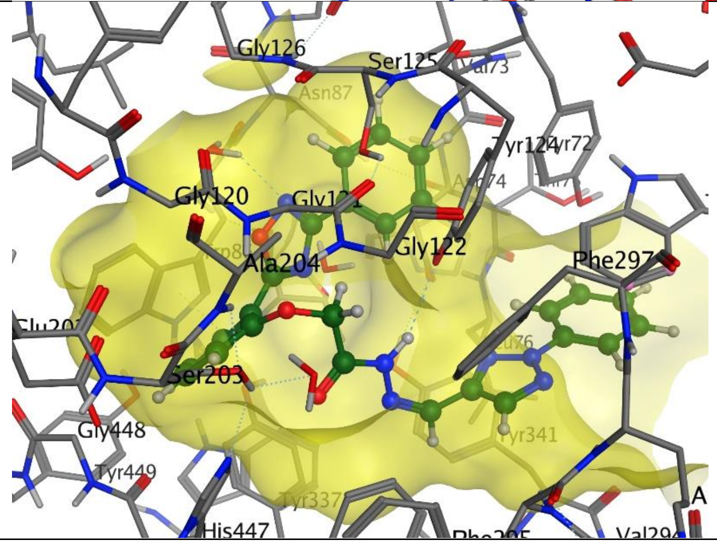
Gly126
Ser125
Asn87
Gly120
Gly122
Ala204
Phe297
Ser203
Gly448
Tyr449
Tyr341
His447

**3b**	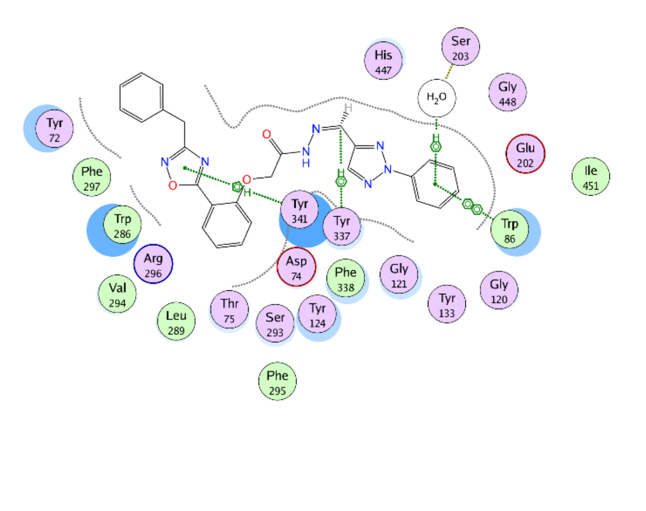 	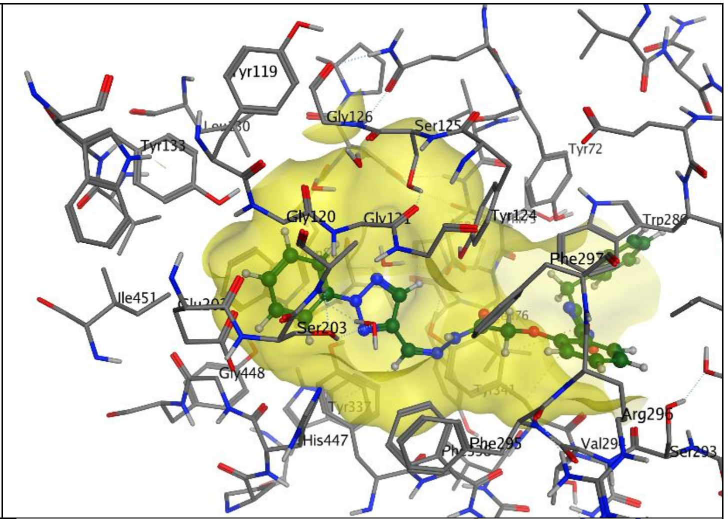
**3c**	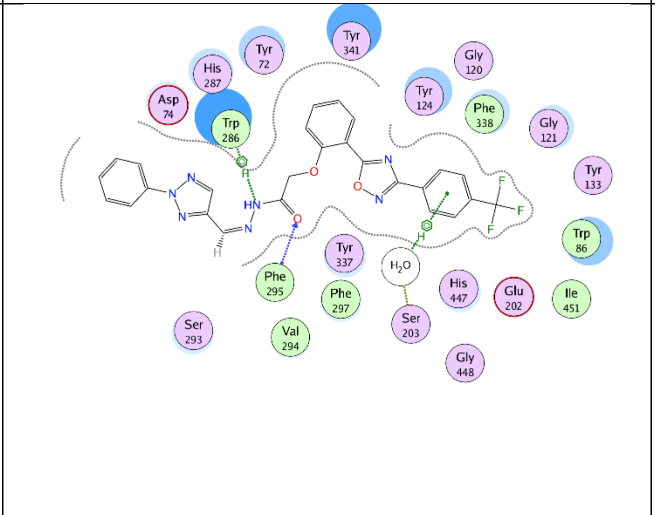 	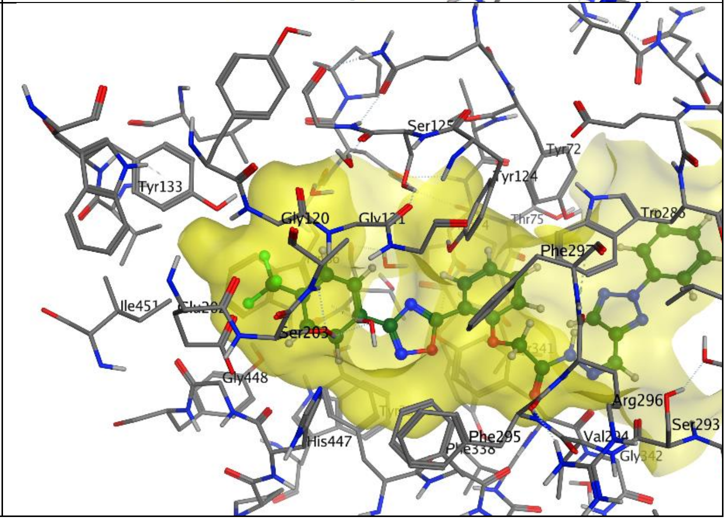

4a

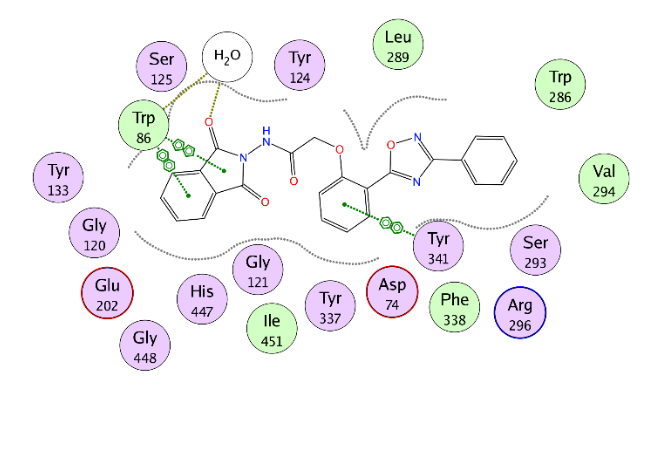
Ser 125
H_2O
Tyr 124
Leu 289
Trp 286
Trp 86
Tyr 133
Val 294
Gly 120
Tyr 341
Ser 293
Gly 121
Glu 202
His 447
Tyr 337
Asp 74
Phe 338
Arg 296
Ile 451
Gly 448

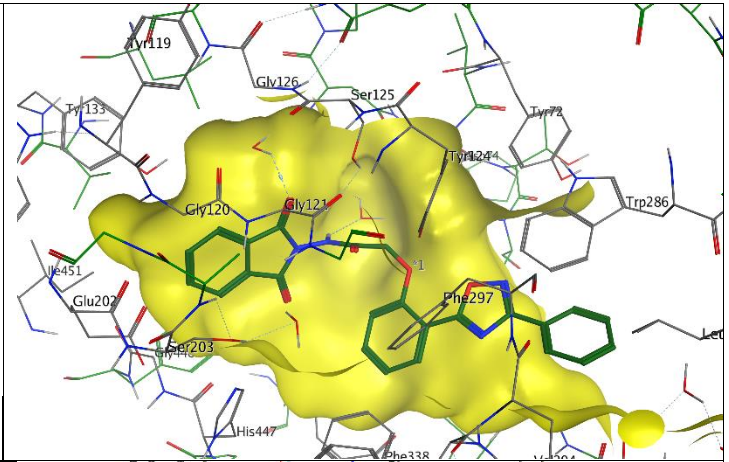
Tyr119
Gly126
Ser125
Tyr133
Tyr72
Gly120
Gly121
Trp286
Ile451
Glu202
Phe297
Ser203
His447

4b

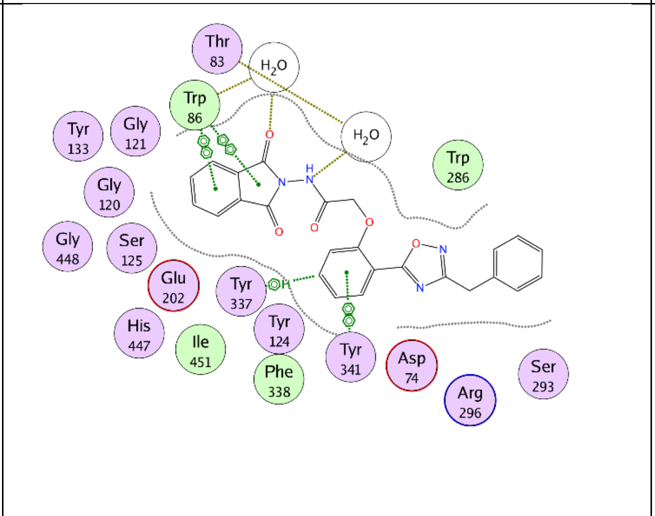
Thr 83
H_2O
Trp 86
H_2O
Tyr 133
Gly 121
Gly 120
Trp 286
Gly 448
Ser 125
Glu 202
Tyr 337
His 447
Tyr 124
Ile 451
Tyr 341
Asp 74
Phe 338
Arg 296
Ser 293

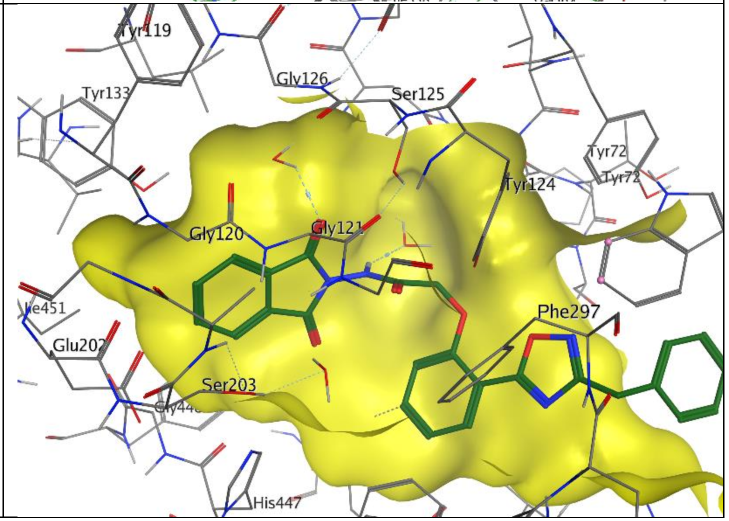
Tyr119
Gly126
Tyr133
Ser125
Tyr72
Tyr124
Gly120
Gly121
Ile451
Phe297
Glu202
Ser203
His447

4c

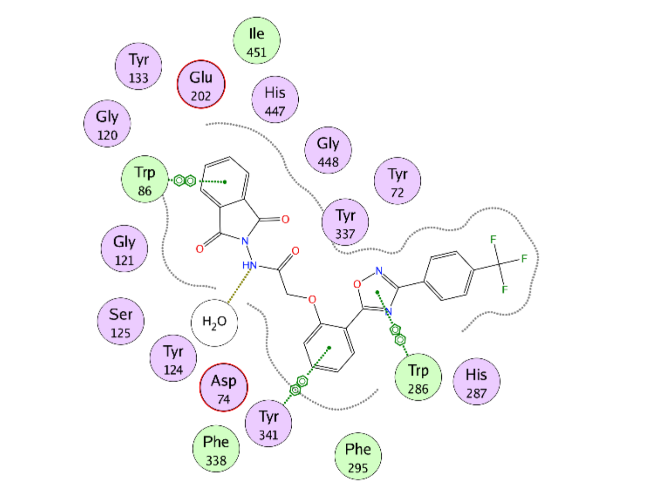
Ile 451
Tyr 133
Glu 202
His 447
Gly 120
Gly 448
Trp 86
Tyr 72
Tyr 337
Gly 121
HN
O
N
F
H2O
Ser 125
Tyr 124
Asp 74
Trp 286
His 287
Tyr 341
Phe 338
Phe 295

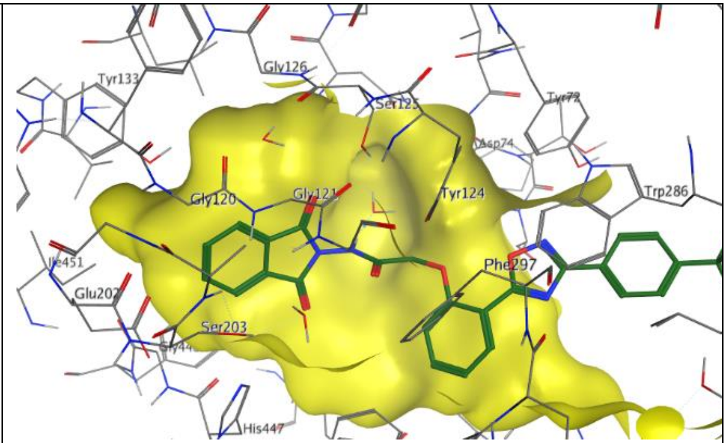
Tyr133
Gly126
Ser125
Tyr72
Asp74
Gly120
Gly121
Tyr124
Trp286
Phe297
Glu202
Ser203
His447

5a

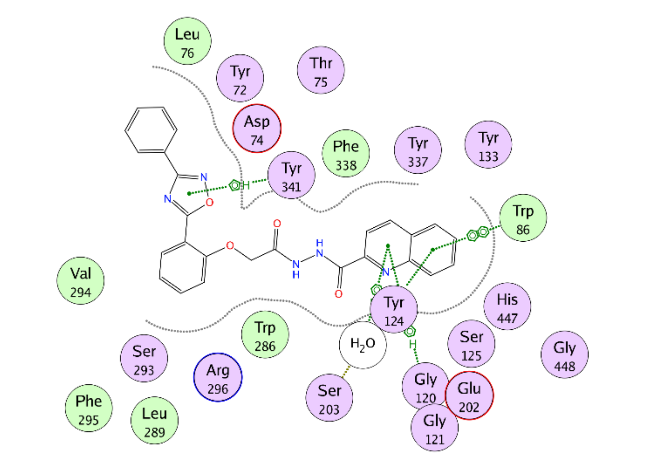
Leu 76
Tyr 72
Thr 75
Asp 74
Phe 338
Tyr 337
Tyr 133
Tyr 341
Trp 86
Val 294
H2O
Tyr 124
His 447
Trp 286
Ser 125
Gly 448
Ser 293
Arg 296
Ser 203
Gly 120
Glu 202
Gly 121
Phe 295
Leu 289

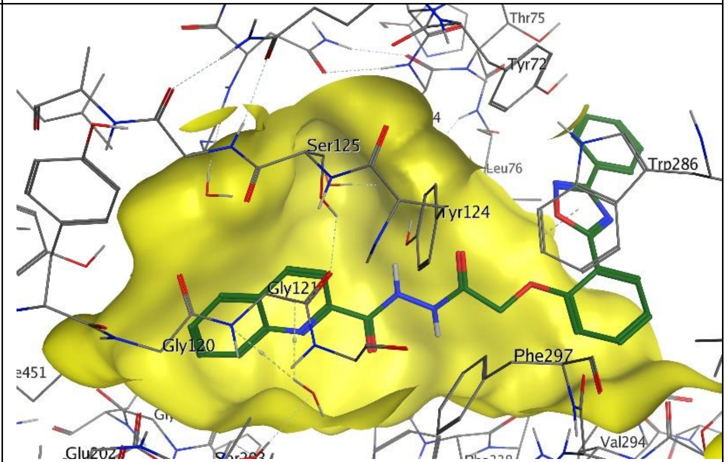
Thr75
Tyr72
Ser125
Leu76
Trp286
Tyr124
Gly121
Gly120
Phe297
Glu202
Val294

<table>
<tr><td>5b</td><td>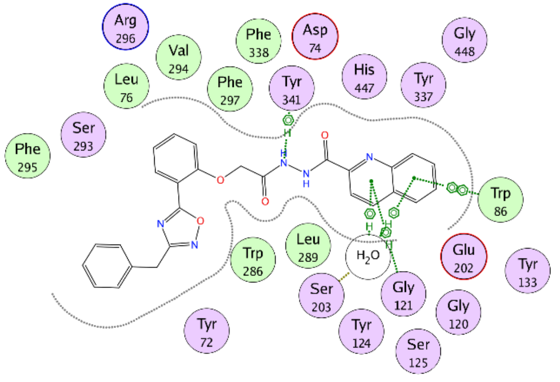</td><td>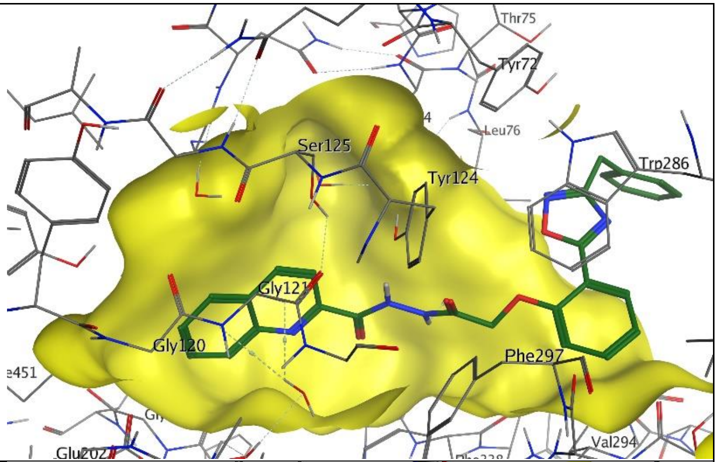</td></tr>
<tr><td>5c</td><td>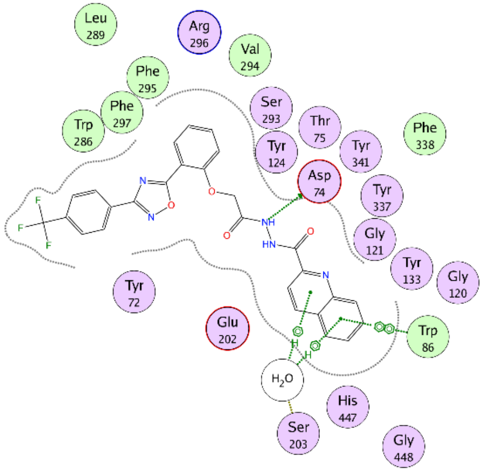</td><td>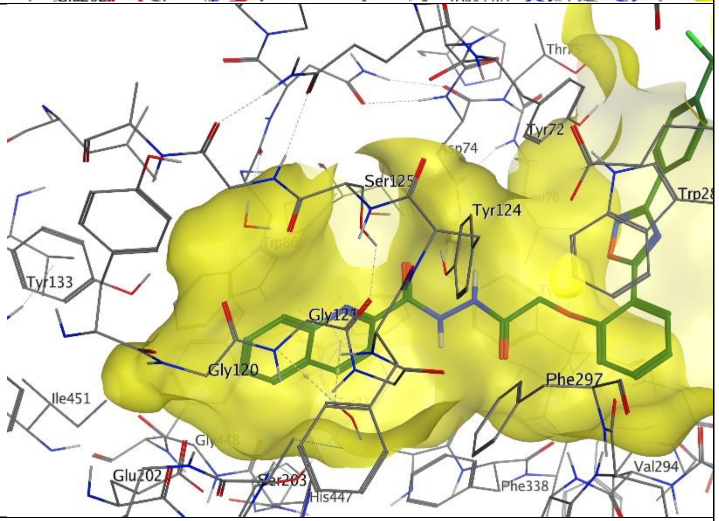</td></tr>
<tr><td></td><td></td><td></td></tr>
</table>

5. Table 2. Physicochemical properties and ADME properties of the target compounds.

Compd.	MW (g/mol) <500	#H-bond acceptors <10	#H-bond donors <5	TPSA ($Å^2$) <140	Log P o/w <5	GI absorption	Lipinski #violations	Bioavailability Score	PAINS #alerts
1a	310.3	6	2	103.3	1.73	High	0	0.55	0
1b	324.3	6	2	103.3	1.7	High	0	0.55	0
1c	378.3	9	2	103.3	2.19	High	0	0.55	0
2a	443.4	8	1	135.4	2.19	Low	0	0.55	0
2b	457.4	8	1	135.4	2.13	Low	0	0.55	0
2c	511.4	11	1	135.4	2.57	Low	1	0.55	0
3a	465.5	8	1	120.3	2.85	High	0	0.55	0
3b	479.5	8	1	120.3	2.78	High	0	0.55	0
3c	533.5	11	1	120.3	3.21	Low	1	0.55	0
4a	440.4	7	1	114.6	2.7	High	0	0.55	0
4b	454.4	7	1	114.6	2.91	High	0	0.55	0
4c	508.4	10	1	114.6	2.81	High	1	0.55	0
5a	465.5	7	2	119.2	2.6	High	0	0.55	0
5b	479.5	7	2	119.2	2.8	High	0	0.55	0
5c	533.5	10	2	119.2	2.96	Low	1	0.55	0
Donepezil HCl	379.49	4	0	38.77	3.06	High	0	0.55	0

6. References

[1] G.L. Ellman, K.D. Courtney, V. Andres, R.M. Featherstone, A new and rapid colorimetric determination of acetylcholinesterase activity, Biochem. Pharmacol. 7 (1961) 88–95. doi:10.1016/0006-2952(61)90145-9.

[2] A. Braca, N. De Tommasi, L. Di Bari, C. Pizza, M. Politi, I. Morelli, Antioxidant Principles from Bauhinia t arapotensis, J. Nat. Prod. 64 (2001) 892–895. doi:10.1021/np0100845.

[3] A. Szutowicz, R.D. Kobes, P.J. Orsulak, Colorimetric assay for monoamine oxidase in tissues using peroxidase and 2,2′-azinodi(3-ethylbenzthiazoline-6-sulfonic acid) as chromogen, Anal. Biochem. 138 (1984) 86–94. doi:10.1016/0003-2697(84)90773-5.

[4] O.O. Oyedapo, B.A. Akinpelu, K.F. Akinwunmi, M.O. Adeyinka, F.O. Sipeolu, Red blood cell membrane stabilizing potentials of extracts of Lantana camara and its fractions, Int. J. Plant Physiol. Biochem. 2 (2010) 46–51.
